# An effective Pd nanocatalyst in aqueous media: stilbene synthesis by Mizoroki–Heck coupling reaction under microwave irradiation

**DOI:** 10.3762/bjoc.13.166

**Published:** 2017-08-18

**Authors:** Carolina S García, Paula M Uberman, Sandra E Martín

**Affiliations:** 1 INFIQC-CONICET- Universidad Nacional de Córdoba, Departamento de Química Orgánica, Facultad de Ciencias Químicas, Haya de la Torre y Medina Allende, Ciudad Universitaria, X5000HUA, Córdoba, Argentina

**Keywords:** aqueous reaction medium, MAOS, Mizoroki–Heck reaction, Pd nanoparticle, sustainable organic synthesis

## Abstract

Aqueous Mizoroki–Heck coupling reactions under microwave irradiation (MW) were carried out with a colloidal Pd nanocatalyst stabilized with poly(*N*-vinylpyrrolidone) (PVP). Many stilbenes and novel heterostilbenes were achieved in good to excellent yields starting from aryl bromides and different olefins. The reaction was carried out in a short reaction time and with low catalyst loading, leading to high turnover frequency (TOFs of the order of 100 h^−1^). The advantages like operational simplicity, high robustness, efficiency and turnover frequency, the utilization of aqueous media and simple product work-up make this protocol a great option for stilbene syntheses by Mizoroki–Heck reaction.

## Introduction

Palladium-catalyzed reactions have emerged as an important tool for organic synthesis. Among them, cross-coupling and coupling reactions have been broadly applied for C–C and C–heteroatom bond formation [1–6]. These reactions have been proven to be powerful and attractive synthetic methods due to their high selectivity and tolerance to a wide range of functional groups on both coupling partners. The Mizoroki–Heck reaction and related chemistry occupy a special place among the basic types of Pd-catalyzed reactions, particularly the vinylation of aryl/vinyl halides or triflates [7–10], explored not only in the inter- and intramolecular version [2,11–13], but also in domino [14] and asymmetric catalytic processes [15]. Due to its versatility, the Mizoroki–Heck reaction is extensively employed in the synthesis of pharmaceutical, agrochemical and natural products, and continues to attract attention within the synthetic chemists’ community [16–18].

From an economic and environmental standpoint, it is highly desirable to develop more environmentally friendly synthetic methodologies. Several studies were dedicated to the design of a novel catalytic system, that are more accessible, robust, efficient and ligand-free [19]. In that sense, nanoparticles (NPs) of transition metals have been introduced as eco-catalysts in several reactions [20]. The NPs became a useful tool for catalytic transformations due to their excellent performance, which is related to their small size and a high surface-to-volume ratio [21–25]. Moreover, they are relatively easy to prepare and can be obtained in different size and morphology by essentially controlling the synthetic method and experimental conditions. As a result of the excellent catalytic activity of NPs, they can be used under mild and, in some cases, in environmental sustainable conditions. In particular, Pd NPs have been well-established as catalysts in the Mizoroki–Heck coupling reaction, obtaining the desired products in good to high yields, finding some examples in aqueous solution and under microwave (MW) irradiation [26–36]. Besides these studies, there is considerable room for improvements of the reported catalytic systems, which may involve high catalyst loadings (>1 mol %), toxic solvents, or limited application scope of iodoarenes and olefins [19].

Along with the increasing awareness about the development of sustainable catalysts, additional efforts should be made to address also sustainable reaction conditions; like employing ecological solvents and alternative energy input among others aspects [37–38]. Water represents a very attractive medium as a result of its advantages as solvent (nontoxic, not flammable, cheap and readily available), the use of water or aqueous systems as solvent in organic synthesis to replace hazardous and expensive organic solvents have been explored [39–40]. Various catalytic systems have been reported for the Mizoroki–Heck reaction in aqueous medium [41–43]. However, a drawback of using water is related to the limited solubility of the reactants, leading to less efficient reactions. To solve this problem several systems have been developed by using co-solvents, surfactants, pressure reactors or heating the reaction mixture by MW irradiation [44]. Under MW irradiation conditions, not only the rate of organic reactions could be increased, and better selectivities of the products achieved, but also the homogeneous mixing of the reactants in water could be enhanced, on the basic of the efficient ability to absorb MW energy by water [44]. The use of water in combination with MW heating for Heck coupling reactions has been reported, using a phase-transfer agent and focusing on the employment of ultralow catalyst concentrations [45].

Recently, we reported the electrochemical synthesis of stable Pd NPs by using platinum or vitreous carbon electrodes at room temperature via direct electroreduction of aqueous H_2_PdCl_4_ in the presence of poly(*N*-vinylpirrolidone) (PVP) [46–47]. These Pd NPs exhibited highly efficient catalytic activity in Suzuki–Miyaura coupling reactions [46–47] and nitroaromatic hydrogenations [48] in aqueous medium. Outstanding performance of these PVP-Pd NPs in the coupling reaction was observed, reaching high turnover numbers (TON up 10^4^–10^5^) in the presence of aryl iodides and bromides. Additionally, the performance of the NPs was briefly examined for the Mizoroki–Heck reaction with haloacetophenones [47]. As a remarkable feature of this nanocatalyst, besides its great catalyst activity and compatibility with aqueous reaction media, is that it can be used without previous purification processes. Consequently, as a part of our ongoing interest in developing green reaction conditions for organic syntheses; we herein report an extensive study on the catalytic application of the electrochemical synthesized PVP-Pd NPs for Mizoroki–Heck coupling reaction. By the efficient coupling reaction with aryl bromides, many stilbenes and novel hetero-stilbenes were obtained employing the Pd NPs in aqueous medium under relatively mild conditions, using MW irradiation.

## Results and Discussion

The PVP-Pd NPs of a mean diameter of 10 nm were obtained using a platinum electrode, characterized as previously reported, and used without additional purification [46–47]. In a previous work the PVP-Pd NPs synthesized using a vitreous carbon electrode were tested on the Mizoroki–Heck reaction with 4-iodo- or 4-bromoacetophenone and styrene [47]. The best result with 4-bromoacetophenone was accomplished when the reaction was performed under MW irradiation for 10 minutes using 0.2 mol % of Pd, affording the (*E*)-stilbene product in 98% yield. In order to further explore the scope of the PVP-Pd NPs-catalyzed Mizoroki–Heck coupling, a systematic study of the reaction was carried out with a wide range of substrates and olefins. Based on our previous results [47], and other reported examples of Mizoroki–Heck coupling reactions that use MW irradiation as alternative energy input [26,28,36,49], the reaction of 4-bromoacetophenone (**1a**) and styrene (**2a**) as coupling reagents was initially selected to screening the reaction conditions (Table 1). In this case, the reactions were performed with 0.1 mol % of Pd, in a mixture of H_2_O/alcohol (3:1) as solvent under MW irradiation, employing a dynamic heating method at fixed temperature in a sealed vessel. The results are summarized in Table 1.

**Table 1:** Reaction condition optimizations.^a^

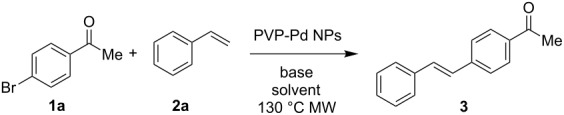				

Entry	Solvent	Base	*t* (min)	Yield (%)^b^

1	H_2_O/EtOH	K_2_CO_3_	1	65
2	H_2_O/EtOH	K_2_CO_3_	5	92
3	H_2_O/EtOH	K_2_CO_3_	10	100
4^c^	H_2_O/EtOH	K_2_CO_3_	10	88
5	H_2_O	K_2_CO_3_	10	23
6	H_2_O/iPrOH	K_2_CO_3_	10	100
7	H_2_O/ethylene glycol	K_2_CO_3_	10	54
8^d^	H_2_O/EtOH	K_2_CO_3_	10	100
9	H_2_O/EtOH	Na_2_CO_3_	10	100
10	H_2_O/EtOH	K_3_PO_4_	10	93
11	H_2_O/EtOH	NaOAc	10	54
12	H_2_O/EtOH	–	10	<5
13^e^	H_2_O/EtOH	K_2_CO_3_	10	73

^a^Reaction conditions: 0.25 mmol of 4-bromoacetophenone (**1a**), 0.30 mmol of styrene (**2a**), 0.5 mmol of the base, 0.1 mol % of Pd, 2 mL of the mixed solvent 3:1, 130 °C MW (dynamic heating method) in a sealed vessel. ^b^GC yields. The yields reported represent at least the average of two reactions. ^c^Pd loading of 0.05 mol %. ^d^Tap water. ^e^Catalyst source: H_2_PdCl_4_ 0.5 mM + 16 g/L PVP.

A general screening was performed to determine the optimal reaction time, the identity and presence of a co-solvent (ethanol, isopropanol, ethylene glycol) and the best base (K_2_CO_3_, Na_2_CO_3_, K_3_PO_4_, sodium acetate (NaOAc)). In a first approach, time optimization was performed by using K_2_CO_3_ as base, at 130 °C by MW irradiation (entries 1–3, Table 1). The highest catalytic activity of PVP-Pd NPs was obtained after 10 minutes, when the yield of stilbene **3** achieved 100% (entry 3, Table 1). Reducing the catalyst loading (0.05 mol %) leads to a decrease of product yield, however, to a very respectable 88% yield (entry 4, Table 1). To achieve a complete conversion with this Pd loading 20 min of MW irradiation were required. The influence and identity of the co-solvent were also explored (entries 5–8, Table 1). This screening showed that the presence of an alcohol was required to obtain the products in high yields (entry 5, Table 1). Ethanol or isopropanol can be used in this system to achieve quantitatively the desired products; however, when ethylene glycol was used lower yields were reached (entries 3, 6 and 7, Table 1). On the other hand, the use of tap water had no negative effect on the catalytic activity of the catalyst (entry 8, Table 1). When the nature of the base was evaluated, it was observed that inorganic bases gave high yields of stilbene **3** (entries 9 –12, Table 1). K_2_CO_3_ and Na_2_CO_3_ could be used to obtain the products in excellent yields (entries 3 and 9, Table 1). A slightly lower yield of stilbene **3** was observed employing K_3_PO_4_ (93%; entry 10, Table 1). As it was expected, when the reaction was carried out in the absence of a base, there was no conversion of substrate **1a** (entry 12, Table 1).

Taking into account that several Pd NPs could be synthesized by MW irradiation [49], the in situ formation of NPs in the system was evaluated. For that, the Mizoroki–Heck coupling reaction was carried out with a homogeneous mixture based on H_2_PdCl_4_ and PVP (the same solution employed to obtain PVP-Pd NPs by electroreduction, entry 13, Table 1). Under the same reaction conditions, this in situ arranged catalytic system proved to be less active than the electrochemically preformed Pd NPs. Additionally, Pd NPs of an average size of (4 ± 1) nm and spherical shape were obtained when they were prepared from the same H_2_PdCl_4_ and PVP aqueous solution using MW irradiation (see Supporting Information File 1). The morphology and size of the Pd NPs was determined by TEM micrographs. Unfortunately, these MW-synthesized NPs proved to be inactive as catalysts in the Mizoroki–Heck coupling reaction between **1a** and **2a** in aqueous medium.

Thus, the PVP-Pd NPs electrochemically preformed showed a high catalytic activity in the Mizoroki–Heck reaction, employing nontoxic solvents and without the need to previously activation [19] or treatment of the catalyst. Furthermore, this nanocatalyst exhibited an outstanding activity for aryl bromides. We previously reported that [47] in the coupling of **1a** with **2a** under the same reaction conditions with conventional heating and in the presence of 0.2 mol % of PVP-Pd NPs only 39% yield of product **3** was achieved after 24 hours. Thus, synergism between MW and Pd NPs can lead to really efficient and sustainable catalytic systems. It is noteworthy that besides the high conversion, the TOF (h^−1^) values found for Mizoroki–Heck coupling reactions to obtain stilbene **3** (TOF ≈11000 h^−1^, entry 2, Table 1) were relative high for aryl bromides [50–51], even by comparison of the present protocol with other catalytic systems with Pd NPs under MW heating [52].

In order to extend the catalytic applications of this PVP-Pd NPs in the Mizoroki–Heck reaction, a substrate screening was carried out to determine the reaction scope and particularly the synthetic applications of this nanocatalyst. Thus, a variety of aryl halides **1a–h** and olefins **2a–c** were screened, employing the best reaction conditions above obtained (0.5 mmol of K_2_CO_3_, H_2_O/EtOH, at 130 ºC, Table 2).

**Table 2:** Mizoroki–Heck reaction of aryl halides with olefins catalyzed by PVP-Pd NPs.^a^

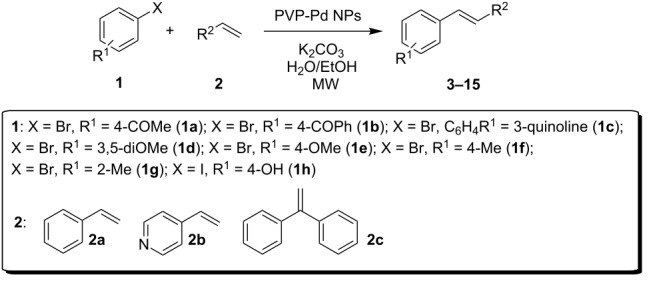						

Entry	**1**	**2**	% Pd *t* (min) *T* (°C)	Product	Yield (%)^b^	TOF (h^−1^) (TON)^c^

1	**1a**	**2a**	0.1 10 130	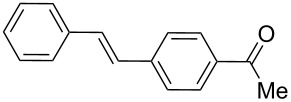 **3**	100	6000 (1000)
2	**1b**	**2a**	0.1 10 130	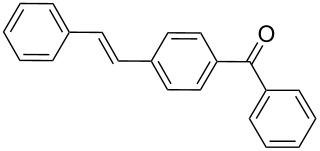 **4**	85	5100 (850)
3	**1c**	**2a**	0.2 15 130	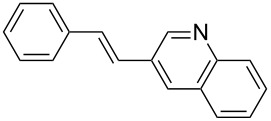 **5**	96	1920 (480)
4	**1d**	**2a**	0.2 10 130	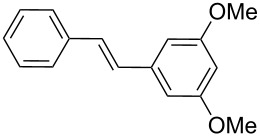 **6**	98	1960 (490)
5	**1e**	**2a**	0.2 15 150	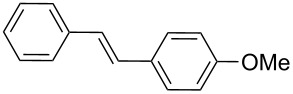 **7**	81	1620 (405)
6	**1f**	**2a**	0.2 15 150	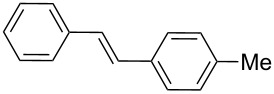 **8**	81	1620 (405)
7	**1g**	**2a**	0.2 15 150	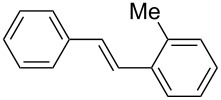 **9**	60	1200 (300)
8	**1h**	**2a**	0.05 5 130	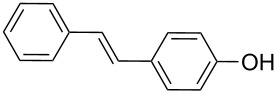 **10**	83	19920 (1660)
9	**1a**	**2b**	0.1 15 130	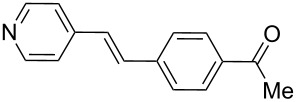 **11**	89	3560 (890)
10	**1b**	**2b**	0.1 15 150	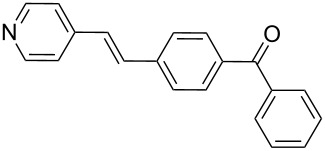 **12**	87	3480 (870)
11	**1c**	**2b**	0.2 15 150	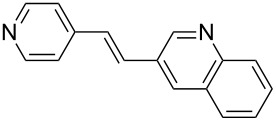 **13**	96	1920 (480)
12	**1d**	**2b**	0.2 15 150	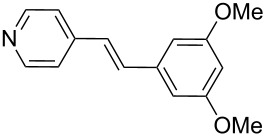 **14**	96	1920 (480)
13	**1a**	**2c**	0.2 15 130	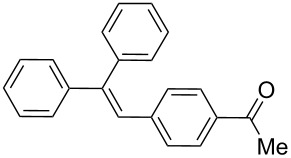 **15**	40	800 (200)

^a^Reaction conditions: 0.25 mmol of aryl halide, 0.30 mmol of olefin, 0.5 mmol of K_2_CO_3_, 2 mL of the mixed H_2_O/EtOH 3:1, MW (dynamic heating method) in a sealed vessel. ^b^GC yields. The yields reported represent at least the average of two reactions. ^c^TOF (turnover frequency, mol substrate converted per mol of Pd per hour). TON (turnover number, mol substrate converted per mol of Pd).

In all cases, clean and high selective reactions to obtain the *trans*-products were achieved.

By using styrene (**2a**) and aryl bromides with electron-withdrawing groups as coupling partners, such as 4-bromoacetophenone (**1a**) and 4-bromobenzophenone (**1b**), the reactions were carried out in short times, employing low catalyst loading, obtaining the (*E*)-stilbenes **3** and **4** with excellent yields and selectivity (entries 1 and 2, Table 2).

Despite that the synthesis of substituted heteroaryl derivatives is of great importance, only few examples employing heteroaryl halides in the Mizoroki–Heck reaction were reported [53]. This might be attributed to electronic aspects or poison by the heteroatom to the active Pd species [54]. However, when the coupling reaction with 3-bromoquinoline (**1c**) was carried out with PVP-Pd NPs as catalyst, the desired product **5** was obtained in excellent yield (entry 3, Table 2). The reaction conditions developed to obtain stilbene **5** are better than the previously reported ones (higher yields, short reaction time and more benign solvent) [55].

In addition, aryl bromides with electron-donating substituents were evaluated in the Mizoroki–Heck reaction catalyzed by PVP-Pd NPs (entries 4–7, Table 2). As general trend, with styrene (**2a**) as coupling partner, aryl bromides with an electron-withdrawing group or containing a nitrogen heterocycle (entries 1–3, Table 2) react better than aryl bromides with electron-donating groups (entries 4–7, Table 2). In the latter cases, higher temperatures, or a slight increase in reaction time and catalyst loading were needed to obtain the desired (*E*)-stilbenes **6–9** in good yields. When an *ortho*-substituted arene, **1g**, was used, product **9** was obtained in only moderate yield compared with *para*-substituted stilbene **8** synthesized under the same conditions (entries 6 and 7, Table 2). This behavior can be explained by the steric hindrance caused by the methylene group in *ortho*-position. The coupling of styrene with a substrate bearing an acidic proton, 4-iodophenol (**1h**), was found to proceed efficiently, obtaining a high TOF of 19920 h^−1^ (entry 8, Table 2). Unfortunately, it was not possible to successfully accomplish the Mizoroki–Heck coupling with aryl chlorides under many tested reaction conditions.

Additionally, the heterocyclic alkene 4-vinylpyridine (**2b**) was used as a coupling partner in the presence of aryl bromides **1a–d**. Even though olefin **2b** was shown to be slightly less reactive than styrene (**2a**), the heteroaromatic coupling products were afforded in excellent yields (entries 9–12, Table 2). By this methodology, new heteroaromatic olefins **11–13** were synthesized. The reaction with the symmetric *gem*-substituted olefin 1,1-diphenylethylene (**2c**) and 4-bromoacetophenone (**1a**) gave the trisubstituted olefin **15** in moderate yield (40%, entry 13, Table 2) [56].

On the other hand, and in order to further explore the scope of the PVP-Pd NPs catalyst, the synthesis of stilbene derivatives by Pd-tandem reactions was carried out. Some synthetic methods employing consecutive Pd Hiyama–Heck coupling reactions have been reported to obtain a variety of stilbene derivatives [55,57–58]. In our case, consecutive Pd-catalyzed Stille–Heck coupling reactions were explored in the presence of PVP-Pd NPs under MW irradiation to obtain the stilbenes. The results are summarized in Table 3.

**Table 3:** Stille–Heck tandem reaction of aryl halides with tributyl(vinyl)stannane.^a^

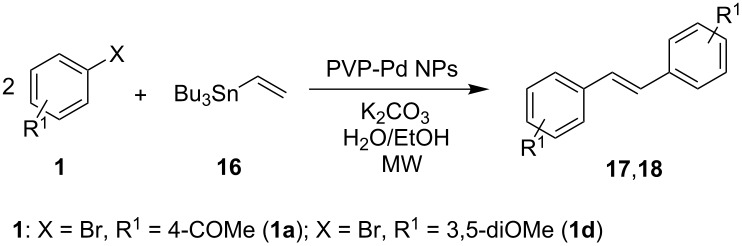					

Entry	1	% Pd	Product	Yield (%)^b^	TOF (h^−1^) (TON)^c^

1	**1a**	0.1	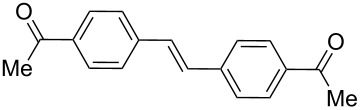 **17**	83	3320 (1660)
2	**1d**	0.2	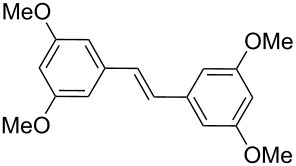 **18**	80	1600 (800)

^a^Reaction conditions: 0.5 mmol of aryl halide, 0.25 mmol of tributyl(vinyl)stannane (**16**), 1 mmol of K_2_CO_3_, 4 mL of H_2_O/EtOH 3:1, 130 °C MW (dynamic heating method) in a sealed vessel during 30 minutes. ^b^GC yields. The yields reported represent at least the average of two reactions. ^c^TON (turnover number, mol substrate converted per mol of Pd). TOF (turnover frequency, mol substrate converted per mol of Pd per hour).

4-Bromoacetophenone (**1a**) and 1-bromo-3,5-dimethoxybenzene (**1d**) were chosen to react with tributyl(vinyl)stannane (**16**). In both cases, the desired stilbenes **17** and **18** were obtained in good yield and in short reaction time under MW irradiation, besides the different electronic characteristic of substrates **1a** and **1d** (entries 1 and 2, Table 3). Thus, it was found that the PVP-Pd NPs were effective to catalyze two different reactions in a consecutive way. High yields and selectivity of the coupling products were obtained. These examples provide the first report about consecutive Stille–Heck reactions in aqueous media under MW irradiation.

**Scheme 1:**
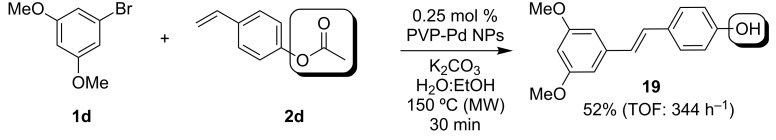
Synthesis of (*E*)-pterostilbene (**19**) catalyzed by PVP-Pd NPs.

Once the scope of the reaction was established, we were interested in investigating the synthetic applicability of this catalyst in a natural product synthesis. For this purpose, the reaction between aryl bromide **1d** and 4-acetoxystyrene (**2d**) was studied to obtain (*E*)-pterostilbene (**19**, Scheme 1).

Several derivatives of polyphenolic stilbenes as (*E*)-pterostilbene are natural products, which present an interesting biological activity [59]. Under the above mentioned reaction conditions (*E*)-pterostilbene (**19**) was obtained in moderate yield, but with a high TOF value considering the use of deactivated aryl bromide **1d**. Additionally, cleavage of the acetyl group in reagent **2d** occurred in the same reaction media without any additional steps. This issue should not be a trouble for the reaction in view of the coupling takes place properly in the presence of hydroxy groups. The synthetic procedures proposed for this type of stilbenes involves several steps, in which a Wittig reaction followed by different metal-catalyzed reactions and harsh conditions are usually required [60–62]. In our case, the desired product was obtained under environmentally friendly reaction conditions and in a short reaction time.

Thus, the PVP-Pd NPs proved to be an effective catalyst to perform the coupling reaction between a wide range of aryl bromides and different alkenes. Besides, with demanding substrates like heterocycles, *ortho*-substituted and non-activated aryl halides, very good results were achieved.

**Figure 1:**
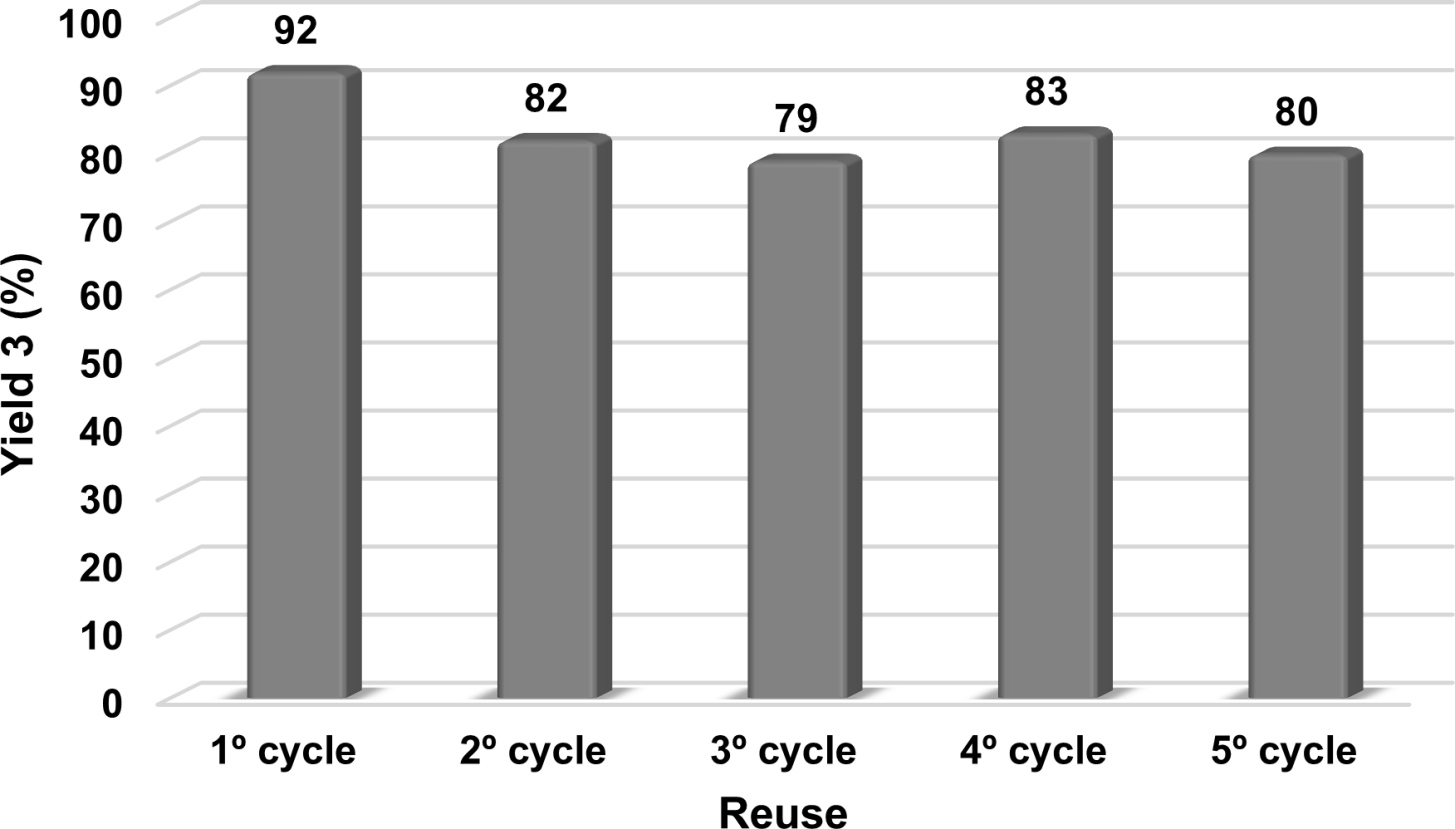
Reuse experiments of PdNPs in the coupling reaction between 4-bromoacetophenone (**1a**) and styrene (**2a**). Time: 1º cycle: 10 min; 2º cycle: 20 min; 3º cycle: 30 min, 4º cycle: 30 min, 5º cycle: 30 min.

Finally, the reusability of PVP-Pd NPs was evaluated in the reaction between 4-bromoacetophenone (**1a**) and styrene (**2a**). In our previous reports [46–48], it was exposed that a PVP-Pd NPs aqueous dispersion exhibited a high stability, and for that reason, the Pd NPs cannot be separated by a physical technique. Any attempt to separate the nanocatalyst from reaction media by centrifugation, precipitation or organic extraction eventually lead to a dramatic drop of the catalytic activity. As a result of this, and in order to perform the reuse experiment, repeated runs on the same batch were performed; reusing the reaction mixture with the PVP-Pd nanocatalyst. By this procedure in both, Suzuki–Miyaura coupling reaction [46] and nitroaromatic hydrogenation [48], the PVP-Pd NPs catalytic system exhibited high catalytic activity after five cycles. In the Heck–Mizoroki coupling reaction under MW irradiation, once the reaction was completed, a new batch of reagents was added to the reaction tube and it was once more heated by MW irradiation for the needed time. After that, a GC analysis of the reaction mixture was performed and product **3** was quantified (Figure 1).

Although the activity of PVP-Pd NPs slightly decreased in the second cycle, stilbene **3** was still obtained in good yields after five cycles of the Heck–Mizoroki coupling reaction. The initial drop could be explained by taking into account that the product and substrates are not soluble in the aqueous media causing a non-homogeneous heating and stirring of the system [63].

TEM micrograph of PVP-Pd NPs before and after one catalytic cycle was performed. The physical separation of NPs was carried out by centrifugation as previously reported [48]. The comparison of both TEM micrographs disclosed that the original PVP-Pd NPs [(9 ± 2) nm] clump to larger aggregates after the first catalytic cycle, providing NPs with a mean diameter of (20 ± 10) nm with high dispersion in size (Figures S1 and S2, Supporting Information File 1). However, these bigger NPs were still active as catalysts, since with this larger NPs several catalytic cycles were performed (Figure 1). The morphology of the NPs like a “blackberry” appeared not to be modified, and probably this structure is responsible for the activity since it leaves enough surface available to perform the catalytic reaction.

## Conclusion

In summary, the PVP-Pd NPs exhibit an outstanding catalytic activity in the Mizoroki–Heck reaction under environmentally friendly reaction conditions employing aqueous solvent and MW irradiation. Under the optimized conditions (0.05–0.25 mol % Pd NPs, 2 equiv of K_2_CO_3_, 2 mL of H_2_O/EtOH 3:1, at 130–150 ºC), the corresponding stilbenes and novel heterostilbenes could be obtained in good to excellent yields (40–100%) from aryl/heteroaryl bromides and different olefins. The stilbenes were achieved with high selectivity for *trans*-products in short reaction time leading to high reaction rates (TOFs of the order of 10^3^) for bromide derivatives. When less reactive electrophiles were used, the catalytic performance depended on the Pd NPs loading. Fundamental properties including high robustness, efficiency and TOFs, mild reaction conditions, utilization of aqueous media as a green solvent and simple product work-up make this catalytic system favorable from the environmental and economic point of view. Reusability experiments of the reaction mixture showed that PVP-Pd NPs maintain their catalytic activity after an initial little drop for at least five cycles. Moreover, the PVP-Pd NPs catalyst system was applied to a tandem Stille–Heck reaction.

## Experimental

### Materials and methods

4-Bromoacetophenone (**1a**), 4-bromobenzophenone (**1b**), 3-bromoquinoline (**1c**) 1-bromo-3,5-dimethoxybenzene (**1d**), 4-bromoanisole (**1e**), 4-bromotoluene (**1f**), 2-bromotoluene (**1g**), 4-iodophenol (**1h**), styrene (**2a**), 4-vinylpyridine (**2b**), 1,1-diphenylethylene (**2c**), 4-acetoxystyrene (**2d**), tributyl(vinyl)stannane (**16**). K_3_PO_4_, Na_2_CO_3_, K_2_CO_3_, sodium acetate (AcONa), ethanol 98% (EtOH), isopropanol (iPrOH), ethylene glycol and anhydrous Na_2_SO_4_ were used without purification. Electrolytes for the electrochemical experiments contain KNO_3_, H_2_PdCl_4_ and PVP polymer [poly-(*N*-vinylpyrrolidone)] (*M*_W_ 10000 Da), HCl 35%.

All solvents were of analytical grade and distilled before use. All reactions were carried out under air atmosphere. Silica gel (0.063–0.200 mm) was used for column chromatography. An Autolab PGSTAT100 (ECO CHEMIE) potentiostat–galvanostat was used for both, the potentiodynamic experiments and the galvanostatic pulses. Gas chromatographic analysis were performed on a gas chromatograph with a flame ionization detector, and equipped with the following columns: HP-5 25 m × 0.20 mm × 0.25 μm column. ^1^H NMR and ^13^C NMR were conducted on a high resolution spectrometer Bruker Advance 400, in CDCl_3_ as solvent. Gas chromatographic/mass spectrometer analyses were carried out on a GC–MS QP 5050 spectrometer equipped with a VF-5 ms, 30 m × 0.25 mm × 0.25 μm column. Melting points were determined with an electrical instrument. MW-induced reactions were performed in a CEM Focused Microwave TM Synthesis System, Model Discover single mode instrument. Transmission electron microscopy was conducted in a JEM-Jeol 1120 operating at 80 kV, at the IFFIVE Research Institute, INTA, Córdoba, Argentina. In order to characterize NPs by TEM, samples were prepared through depositing a drop of colloidal PVP-Pd NPs solution on a formvar-carbon-coated cooper grid and dried at room temperature. The total content of Pd was determined by atomic absorption in a Perkin Elmer Analyst 600, using ET (electro thermal mode with graphite furnace) at the ISIDSA Institute, Universidad Nacional de Córdoba, Córdoba, Argentina. Aqueous solutions were prepared from analytical grade chemicals and Milli-Q Millipore water.

### Synthesis of Pd nanoparticles suspension by electrochemical reduction

The electrochemical synthesis of PVP-Pd NPs was carried out as previously reported [46]. The experiments were achieved in a glass electrochemical cell equipped with a Pt disc working electrode (geometric area = 0.0746 cm^2^), a very large area sheet of Pt (counter electrode) and a saturated calomel reference electrode (SCE). The Pd NPs dispersions were obtained through Pd(II) electroreduction in KNO_3_ (0.1 M) and H_2_PdCl_4_ (0.5 × 10^−3^ M) solutions (pH 3.0) by applying a current density pulse at the Pt electrode in the presence of PVP as the stabilizing agent under solution stirring. The galvanostatic synthesis of Pd NPs was performed by applying to the platinum electrode a current density pulse from 0 to a cathodic value of 150 mA/cm^2^, during 600 s. Vigorous stirring of the solution (1000 rpm) with a magnetic stirrer was kept during the galvanostatic electrolysis. After completion of the reaction the aqueous dispersion of Pd NPs was placed in a 25 mL volumetric flask to be used as catalyst solution for Mizoroki–Heck coupling reaction without further purification. Average dimensions and shapes of Pd NPs were determined by transmission electron microscope (TEM) images and the total content of Pd in the colloidal suspensions was determined by atomic absorption.

### Synthesis of Pd nanoparticle suspensions under MW irradiation

In a 10 mL MW tube equipped with a magnetic stirring bar, 0.125 mL of H_2_PdCl_4_ 2 mM (2.5 × 10^−4^ mmol), 16 g/L of PVP 10000 Da (14.24 mg), 0.5 mL of ethanol, and 1.75 mL of bidistilled H_2_O were added. Then, the reaction tube was sealed with a rubber cap and heated at 130 °C for 10 minutes under MW irradiation (fixed temperature method) using air cooling. Average dimensions and shape of Pd NPs were determined by transmission electron microscope (TEM) images.

### Representative procedure for MW-assisted Mizoroki–Heck coupling reactions

The experimental procedure for MW-assisted Mizoroki–Heck coupling reactions was carried out analogously to the procedure previously reported [47]. Into a 10 mL MW tube equipped with a magnetic stirring bar, aryl halide **1a–h** (0.25 mmol), K_2_CO_3_ (0.5 mmol), olefin **2a–d** (0.3 mmol), ethanol (0.5 mL) and water (to obtain a total volume of 2 mL taking into account the volume of Pd NPs solution) were added. Finally, the required volume of the Pd NPs dispersion was added. Then, the reaction tube was sealed with a rubber cap and heated at 130–150 °C for 5–30 minutes under MW irradiation (fixed temperature method) using air cooling. After that, the reaction mixture was cooled to room temperature, extracted three times with ethyl acetate (15 mL each) and dried with anhydrous Na_2_SO_4_. The stilbene products **3–15** were purified by silica gel column chromatography. The products were characterized by ^1^H NMR, ^13^C NMR, and GC–MS. All spectroscopic data were in agreement with those previously reported for the following compounds: (*E*)-1-(4-styrylphenyl)ethanone (**3**) [64], (*E*)-phenyl(4-styrylphenyl)methanone (**4**) [64], (*E*)-3-styrylquinoline (**5**) [55], (*E*)-1,3-dimethoxy-5-styrylbenzene (**6**) [64], (*E*)-1-methoxy-4-styrylbenzene (**7**) [64], (*E*)-1-methyl-4-styrylbenzene (**8**) [65], (*E*)-1-methyl-2-styrylbenzene (**9**) [65], (*E*)-4-styrylphenol (**10**) [66], (*E*)-4-(3,5-dimethoxystyryl)pyridine (**14**) [67], 1-(4-(2,2-diphenylvinyl)phenyl)ethanone (**15**) [56], and (*E*)-4-(3,5-dimethoxystyryl)phenol or pterostilbene (**19**) [60].

### Representative procedure for the MW-assisted Stille–Heck coupling reactions

Analogously to the procedure described in [47], into a 10 mL MW tube equipped with a magnetic stirring bar, aryl bromide **1a**, **1d** (0.5 mmol), K_2_CO_3_ (1 mmol), tributyl(vinyl)stannane (**16**, 0.25 mmol), ethanol (1 mL) and water (to obtain a total volume of 4 mL taking into account the volume of Pd NPs solution) were added. Finally, the required volume of the Pd NPs dispersion was added. Then, the reaction tube was sealed with a rubber cap and heated at 130 °C for 30 minutes under MW irradiation (fixed temperature method) using air cooling. After that, the reaction was cooled to room temperature, extracted three times with ethyl acetate (15 mL each) and dried with anhydrous Na_2_SO_4_. The stilbene products **17** and **18** were purified by silica gel column chromatography. The products were characterized by ^1^H NMR, ^13^C NMR, and GC–MS. All the spectroscopic data were in agreement with those previously reported for the following compounds: (*E*)-1,1'-(ethene-1,2-diylbis(4,1-phenylene))diethanone (**17**) [68] and (*E*)-1,2-bis(3,5-dimethoxyphenyl)ethane (**18**) [69].

### Catalyst reusability experiments in the MW-assisted Mizoroki–Heck coupling reaction

The reusability of PVP-Pd NPs was evaluated in the Mizoroki–Heck coupling reaction between 4-bromoacetophenone (**1a**) and styrene (**2a**). Initially, the first reaction cycle was carried out following the procedure above described, employing 0.1 mol % of Pd NPs dispersion and heating by MW irradiation for 10 minutes. After that, the same mixture was reused by addition of fresh amounts of reactants: 4-bromoacetophenone (0.125 mmol), K_2_CO_3_ (0.25 mmol), styrene (0.15 mmol), ethanol (0.5 mL) and water (0.35 mL), and heated again for the needed time. This procedure was repeated until the desired cycle was reached. In the final cycle, the reaction was processed as previously described.

## Supporting Information

File 1 TEM images of Pd nanoparticles, characterization data, and NMR spectra.
