# Supplementary material for: An effective Pd nanocatalyst in aqueous media: stilbene synthesis by Mizoroki–Heck coupling reaction under microwave irradiation
Source: Beilstein J Org Chem. 2017 Aug 18;13:1717–27. doi: 10.3762/bjoc.13.166 (PMC5564268; doi:10.3762/bjoc.13.166)
Supplement: File 1 — TEM images of Pd nanoparticles, characterization data, and NMR spectra. [file Beilstein_J_Org_Chem-13-1717-s001.pdf]

Supporting Information

for

An effective Pd nanocatalyst in aqueous media: stilbene synthesis by Mizoroki–Heck coupling reaction under microwave irradiation

Carolina S. García, Paula M. Uberman and Sandra E. Martín*

Address: INFIQC-CONICET- Universidad Nacional de Córdoba, Departamento de Química Orgánica, Facultad de Ciencias Químicas. Haya de la Torre y Medina Allende, Ciudad Universitaria, X5000HUA, Córdoba, Argentina

*Corresponding author

Email: Sandra Elizabeth Martín - martins@fcq.un.edu.ar

TEM images of Pd nanoparticles, characterization data, and NMR spectra

Contents

Experimental Section

1. TEM images of Pd nanoparticles S2

2. Characterization data S2

3. NMR Spectroscopy S10

4. References S78

Experimental methods

1 TEM images *of Pd nanoparticles*

1.1- TEM images of Pd nanoparticles synthesized by electrochemical reduction

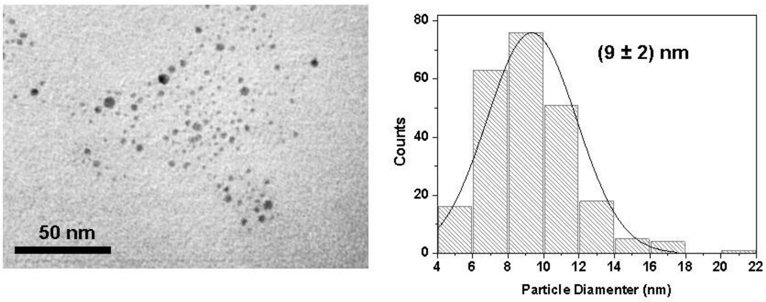

Figure S1: TEM image of PdNPs before catalysis.

1.2- TEM images of Pd nanoparticles recovery after catalysis

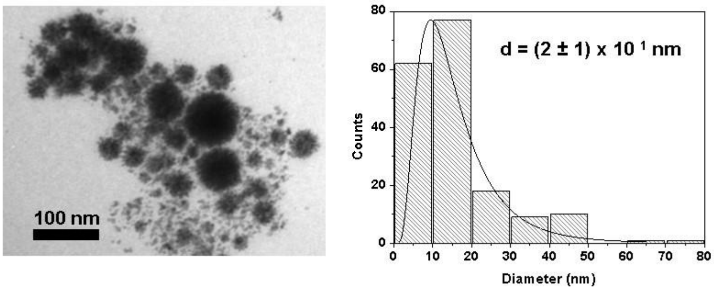

Figure S2: TEM image of PdNPs after one catalytic cycle.

1.3- TEM images of Pd nanoparticles synthesized by microwave irradiation

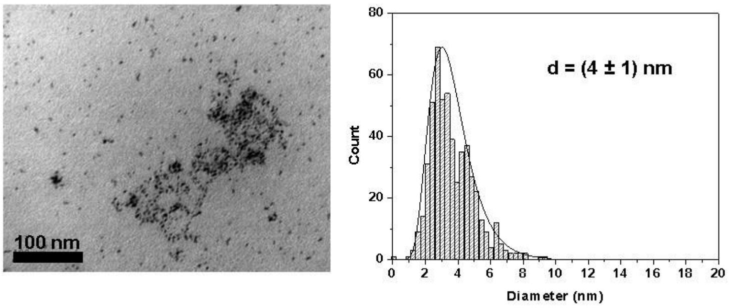

Figure S3: TEM images of the PdNPs obtained under microwave irradiation at 130 °C during 10 minutes.

2. Characterization data

The stilbene products were characterized by ^{1}H NMR, ^{13}C NMR, and GC–MS. All spectroscopic data were in agreement with those previously reported for the following compounds: (*E*)-1-(4-styrylphenyl)ethanone (**3**)[3], (*E*)-phenyl(4-styrylphenyl)methanone (**4**) [3], (*E*)-3-styrylquinoline (**5**)[4],

(*E*)-1,3-dimethoxy-5-styrylbenzene (**6**)[3], (*E*)-1-methoxy-4-styrylbenzene (**7**) [3], (*E*)-1-methyl-4-styrylbenzene (**8**)[5], (*E*)-1-methyl-2-styrylbenzene (**9**) [5], (*E*)-4-styrylphenol (**10**) [6], (*E*)-4-(3,5-dimethoxystyryl)pyridine (**14**)[7], 1-(4-(2,2-diphenylvinyl)phenyl)ethanone (**15**)[8], (*E*)-1,1'-(ethene-1,2-diylbis (4,1-phenylene))diethanone (**17**)[9], (E)-1,2-bis(3,5-dimethoxyphenyl)ethane (**18**)[10], and (*E*)-4-(3,5-dimethoxystyryl)phenol or pterostilbene (**19**)[11].

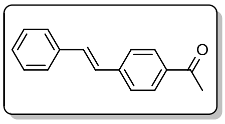

(*E*)-1-(4-Styrylphenyl)ethanone (3)

The product was separated by column chromatography on silica gel eluting with pentane/ethyl acetate gradient (100:0➔80:20) as a white solid.[1]

^{1}H NMR (400 MHz, $CDCl_3$) δ: 7.93 (d, *J* = 8.4 Hz, 2H), 7.57-7.51 (m, 4H), 7.38-7.35 (m, 2H), 7.31-7.29 (m, 1H), 7.20 (d, *J* = 16.4 Hz, 1H), 7.10 (d, *J* = 16.3 Hz, 1H), 2.58 (s, 3H). **^{13}C NMR (101 MHz, $CDCl_3$) δ:** 197.5 (C), 142.1 (C), 136.8 (C), 136.0 (C), 131.5(CH), 129.0 (CH), 128.9 (CH), 128.4 (CH), 127.5 (CH), 126.9 (CH), 126.6 (CH), 26.6 (CH_3). **^{1}H-^{1}H COSY NMR (400 MHz, $CDCl_3$) δ_H/δ_H:** 7.93/7.57-7.51, 7.57-7.51/7.38-7.35, 7.38-7.35/7.31-7.29, 7.20/7.10. ^{1}H-**^{13}C HSQC NMR (400 MHz, $CDCl_3$) δ_H /δ_C:** 7.93/129.0, 7.57-7.51/126.9, 7.57-7.51/126.6, 7.38-7.35/128.9, 7.31-7.29/128.4, 7.20/131.5, 7.10/127.5, 2.58/26.6. **^{1}H-^{13}C HMBC NMR (400 MHz, $CDCl_3$) δ_H /δ_C:** 7.93/128.9, 7.93/142.1, 7.93/197.5, 7.57-7.51/126.6, 7.57-7.51/128.4, 7.57-7.51/131.5, 7.57-7.51/136.0, 7.38-7.35/128.9, 7.38-7.35/136.9, 7.31-7.29/126.9, 7.20/126.9, 7.20/127.5, 7.20/136.8, 7.20/142.1, 7.10/126.6 7.10/131.5, 7.10/136.8, 7.10/142.1, 2.58/197.5, 2.58/136.0, 2.58/128.9. **GC-MS (70eV) m/z (%):** 43(20), 89 (15), 178 (62), 179 (22), 207 (100), [M^+] 222 (57).

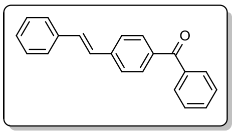

(*E*)-Phenyl(4-styrylphenyl)methanone (4)

The product was separated by column chromatography on silica gel eluting with pentane/ethyl ether gradient (100:0➔90:10) as a white solid.1

^{1}H NMR (400 MHz, $CDCl_3$) δ: 7.83-7.79 (m, 4H), 7.61-7.53 (m, 5H), 7.48 (t, *J* = 7.5 Hz, 2H), 7.38 (t, *J* = 7.5 Hz, 2H), 7.30 (d, *J* = 7.3 Hz, 1H), 7.24 (d, *J* = 16.3 Hz, 1H), 7.15 (d, *J* = 16.3 Hz, 1H). **^{13}C NMR (101 MHz, $CDCl_3$) δ:** 196.2 (C), 141.6 (C), 137.9 (C), 136.9 (C), 136.4 (C), 132.4 (CH), 131.5 (CH), 130.9 (CH), 130.1 (CH), 128.9 (CH), 128.4 (CH), 127.6 (CH), 126.9 (CH), 126.4 (CH). **^{1}H-^{1}H COSY NMR (400 MHz, $CDCl_3$) δ_H/δ_H:** 7.83-7.79/7.61-7.53, 7.83-7.79/7.48, 7.83-7.79/7.15 7.61-7.53/7.48, 7.61-7.53/7.38, 7.61-7.53/7.30, 7.61-7.53/7.24, 7.61-7.53/7.15, 7.38/7.30, 7.24/7.15. **^{1}H-^{13}C HSQC NMR (400 MHz, $CDCl_3$) δ_H/δ_C:** 7.83-7.79/130.9, 7.83-7.79/130.1, 7.61-7.53/132.4, 7.61-7.53/128.4, 7.61-7.53/126.9, 7.61-7.53/126.4, 7.48/127.6, 7.38/128.9, 7.29/128.4, 7.24/131.5, 7.15/127.6. **^{1}H-^{13}C HMBC NMR (400 MHz, $CDCl_3$) δ_H /δ_C:** 7.83-7.79/196.2, 7.83-7.79/141.6, 7.83-7.79/132.4, 7.83-7.79/130.9, 7.83-7.79/130.9, 7.83-7.79/130.1, 7.61-7.53/136.4, 7.61-7.53/131.5, 7.61-7.53/130.9, 7.61-7.53/130.1, 7.61-7.53/128.4, 7.61-7.53/127.6, 7.61-7.53/126.9, 7.61-7.53/126.4, 7.48/137.9, 7.48/128.4, 7.38/136.9, 7.38/128.9, 7.30/126.9, 7.24/141.6, 7.24/127.6, 7.24/126.9, 7.15/141.6, 7.15/136.9, 7.15/131.5, 7.15/126.4. **GC-MS (70eV) m/z (%):** 51 (16), 77 (50), 105 (62), 152 (17), 178 (75), 179 (45), 207 (100), 208 (15), [M^+] 284 (99), [M^++*1*] 285 (24).

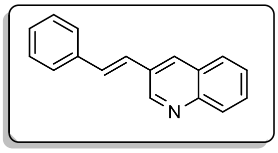

(*E*)-3-Styrylquinoline (5)

The product was separated by column chromatography on silica gel eluting with pentane/ethyl acetate (100:0➔85:15) as a white solid.[2]

^{1}H NMR (400 MHz, CDCl$_3$) δ: 9.12 (d, *J* = 2.2 Hz, 1H), 8.16 (d, *J* = 2.0 Hz, 1H), 8.09 (d, *J* = 8.4 Hz, 1H), 7.81 (d, *J* = 7.5 Hz, 1H), 7.69-7.65 (m, 1H), 7.59-7.51 (m, 3H), 7.41-7.38 (m, 2H), 7.34-7.30 (m, 2H), 7.22 (d, *J* = 16.4 Hz, 1H). **^{13}C NMR (101 MHz, CDCl$_3$) δ:** 149.6 (CH), 147.6 (C), 136.8 (C), 132.4 (CH), 131.0 (CH), 130.4 (C), 129.4 (CH), 129.3 (CH), 128.9 (CH), 128.4 (CH), 128.2 (C), 127.9 (CH), 127.1 (CH), 126.8 (CH), 125.3 (CH). **^{1}H-^{1}H COSY NMR (400 MHz, CDCl$_3$) δ_H/δ_H:** 9.12/8.16, 9.12/7.22, 8.16/8.09, 7.81/7.96-7.65, 8.09/7.59-7.51, 7.69-7.65/7.59-7.51, 7.59-7.51/7.41-7.38, 7.59-7.51/7.34-7.30, 7.41-7.38/7.34-7.30, 7.34-7.30/7.22. **^{1}H-^{13}C HSQC NMR (400 MHz, CDCl$_3$) δ_H/δ_C:** 9.12/149.6, 8.16/132.4, 8.09/129.4, 7.81/129.4, 7.59-7.51/127.1, 7.59-7.51/126.8, 7.41-7.38/128.9, 7.34-7.30/128.4, 7.34-7.30/125.3, 7.22/131.0. **^{1}H-^{13}C HMBC NMR (400 MHz, CDCl$_3$) δ_H/δ_C:** 9.12/147.6, 9.12/132.4, 9.12/130.4, 8.16/149.6, 8.16/147.6, 8.16/127.9, 8.16/125.3, 8.09/128.2, 8.09/127.1, 7.81/147.6, 7.81/132.4 7.81/129.3, 7.69-7.65/147.6, 7.69-7.65/127.9, 7.59-7.51/131.0, 7.59-7.51/129.4, 7.59-7.51/129.3, 7.59-7.51/128.4, 7.59-7.51/128.2, 7.59-7.51/126.8, 7.41-7.38/136.8, 7.41-7.38/128.9, 7.34-7.30/136.8, 7.34-7.30/130.4, 7.34-7.30/126.8, 7.34-7.30/125.3, 7.22/149.7, 7.22/136.8, 7.22/132.4, 7.22/131.0, 7.22/130.4. **GC-MS (70eV) m/z (%):** 101 (16), 115 (18), 202 (17), 230 (100), [M^+] 231 (71).

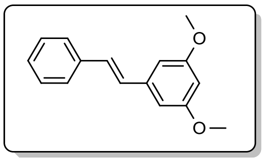

(*E*)-1,3-Dimethoxy-5-styrylbenzene (6)

The product was separated by column chromatography on silica gel eluting with pentane/diethyl ether (100:00➔90:10) as a white solid.1

^{1}H NMR (400 MHz, CDCl$_3$) δ: 7.49 (d, *J* = 7.4 Hz, 2H), 7.36-7.32 (m, 2H), 7.26-7.23 (m, 1H), 7.08 (d, *J* = 16.3 Hz, 1H), 7.02 (d, *J* = 16.3 Hz, 1H), 6.67 (d, *J* = 2.2 Hz, 2H), 6.39 (t, *J* = 2.2 Hz, 1H), 3.81 (s, 6H). **^{13}C NMR (101 MHz, CDCl$_3$) δ:** 161.1 (C), 139.5 (C), 137.3 (C), 129.3 (CH), 128.8 (CH), 127.8 (CH), 126.7 (CH), 104.7 (CH), 100.1 (CH), 55.5 (CH_3). **^{1}H-^{1}H COSY NMR (400 MHz, CDCl$_3$) δ_H/δ_H:** 7.49/7.36-7.32, 7.49/7.26-7.23, 7.08/7.02, 6.67/6.39. **^{1}H-^{13}C HSQC NMR (400 MHz, CDCl$_3$) δ_H/δ_C:** 7.49/126.7, 7.36-7.32/128.8, 7.26-7.23/127.8, 7.08/129.3, 7.02/128.8, 6.67/104.7, 6.39/100.1, 3.81/55.5. **^{1}H-^{13}C HMBC NMR (400 MHz, CDCl$_3$) δ_H/δ_C:** 7.49/129.3, 7.49/127.8, 7.49/126.7, 7.36-7.32/128.8, 7.36-7.32/137.3, 7.26-7.23/126.7, 7.08/139.5, 7.08/137.3, 7.08/128.8, 7.08/126.7, 7.02/139.5, 7.02/137.3, 7.02/129.3, 7.02/104.7, 6.67/161.1, 6.67/128.8, 6.67/104.1, 6.67/100.1, 3.81/161.1. **GC-MS (70eV) m/z (%):** 152 (17), 165 (39), 209 (21), 239 (24), [M^+] 240 (100), [M^++*1*] 241 (21).

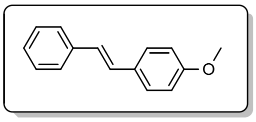

(*E*)-1-Methoxy-4-styrylbenzene (7)

The product was separated by column chromatography on silica gel eluting with pentane/diethyl ether gradient (100:0➔90:10) as a white solid.1

^{1}H NMR (400 MHz, CDCl$_3$) δ: 7.50-7.44 (m, 4H), 7.34 (t, *J* = 7.6 Hz, 2H), 7.25 – 7.21 (m, 1H), 7.07 (d, *J* = 16.3 Hz 1H), 6.97 (d, *J* = 16.3, 1H), 6.90 (d, *J* = 8.7 Hz, 2H) 3.83 (s, 1H). **^{13}C NMR (101**

MHz, $CDCl_3$) δ: 159.4 (C), 137.8 (C), 130.3 (C), 128.8 (CH), 128.3 (CH), 127.9 (CH), 127.4 (CH), 126.7 (CH), 126.4 (CH), 114.3 (CH), 55.5 (CH_3). **^{1}H-^{1}H COSY NMR (400 MHz, $CDCl_3$) δ_H/δ_H:** 7.50-7.44/7.34, 7.50-7.44/6.90, 7.34/7.25 – 7.21, 7.07/6.97. **^{1}H-^{13}C HSQC NMR (400 MHz, $CDCl_3$) δ_H/δ_C:** 7.50-7.44/127.9, 7.50-7.44/126.4, 7.34/128.8, 7.25 – 7.21/127.4, 7.07/128.3, 6.97/126.7, 6.90/114.3, 3.83/55.5. **^{1}H-^{13}C HMBC NMR (400 MHz, $CDCl_3$) δ_H/δ_C:** 7.50-7.44/159.4, 7.50-7.44/127.9, 7.50-7.44/127.4, 7.50-7.44/127.3, 7.50-7.44/126.4, 7.34/137.8, 7.34/128.8, 7.25-7.21/126.4, 7.07/137.8, 7.07/127.9, 7.07/126.7, 6.97/130.2, 6.97/128.3, 6.97/126.4, 6.90/159.4, 6.90/130.3, 3.83/159.4. **GC-MS (70eV) m/z (%):** 152 (21), 165 (32), 167 (34), 179 (15), 195 (18), [M^+] 210 (100), [M^++*1*] 211 (15).

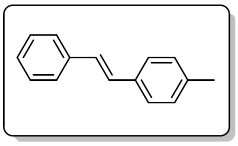

***(E)*-1-Methyl-4-styrylbenzene (8)**

The product was separated by column chromatography on silica gel eluting with pentane/dichloromethane (100:0→98:2) as a white solid.[3]

^{1}H NMR (400 MHz, $CDCl_3$) δ: 7.51-7.49 (m, 2H), 7.41 (d, *J* = 8.1 Hz, 2H), 7.36-7.32 (m, 2H), 7.26-7.21 (m, 1H), 7.16 (d, *J* = 7.9 Hz, 2H), 7.09 (d, *J* = 16.4 Hz, 1H), 7.05 (d, *J* = 16.4 Hz, 1H), 2.35 (s, 3H). **^{13}C NMR (101 MHz, $CDCl_3$) δ:** 137.7 (C), 134.7(C), 129.5 (CH), 128.8 (CH), 128.7 (CH), 127.8 (CH), 127.5 (CH), 126.6 (CH), 126.5 (CH), 21.4 (CH_3). **^{1}H-^{1}H COSY NMR (400 MHz, $CDCl_3$) δ_H/δ_H:** 7.51-7.49/7.36-7.32, 7.41/7.16, 7.17/2.35. **^{1}H-^{13}C HSQC NMR (400 MHz, $CDCl_3$) δ_H/δ_C:** 7.51-7.49/126.5, 7.41/126.6, 7.36-7.32/128.7, 7.26-7.21/127.5, 7.16/129.5, 7.09/127.8, 7.05/128.8, 2.35/21.4. **^{1}H-^{13}C HMBC NMR (400 MHz, $CDCl_3$) δ_H/δ_C:** 7.51-7.49/127.8, 7.51-7.49/127.5, 7.51-7.49/126.6, 7.41/137.7, 7.41/128.8, 7.41/128.7, 7.41/126.5, 7.36-7.32/137.7, 7.36-7.32/128.8, 7.26-7.21/126.6, 7.16/134.7, 7.16/129.5, 7.16/21.4, 7.09/126.6, 7.09/134.7, 7.09/137.7, 7.05/137.6, 7.05/134,7,7.05/126.5, 2.35/137.7, 2.35/129.5. **GC-MS (70eV) m/z (%):** 178 (79), 179 (100), 193 (16), [M^+] 194 (89), [M^++*1*] 195 (15).

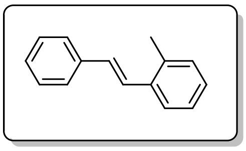

***(E)*-1-Methyl-2-styrylbenzene (9)**

The product was separated by column chromatography on silica gel eluting with pentane/diethyl ether (100:00→90:10) as a white solid (i.y: 28.9 mg; 53% without optimization).3

^{1}H NMR (400 MHz, $CDCl_3$) δ: 7.59 (d, *J* = 6.9 Hz, 1H), 7.53-7.51 (m, 2H), 7.37-7.31 (m, 3H), 7.28-7.24 (m, 1H), 7.22-7.16 (m, 3H), 6.99 (d, *J* = 16.1 Hz, 1H), 2.42 (s, 3H). **^{13}C NMR (101 MHz, $CDCl_3$) δ:** 137.8 (C), 136.5 (C), 135.9 (C), 130.5 (CH), 130.2 (CH), 128.8 (CH), 127.7 (CH), 127.6 (CH), 126.7 (CH), 126.3 (CH), 125.5 (CH), 20.0 (CH_3). **^{1}H-^{1}H COSY NMR (400 MHz, $CDCl_3$) δ_H/δ_H:** 7.59/7.28-7.24, 7.59/7.22-7.16, 7.53-7.51/7.37-7.31, 7.53-7.51/7.28-7.24, 7.53-7.51/6.99, 7.37-7.31/7.28-7.24, 7.37-7.31/6.99, 7.28-7.24/7.22-7.16. **^{1}H-^{13}C HSQC NMR (400 MHz, $CDCl_3$) δ_H/δ_C:** 7.59/125.5, 7.53-7.51/126.7, 7.37-7.31/128.8, 7.37-7.31/126.7, 7.28-7.24/127.7, 7.22-7.16/130.5, 7.22-7.16/127.6, 7.22-7.16/126.3, 6.99/130.2, 2.42/20.0. **^{1}H-^{13}C HMBC NMR (400 MHz, $CDCl_3$) δ_H/δ_C:** 7.59/135.9, 7.59/127.6, 7.53-7.51/130.2, 7.53-7.51/127.6, 7.53- 7.51/126.7, 7.37-7.31/137.8, 7.37-7.31/136.5, 7.37-7.31/135.9, 7.37-7.31/128.8, 7.37-7.31/125.5, 7.28-7.24/126.7, 6.99/136.5,

6.99/126.6, 2.42/136.5, 2.42/135.9, 2.42/130.5. **GC-MS (70eV) m/z (%):** 89 (16), 115 (32), 116 (21), 165 (15), 178 (75), 179 (100), 193 (17), [M^+] 194 (80).

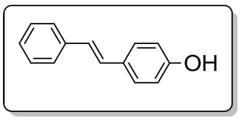

***(E)*-4-Styrylphenol (10)**

The product was separated by radial chromatography (centrifugal thin-layer chromatography) eluting with pentane/ethyl acetate (100:00➔85:15) as a white solid.[4]

^{1}H NMR (400 MHz, Acetone-d$_6$) δ: 8.48 (s, 1H), 7.56-7.54 (m, 2H), 7.46 (dt, *J* = 9.5 Hz, 1.76 Hz, 2H), 7.36-7.32 (m, 2H), 7.24-7.20 (m, 1H), 7.17 (d, *J* = 16.4 Hz, 1H), 7.05 (d, *J* = 16.4 Hz, 1H), 6.85 (dt, *J* = 9.5 Hz, 2 Hz, 2H). **^{13}C NMR (101 MHz, Acetone-d$_6$) δ:** 158.2 (C), 138.8 (C), 129.9 (C), 129.4 (CH), 129.3 (CH), 128.7 (CH), 127.8 (CH), 126.9 (CH), 126.4 (CH), 116.4 (CH). **^{1}H-^{1}H COSY NMR (400 MHz, Acetone- *d$_6$*) δ_H/δ_H:** 7.56-7.54/7.36-7.32, 7.56-7.54/7.24-7.20, 7.56-7.54/7.05, 7.46/7.17, 7.46/6.86, 7.36-7.32/7.24-7.20, 7.17/7.05, 7.17/6.86. **^{1}H-^{13}C HSQC NMR (400 MHz, Acetone- *d$_6$*) δ_H/δ_C:** 7.56-7.54/126.9, 7.46/128.7, 7.36-7.32/129.4, 7.24-7.20/127.8, 7.17/129.3, 7.05/126.4, 6.86/116.4. **^{1}H-^{13}C HMBC NMR (400 MHz, Acetone- *d$_6$*) δ_H/δ_C:** 7.56-7.54/129.4, 7.56-7.54/127.8, 7.56-7.54/126.9, 7.56-7.54/126.4, 7.46/158.2, 7.46/129.4, 7.46/128.3, 7.46/128.7, 7.46/116.4, 7.36-7.32/138.8, 7.36-7.32/129.4, 7.36-7.32/126.9, 7.24-7.20/126.9, 7.24-7.20/126.5, 7.17/138.8, 7.17/128.7, 7.17/126.4, 7.05/138.8, 7.05/129.9, 7.05/129.3, 7.05/126.8, 6.86/158.2, 6.86/129.8, 6.86/126.4, 6.86/116.4. **GC-MS (70eV) m/z (%):** 152 (18), 165 (26), 167 (24), 181 (20), 195 (43), [M^+] 196 (100).

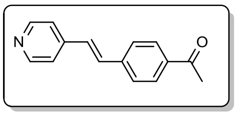

***(E)*-1-(4-(2-(Pyridin-4-yl)vinyl)phenyl)ethanone (11)**

The product was separated by column chromatography on silica gel eluting with pentane/ethyl acetate (100:00➔20:80) as a yellow solid (35.8 mg; 64%). Melting point: 104.2-105.2 °C.

^{1}H NMR (400 MHz, CDCl$_3$) δ: 8.60 (d, *J* = 5.7 Hz, 2H), 7.98-7.96 (m, 2H), 7.61 (d, *J* = 8.4 Hz, 2H), 7.37 (dd, *J* = 4.7, 1.4 Hz, 2H), 7.30 (d, *J* = 16.4 Hz, 1H), 7.11 (d, *J* = 16.4 Hz, 1H), 2.60 (s, 1H). **^{13}C NMR (101 MHz, CDCl$_3$) δ:** 197.4 (C), 150.4 (CH), 144.0 (C), 140.7 (C), 136.9 (C), 131.9 (CH), 129.0 (CH), 128.7 (CH), 127.1 (CH), 121.1 (CH), 26.7 (CH). **^{1}H-^{1}H COSY NMR (400 MHz, CDCl$_3$) δ_H/δ_H:** 8.60/7.37, 7.98-7.96/7.61, 7.30/7.11. **^{1}H-^{13}C HSQC NMR (400 MHz, CDCl$_3$) δ_H/δ_C:** 8.60/150.4, 7.98-7.96/129.0, 7.61/127.1, 7.37/121.1, 7.30/128.7, 7.14/131.9, 2.60/26.7. **^{1}H-^{13}C HMBC NMR (400 MHz, CDCl$_3$) δ_H/δ_C:** 8.60/150.4, 8.60/144.0, 8.60/121.1, 7.98-7.96/197.4, 7.98-7.96/140.7, 7.98-7.96/129.0, 7.61/136.9, 7.61/131.9, 7.61/127.1, 7.37/150.4, 7.37/128.7, 7.37/121.1, 7.30/144.0, 7.30/140.7, 7.30/131.9, 7.30/121.1, 7.11/144.0, 7.11/140.7, 7.11/131.9, 7.11/121.1, 2.60/197.4, 2.60/136.9, 2.60/129.0. **GC-MS (70eV) m/z (%):** 151 (16), 152 (45), 180 (21), 208 (100), [M^+] 223 (57). **HRMS (TOF; ESI$^+$):** calcd for $C_{15}H_{13}NNa^+$ $(M + Na)^+$: 246,0889; found: 246,0891.

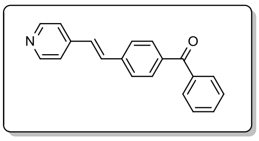

***(E)*-Phenyl(4-(2-(pyridin-4-yl)vinyl)phenyl)methanone (12)**

The product was separated by column chromatography on silica gel eluting with pentane/ethyl acetate (100:00➔30:70) as a white solid (59.4 mg; 84%). Melting point: 186.1-187.1°C.

^{1}H NMR (400 MHz, $CDCl_3$) δ: 8.61 (d, *J* = 4.2 Hz, 2H), 7.85-7.80 (m, 4H), 7.64-7.58 (m, 3H), 7.50 (t, *J* = 7.5 Hz, 2H), 7.40 (d, *J* = 5.0 Hz, 2H), 7.35 (d, *J* = 16.4 Hz, 1H), 7.14 (d, *J* = 16.3 Hz, 1H). **^{13}C NMR (101 MHz, $CDCl_3$) δ:** 196.0 (C), 150.5 (CH), 144.1 (C), 140.2 (C), 137.7 (C), 137.5 (C), 132.6 (CH), 132.1 (CH), 130.8 (CH), 130.1 (CH), 128.7 (CH), 128.5 (CH), 126.9 (CH), 121.1 (CH). **^{1}H-^{1}H COSY NMR (400 MHz, $CDCl_3$) δ_H/δ_H:** 8.61/7.40, 7.85-7.80/7.64-7.58, 7.64-7.58/7.50, 7.64-7.58/7.50, 7.64-7.58/7.35, 7.35/7.14. **^{1}H-^{13}C HSQC NMR (400 MHz, $CDCl_3$) δ_H/δ_C:** 8.61/150.5, 7.85-7.80/130.8, 7.85-7.80/130.1, 7.64-7.58/132.6, 7.64-7.58/126.9, 7.50/128.5, 7.40/121.1, 7.35/132.1, 7.14/128.7. **^{1}H-^{13}C HMBC NMR (400 MHz, $CDCl_3$) δ_H/δ_C:** 8.60/144.1, 8.60/121.1, 7.85-7.80/196.0, 7.85-7.80/140.2, 7.85-7.80/132.6, 7.85-7.80/130.8, 7.85-7.80/130.1, 7.85-7.80/126.9, 7.64-7.58/137.5, 7.64-7.58/132.1, 7.64-7.58/130.8, 7.64-7.58/130.1, 7.64-7.58/126.9, 7.50/137.7, 7.50/128.7, 7.50/121.1, 7.35/144.1, 7.35/128.7, 7.35/126.9, 7.14/140.2, 7.14/132.1, 7.14/121.1. **GC-MS (70eV) m/z (%):** 51 (16), 77 (59), 105 (69), 151 (19), 152 (51), 180 (18), 208 (94), [M^+] 285 (100), [M^++1] 286 (21). **HRMS (TOF; ESI$^+$):** calcd. for $C_{20}H_{15}NNaO^+$ (M + Na)$^+$:308,1046; found: 308,1058.

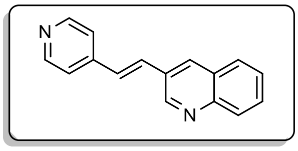

***(E)*-3-(2-(Pyridin-4-yl)vinyl)quinoline (13)**

The product was separated by column chromatography on silica gel eluting with ethyl acetate/ethanol (100:00➔90:10) as a white solid (38.4 mg; 66%). Melting point: 113-115°C.

^{1}H NMR (400 MHz, $CDCl_3$) δ: 9.13 (d, *J* = 2.2 Hz, 1H), 8.63 (dd, *J* = 4.6, 1.4 Hz, 2H), 8.22 (d, *J* = 2.0 Hz, 1H), 8.11 (d, *J* = 8.4 Hz, 1H), 7.84 (d, *J* = 8.1 Hz, 1H) (**e**), 7.72 (ddd, *J* = 8.4, 6.9, 1.4 Hz, 1H) (**f**), 7.57 (ddd, *J* = 8.1, 7.0, 1.1 Hz, 1H), 7.45-7.41 (m, 3H), 7.23 (d, *J* = 16.4 Hz, 1H). **^{13}C NMR (101 MHz, $CDCl_3$) δ:** 150.5 (CH), 149.3 (CH), 148.1 (C), 144.1 (C), 133.6 (CH), 130.0 (CH), 129.9 (CH), 129.5 (CH), 129.3 (C), 128.3 (CH), 128.1 (CH), 128.0 (C), 127.4 (CH), 121.0 (CH). **^{1}H-^{1}H COSY NMR (400 MHz, $CDCl_3$) δ_H/δ_H:** 9.13/8.22, 8.63/7.45-7.41, 8.11/7.72, 7.84/7.57, 7.72/7.57, 7.45-7.41/7.23. **^{1}H-^{13}C HSQC NMR (400 MHz, $CDCl_3$) δ_H/δ_C:** 9.13/149.3, 8.63/150.5, 8.22/133.6, 8.11/129.5, 7.84/128.1, 7.72/130.0, 7.57/127.4, 7.45-7.41/129.9, 7.45-7.41/121.0, 7.23/128.3. **^{1}H-^{13}C HMBC NMR (400 MHz, $CDCl_3$) δ_H/δ_C:** 9.13/148.1, 9.13/133.6, 9.13/129.3, 8.63/150.5, 8.63/144.1, 8.63/121.0, 8.22/149.3, 8.22/148.1 8.22/129.9, 8.22/128.1, 8.11/128.0, 8.11/127.4, 7.84/148.1, 7.84/133.6, 7.84/130.0, 7.72/148.1, 7.72/128.1, 7.57/130.0, 7.57/129.5, 7.57/128.1, 7.45-7.41/150.5, 7.45-7.41/149.3, 7.45-7.41/144.1, 7.45-7.41/133.6, 7.45-7.41/128.3, 7.45-7.41/121.0, 7.23/129.9, 7.23/129.3, 7.23/121.0. **GC-MS (70eV) m/z (%):** 51 (17), 102 (22), 204 (44), 231 (100), [M^+] 232 (99), [M^++*1*] 233 (18). **HRMS (TOF, ESI$^+$):** calcd for $C_{16}H_{12}N_2Na^+$ (M + Na)$^+$: 255.0893, found: 255.0888.

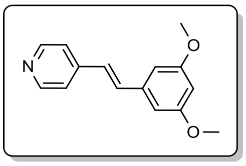

(*E*)-4-(3,5-Dimethoxystyryl)pyridine (14)

The product was separated by column chromatography on silica gel eluting with pentane/ethyl acetate (100:00➔40:60) as yellow oil.[5]

^{1}H NMR (400 MHz, CDCl$_3$) δ: 8.57 (dd. *J* = 4.7, 1.4 Hz, 2H), 7.34 (dd, *J* = 4.7, 1.4 Hz, 2H), 7.21 (d, *J* = 16.3 Hz, 1H), 6.96 (d, *J* = 16.2 Hz, 1H), 6.68 (d, *J* = 2.2 Hz, 2H), 6.45 (t, *J* = 2.2 Hz, 1H), 3.83 (s, 6H). **^{13}C NMR (101 MHz, CDCl$_3$) δ:** 161.2 (C), 150.3 (CH), 144.6 (C), 138.2 (C), 133.3 (CH), 126.6 (CH), 121.0 (CH), 105.2 (CH), 101.1 (CH), 55.5 (CH$_3$). **^{1}H-^{1}H COSY NMR (400 MHz, CDCl$_3$) δ$_H$/δ$_H$:** 8.57/7.34, 7.21/6.96, 6.68/6.45. **^{1}H-^{13}C HSQC NMR (400 MHz, CDCl$_3$) δ$_H$/δ$_C$:** 8.57/150.3, 7.34/121.0, 7.21/133.3, 6.96/126.6, 6.68/105.2, 6.45/101.1, 3.83/55.5. **^{1}H-^{13}C HMBC NMR (400 MHz, CDCl$_3$) δ$_H$/δ$_C$:** 8.57/150.3, 8.57/144.6, 8.57/121.0, 7.34/150.3, 7.34/126.6, 7.34/121.0, 7.21/144.6, 7.21/105.2, 6.96/138.2, 6.96/121.0, 6.68/161.2, 6.68/133.3, 6.68/195.2, 6.68/101.1, 6.45/161.2, 6.45/105.2, 3.83/161.2, **GC-MS (70eV) m/z (%):** 154 (15), 166 (15), 167 (23), 210 (19), 240 (52), [M^+] 241 (100).

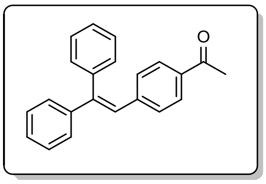

1-(4-(2,2-Diphenylvinyl)phenyl)ethanone (15)

The product was separated by column chromatography on silica gel eluting with pentane/ethyl ether (100:00➔90:10) as a white solid.[6]

^{1}H NMR (400 MHz, CDCl$_3$) δ: 7.72 (d, *J* = 8.5 Hz, 2H), 7.35-7.31 (m, 8H), 7.20-7.17 (m, 2H), 7.09 (d, *J* = 8.4 Hz, 2H), 6.99 (s, 1H), 2.53 (s, 3H). **^{13}C NMR (101 MHz, CDCl$_3$) δ:** 197.7 (C), 145.4 (C), 143.0 (C), 142.5 (C), 140.0 (C), 135.2 (C), 130.4 (CH), 129.7 (CH), 128.9 (CH), 128.4 (CH), 128.2 (CH), 128.1 (CH), 128.0 (CH), 127.9 (CH), 127.1 (CH), 26.7 (CH$_3$). **^{1}H-^{1}H COSY NMR (400 MHz, CDCl$_3$) δ$_H$/δ$_H$:** 7.72/7.09, 7.72/6.99, 7.35-7.31/7.20-7.17, 7.09/6.99. **^{1}H-^{13}C HSQC NMR (400 MHz, CDCl$_3$) δ$_H$/δ$_C$:** 7.72/128.2, 7.35-7.31/128.9, 7.35-7.31/128.4, 7.35-7.31/128.1, 7.35-7.31/128.0, 7.35-7.31/127.9, 7.20-7.17/130.4, 7.09/129.7, 6.99/127.1, 2.53/26.7. **^{1}H-^{13}C HMBC NMR (400 MHz, CDCl$_3$) δ$_H$/δ$_C$:** 7.72/197.7, 7.72/142.5, 7.72/128.2, 7.35-7.31/145.4, 7.35-7.31/143.0, 7.35-7.31/140.0, 7.35-7.31/128.9, 7.35-7.31/128.4, 7.35-7.31/128.0, 7.20-7.17/127.9, 7.09/135.2, 7.09/129.7, 7.09/127.1, 6.99/145.4, 6.99/143.0, 6.99/140.0, 6.99/129.7, 2.53/197.7, 2.53/135.3, 2.53/128.2. **GC-MS (70eV) m/z (%):** 43 (44), 113 (15), 126 (32), 178 (15), 215 (16), 226 (15), 239 (34), 240 (26), 252 (30), 253 (36), 25 (40), 283 (82), 284 (21), [M^+] 298 (100), [M^++*1*] 299 (22).

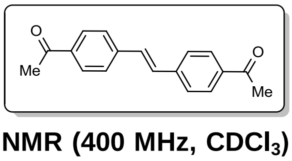

(*E*)-1,1'-(Ethene-1,2-diylbis(4,1-phenylene))diethanone (17)

The product was separated by column chromatography on silica gel eluting with pentane/ ethyl acetate (100:0➔50:50) as a yellow solid.[7] **^{1}H NMR (400 MHz, CDCl$_3$) δ**: 7.97 (d, J = 8.5 Hz, 4H), 7.62 (d, J = 8.4 Hz, 4H), 7.25 (s, 2H), 2.62 (s, 6H). **^{13}C NMR (101 MHz, CDCl$_3$) δ:** 197.5 (C), 141.4 (C), 136.6 (C), 130.3 (CH), 129.1 (CH), 127.0 (CH), 26.7 (CH3). **^{1}H-^{1}H COSY NMR (400 MHz; CDCl$_3$) δH/δH**: 7.97/7.62. **^{1}H-^{13}C HSQC NMR (400 MHz, CDCl$_3$) δH/δC**: 7.97/129.1, 7.62/127.0, 7.25/130.3, 2.62/26.7. **^{1}H-^{13}C HMBC NMR (400 MHz, CDCl$_3$) δH/δC**: 7.97/197.5, 7.97/141.4, 7.97/129.1, 7.62/136.6, 7.62/130.3, 7.62/127.0,

7.25/141.4, 7.25/127.0, 2.62/197.5, 2.62/136.6. **GC-MS (70eV) m/z (%):** 43 (100), 176 (15), 249 (95), [M^+] 264 (48).

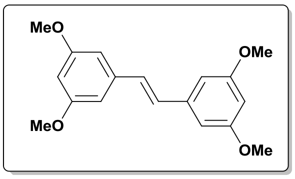

(*E*)-1,2-Bis(3,5-dimethoxyphenyl)ethane (18)

The product was separated by column chromatography on silica gel eluting with pentane/ diethyl ether (100:0➔90:10) as a white solid. [8]

^{1}H NMR (400 MHz; CDCl$_3$) δ: 7.01 (s, 2H), 6.66 (d, J = 2.2 Hz, 4H), 6.40 (t, J = 2.2 Hz, 2H), 3.83 (s, 12H). **^{13}C NMR (101 MHz; CDCl$_3$) δ**: 161.1 (C), 139.3 (C), 129.3 (CH), 104.8 (CH), 100.3 (CH), 55.5 (CH3). **^{1}H-^{1}H COSY NMR (400 MHz; CDCl$_3$) δH/δH**: 7.01/6.66, 6.66/6.40. **^{1}H-^{13}C HSQC NMR (400 MHz; CDCl$_3$) δH/δC**: 7.01/129.3, 6.66/104.8, 6.40/100.3, 3.83/55.5. **^{1}H-^{13}C HMBC NMR (400 MHz; CDCl$_3$) δH/δC**: 7.01/139.3, 7.01/129.3, 7.01/104.8, 6.66/161.1, 6.66/129.3, 6.66/104.8, 6.66/100.3, 6.40/161.1, 3.83/161.1. **GC-MS (70eV) m/z (%)**: 152 (15), 254 (15), 269 (49), 270 (22), 299 (16), [M^+] 300 (100), [M^++1] 301 (15).

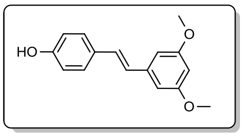

***(E)*-4-(3,5-Dimethoxystyryl)phenol (19)**

The product was separated by radial chromatography (centrifugal thin-layer chromatography) eluting with pentane/ethyl acetate (100:00➔70:30) as a white solid.[9]

^{1}H NMR (400 MHz, CDCl$_3$) δ: 7.39 (d, *J* = 8.6 Hz, 2H), 7.02 (d, *J* = 16.2 Hz, 1H), 6.88 (d, *J* = 16.2 Hz, 1H), 6.81 (d, *J* = 8.6 Hz, 2H), 6.65 (d, *J* = 2.2 Hz, 2H), 6.38 (t, *J* = 2.2 Hz, 1H), 5.28 (s, 1H), 3.82 (s, 6H). **^{13}C NMR (101 MHz, CDCl$_3$) δ:** 161.1 (C), 155.6 (C), 139.8 (C), 130.2 (C), 128.9 (CH), 128.1 (CH), 126.7 (CH), 115.8 (CH), 104.6 (CH), 99.8 (CH), 55.5 (CH$_3$). **^{1}H-^{1}H COSY NMR (400 MHz, CDCl$_3$) δ$_H$/δ$_H$:** 7.39/6.81, 7.02/6.88, 6.65/6.38. **^{1}H-^{13}C HSQC NMR (400 MHz, CDCl$_3$) δ$_H$/δ$_C$:** 7.39/128.1, 7.02/128.9, 6.88/126.7, 6.81/115.8, 6.65/104.6, 6.38/99.8, 3.82/55.5. **^{1}H-^{13}C HMBC NMR (400 MHz, CDCl$_3$) δ$_H$/δ$_C$:** 7.39/155.6, 7.39/128.9, 7.39/128.1, 7.02/139.8, 7.02/128.1, 7.02/126.7, 6.88/130.2, 6.88/128.9, 6.88/104.6, 6.81/155.6, 6.81/130.2, 6.81/115.8, 6.65/161.1, 6.65/126.7, 6.65/104.6, 6.65/99.8, 6.38/161.1, 6.38/104.6, 3.82/161.1. **GC-MS (70eV) m/z (%):** 76 (15), 181 (20), 182 (17), 207 (59), 225 (18), [M^+] 256 (100), [M^++1] 257 (34).

3. NMR Spectroscopy

^{1}H NMR (400 MHz, $CDCl_3$) (*E*)-1-(4-styrylphenyl)ethanone (3)

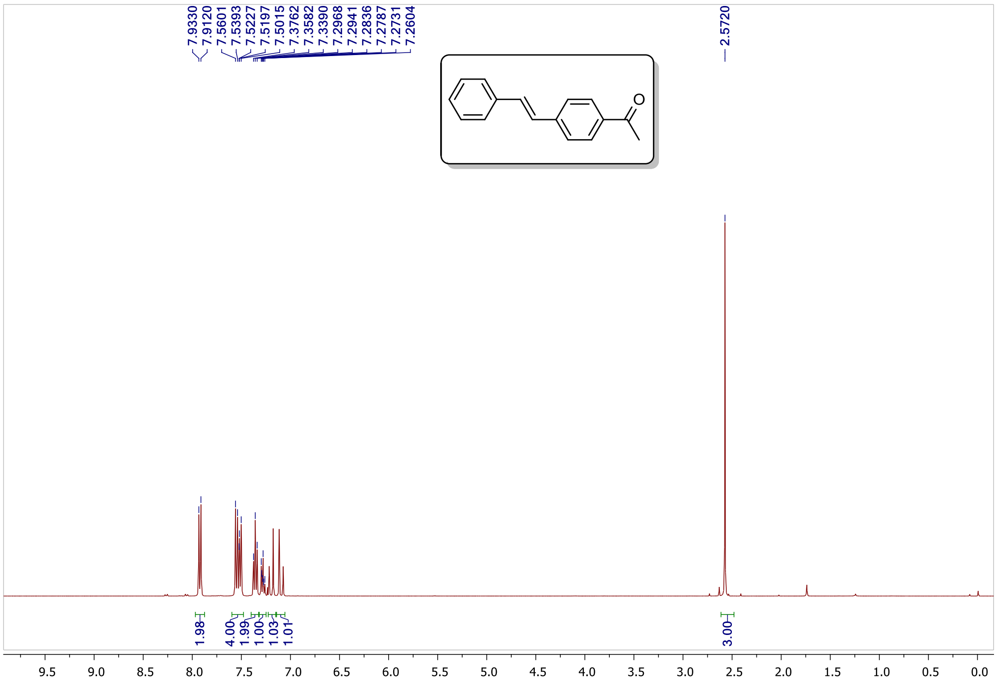

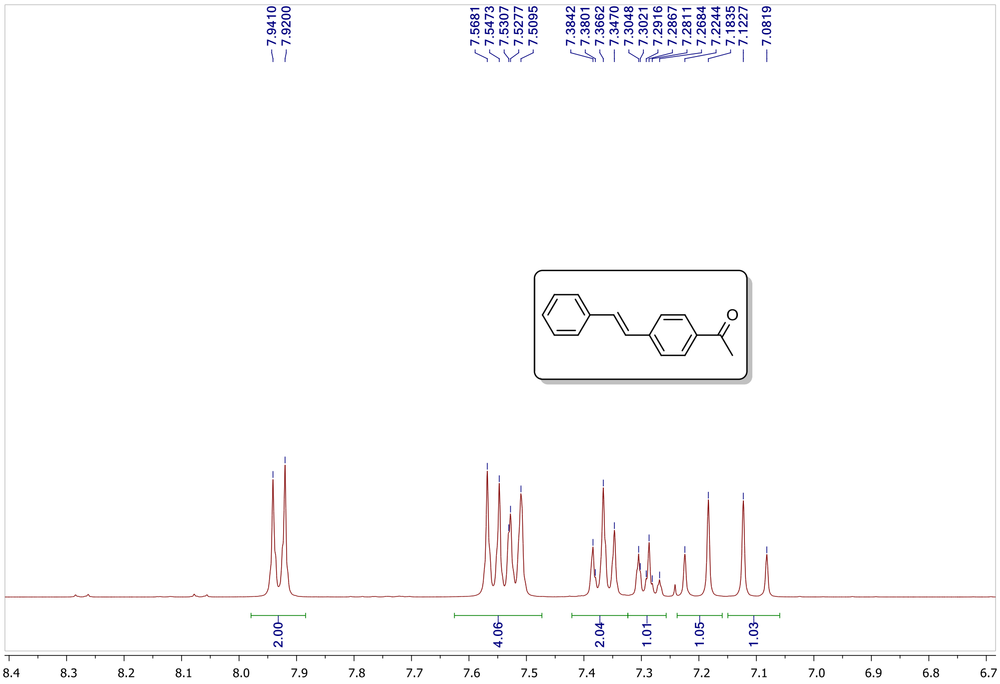

^{13}C NMR (101 MHz, $CDCl_3$) (*E*)-1-(4-styrylphenyl)ethanone (3)

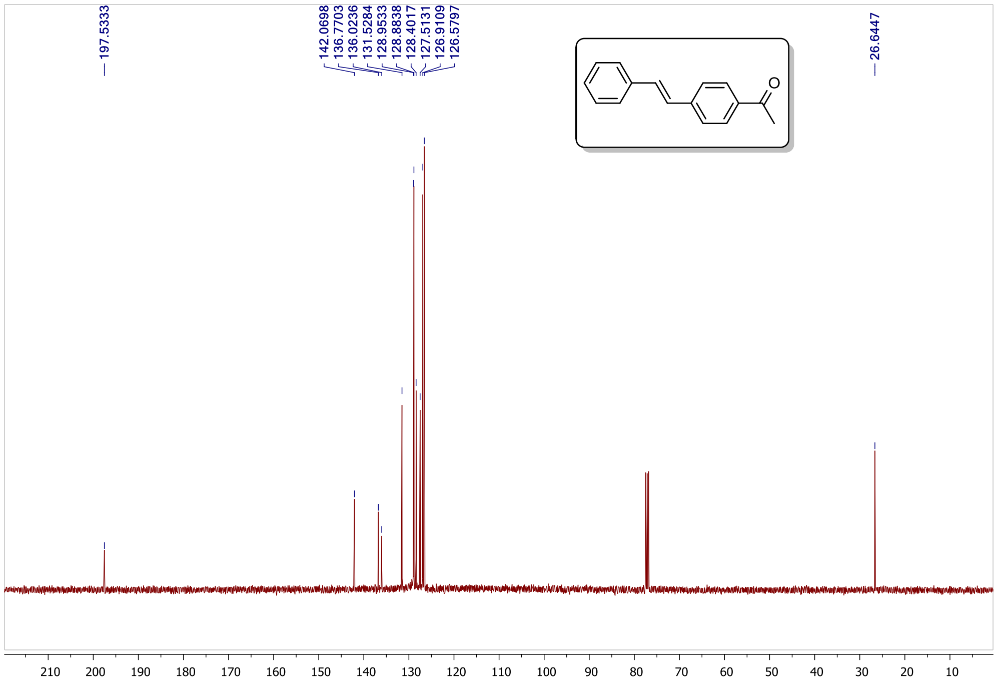

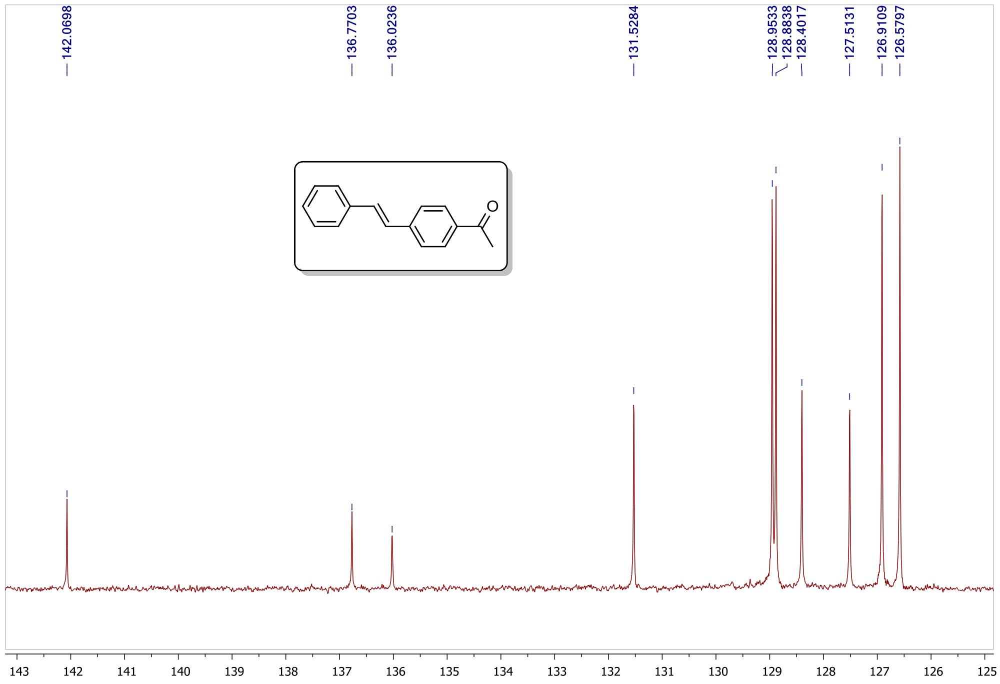

^{1}H-^{1}H COSY NMR (400 MHz, $CDCl_3$) (*E*)-1-(4-styrylphenyl)ethanone (3)

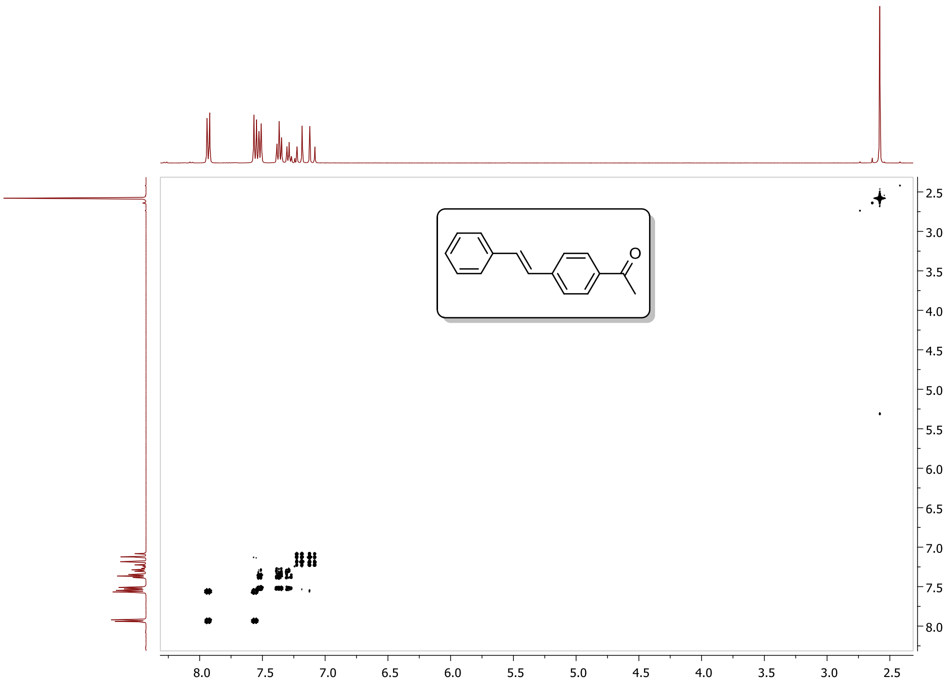

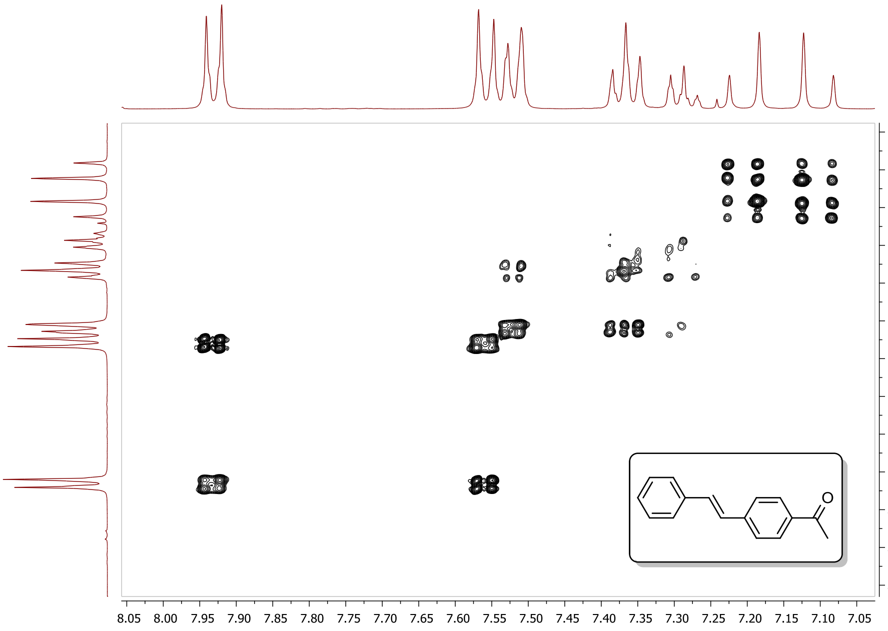

^{1}H-^{13}C HSQC NMR (400 MHz, $CDCl_3$) (*E*)-1-(4-styrylphenyl)ethanone (3)

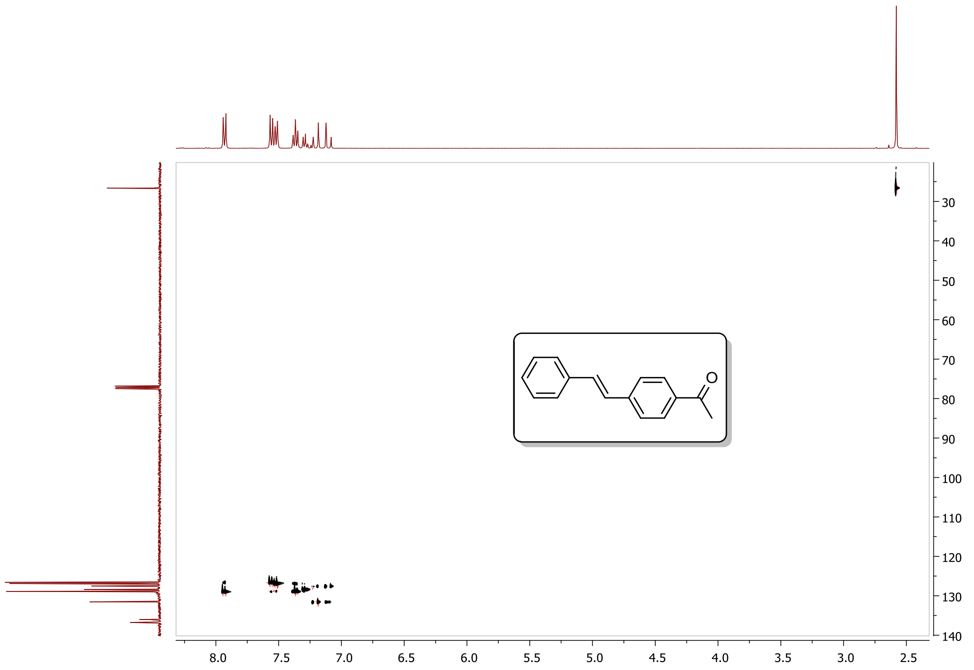

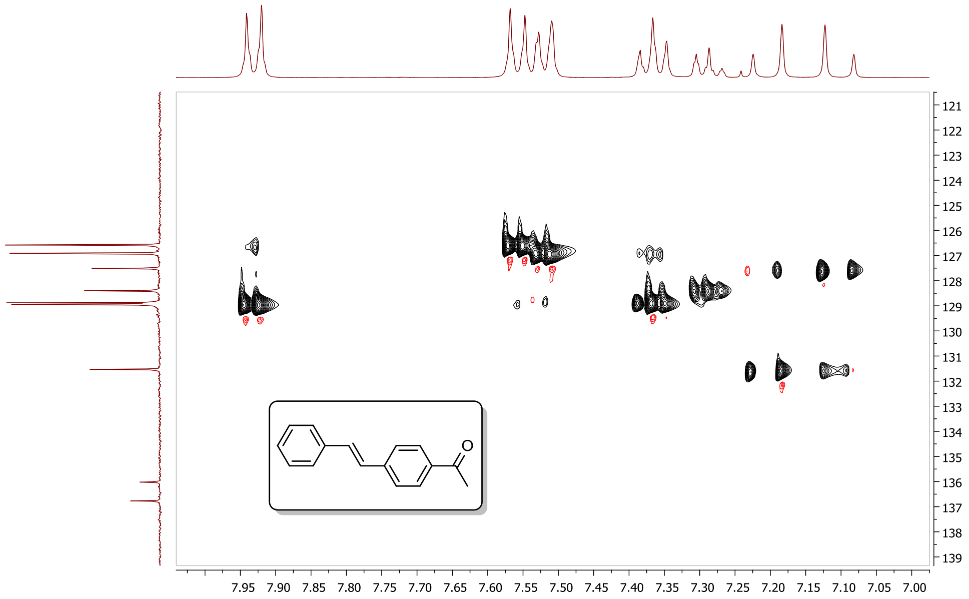

^{1}H-^{13}C HMBC NMR (400 MHz, $CDCl_3$) (*E*)-1-(4-styrylphenyl)ethanone (3)

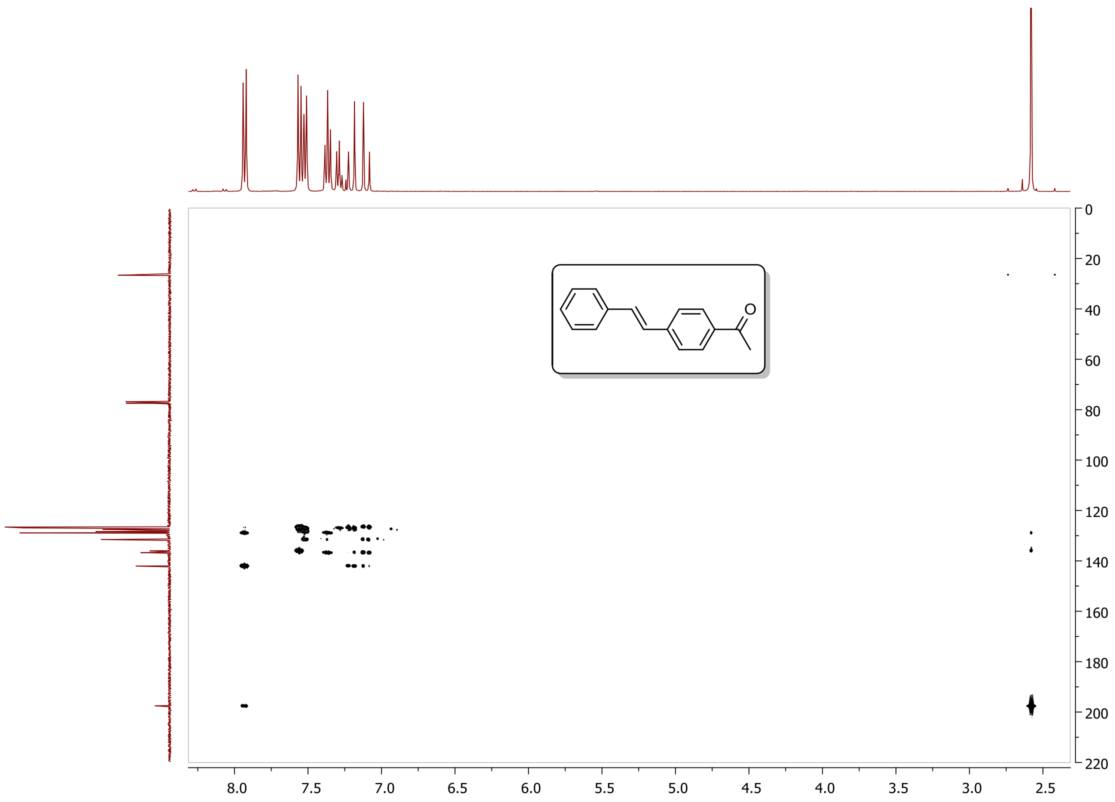

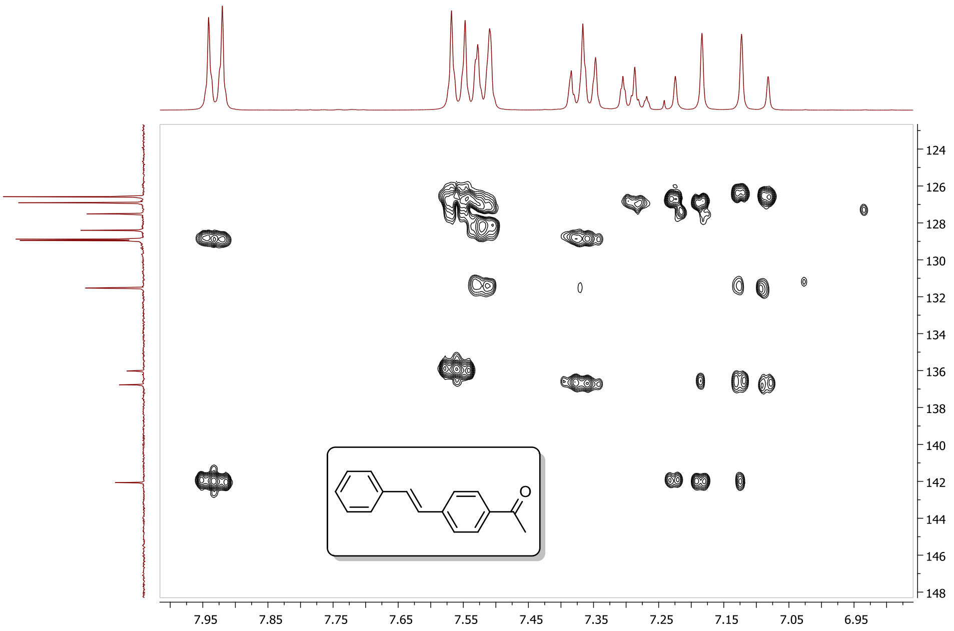

^{1}H NMR (400 MHz, $CDCl_3$) (*E*)-phenyl(4-styrylphenyl)methanone (4)

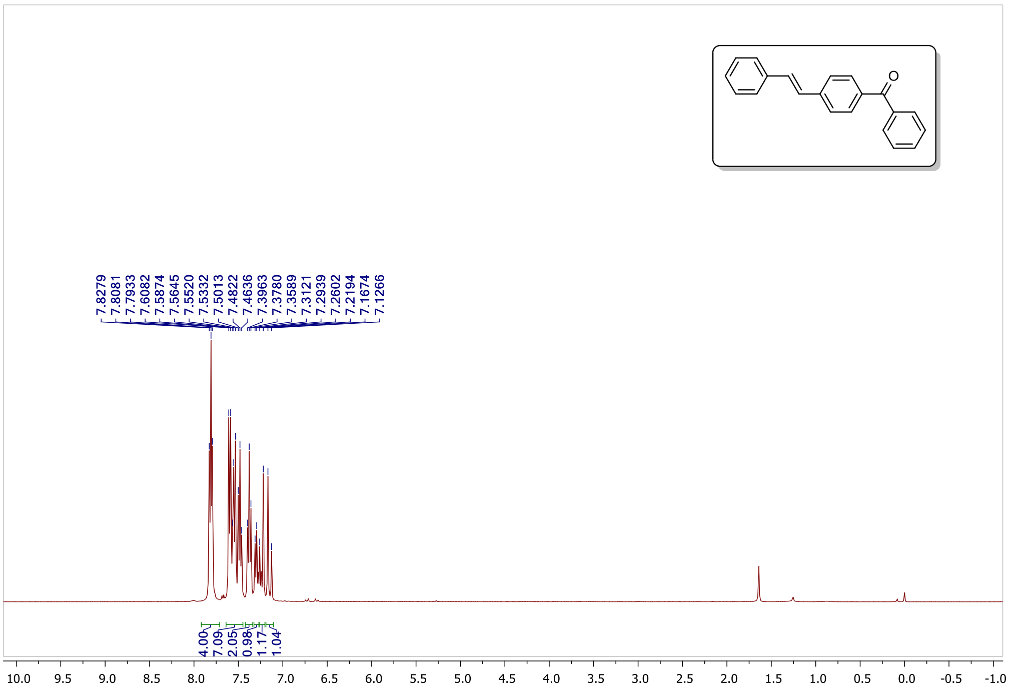

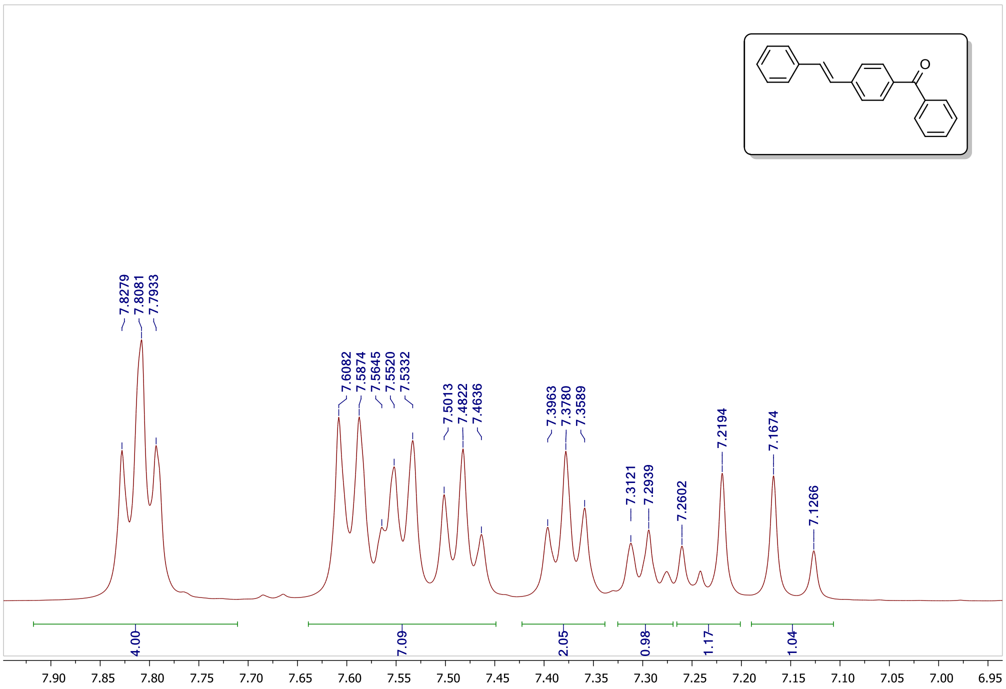

^{13}C NMR (101 MHz, $CDCl_3$) (*E*)-phenyl(4-styrylphenyl)methanone (4)

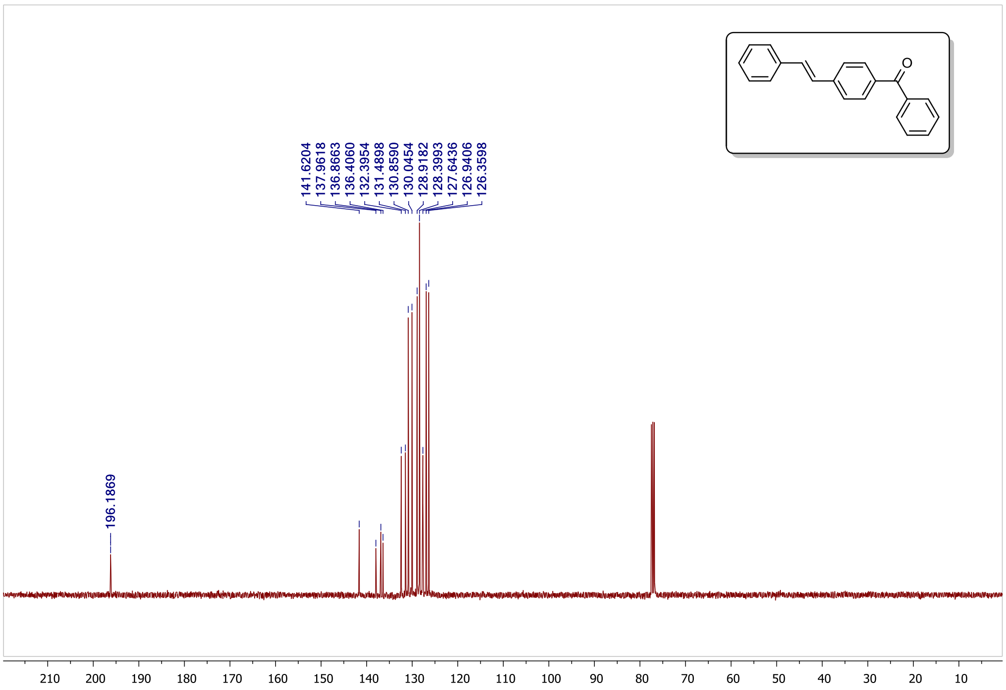

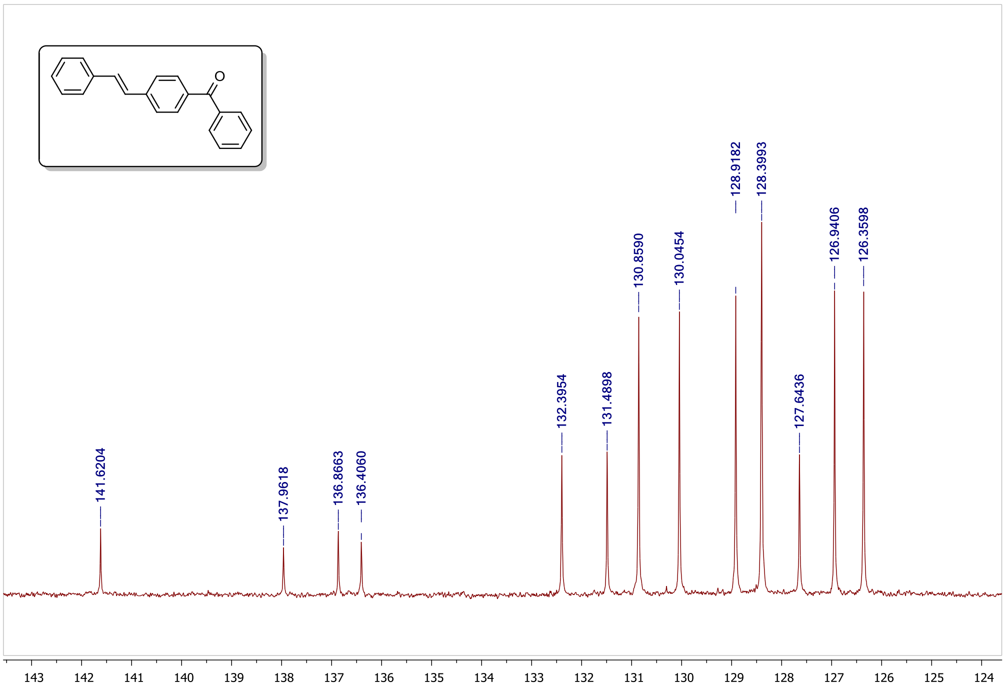

^{1}H-^{1}H COSY NMR (400 MHz, $CDCl_3$) (*E*)-phenyl(4-styrylphenyl)methanone (4)

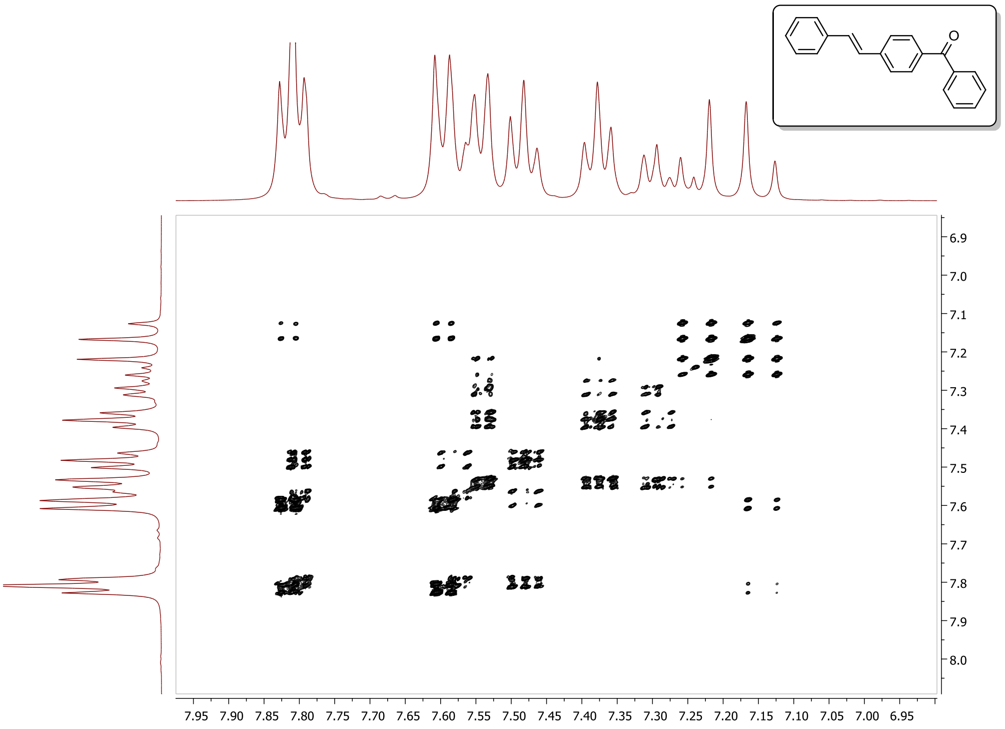

^{1}H-^{13}C HSQC NMR (400 MHz, $CDCl_3$) (*E*)-phenyl(4-styrylphenyl)methanone (4)

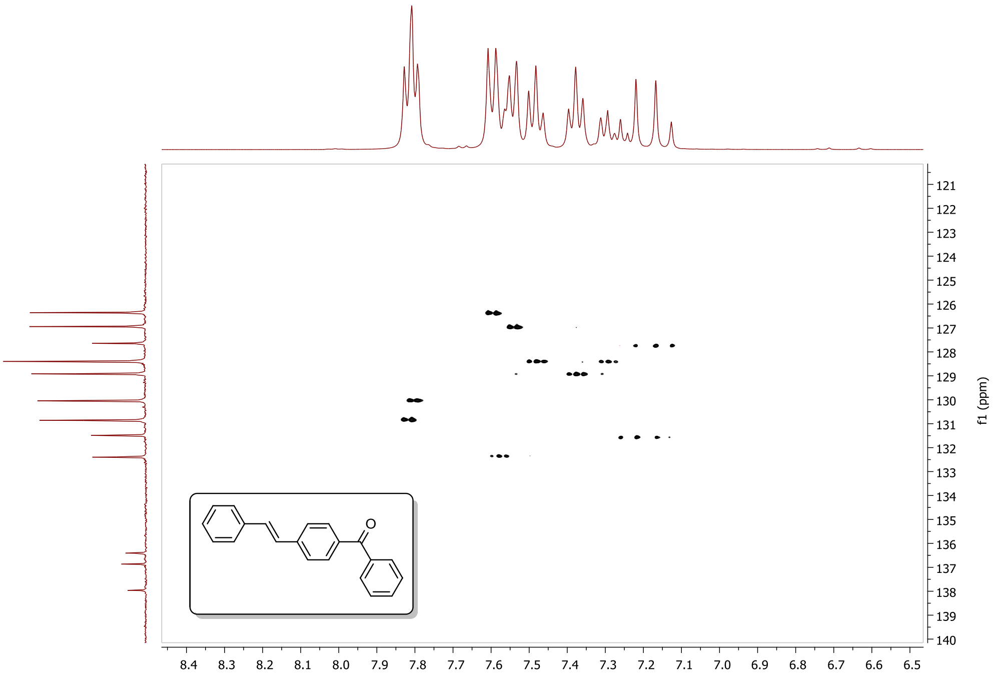

^{1}H-^{13}C HMBC NMR (400 MHz, $CDCl_3$) (*E*)-phenyl(4-styrylphenyl)methanone (4)

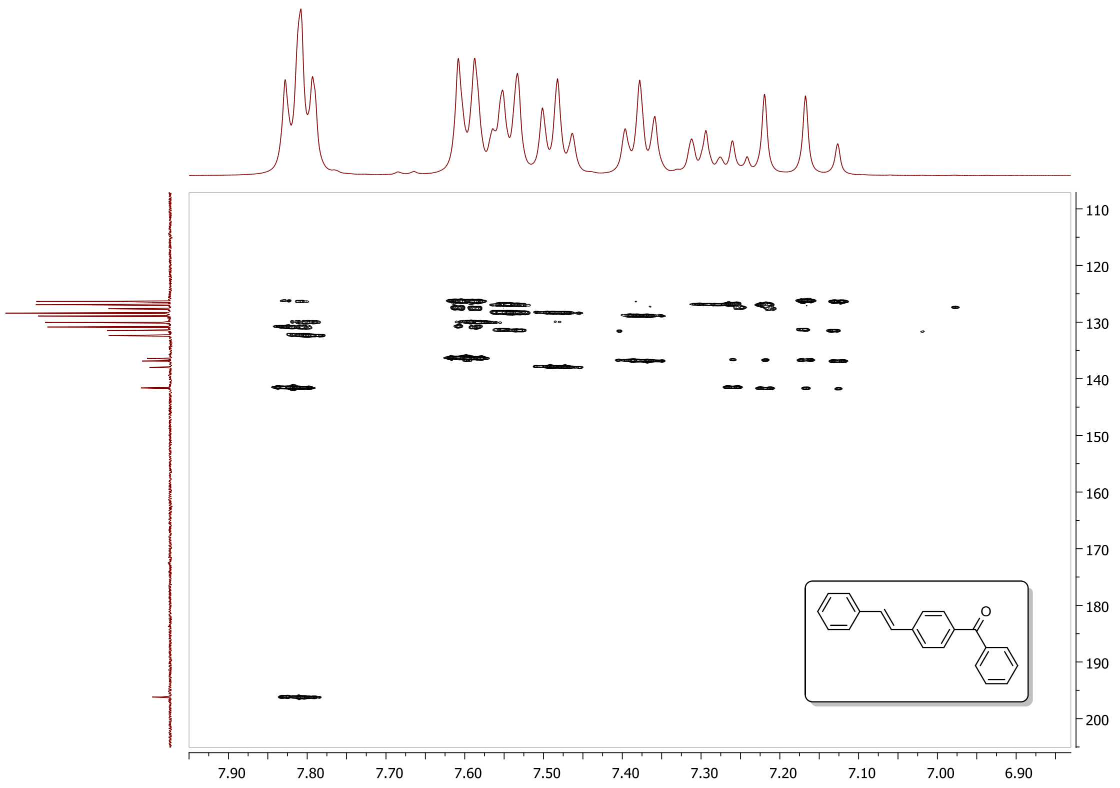

^{1}H NMR (400 MHz, $CDCl_3$) *(E)*-3-styrylquinoline (5)

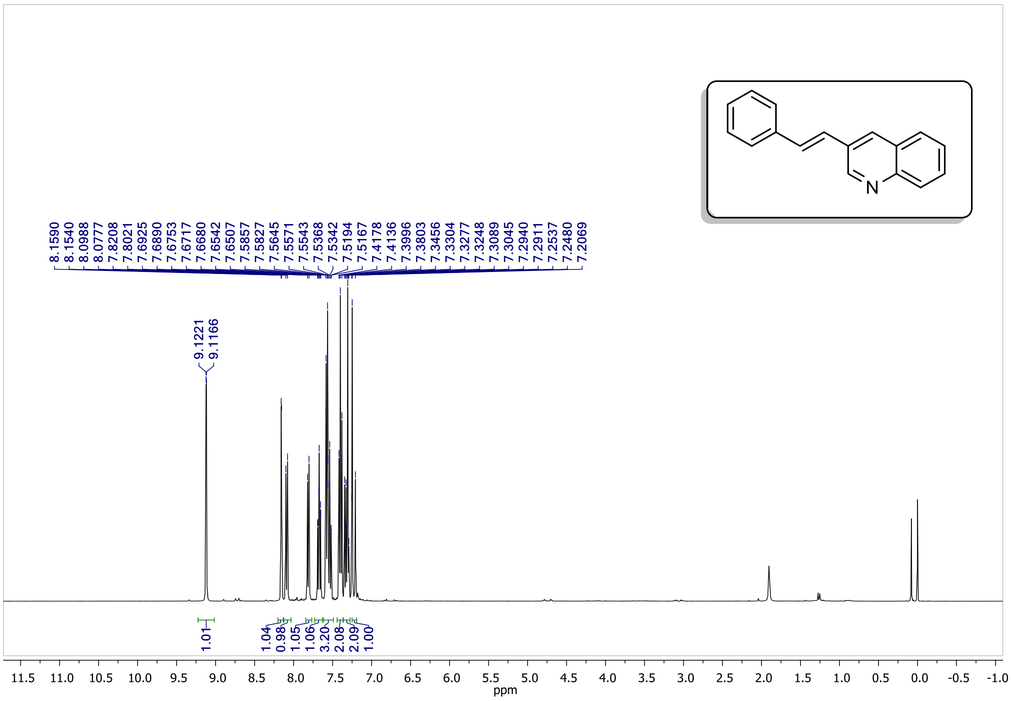

^{1}H NMR (400 MHz, $CDCl_3$) *(E)*-3-styrylquinoline (5)

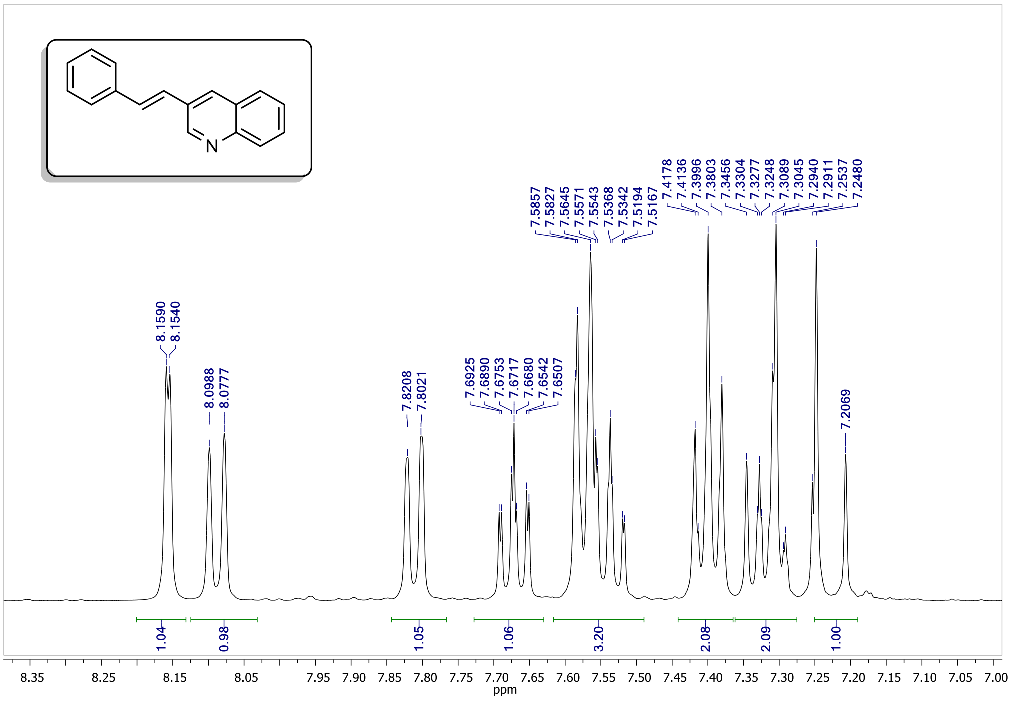

^{13}C NMR (100 MHz, $CDCl_3$) *(E)*-3-styrylquinoline (5)

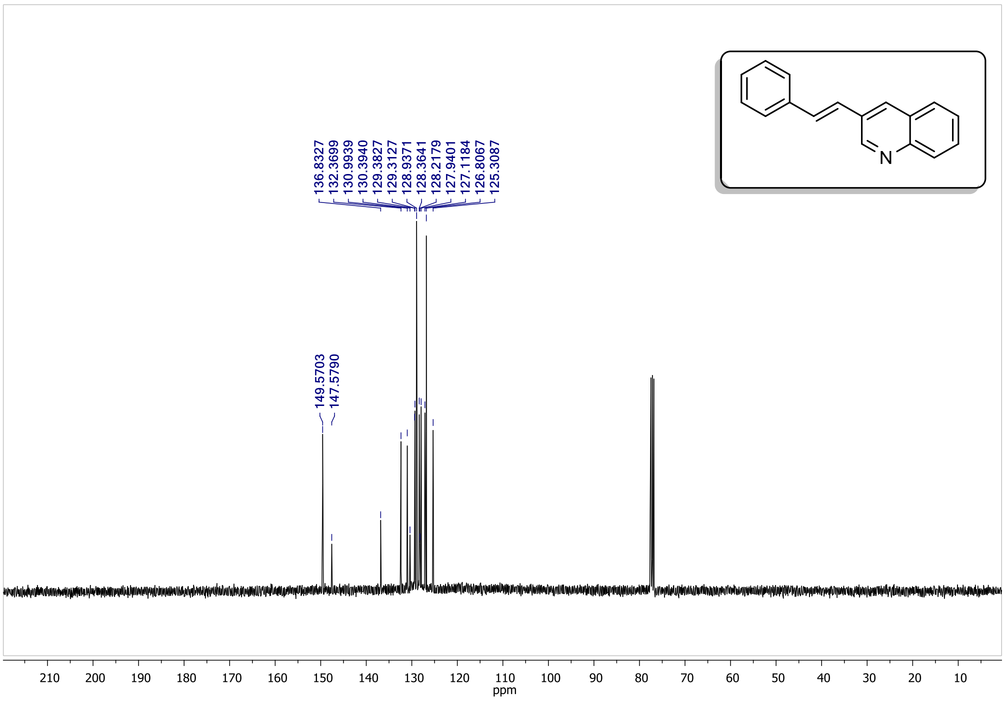

^{13}C NMR (100 MHz, $CDCl_3$) *(E)*-3-styrylquinoline (5)

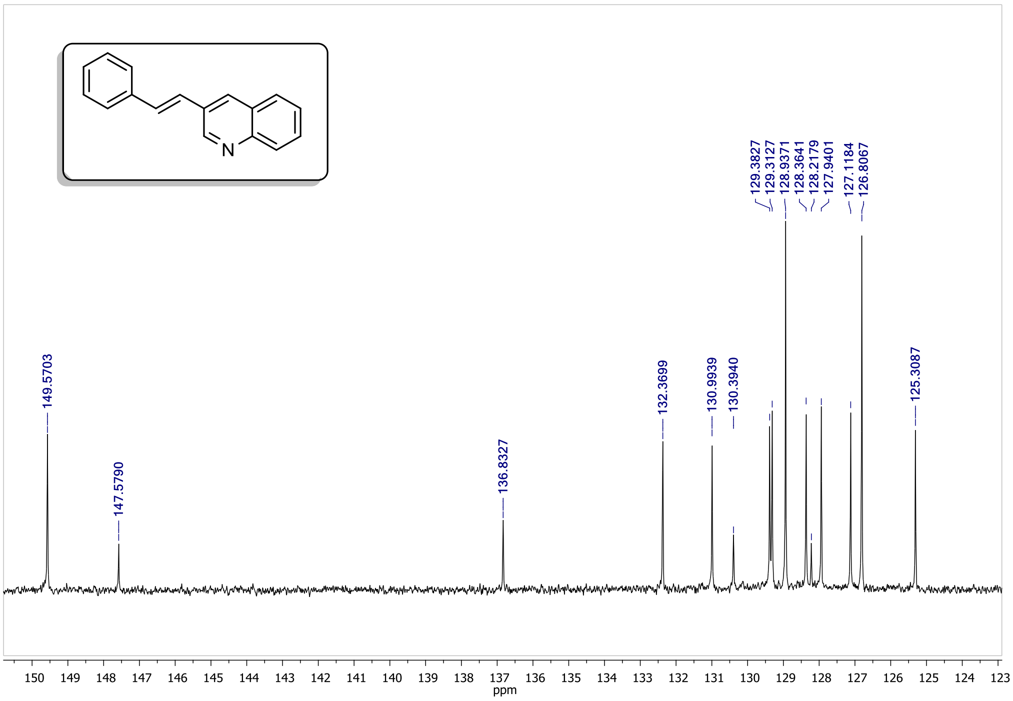

^{1}H-^{1}H COSY NMR (400 MHz, $CDCl_3$) *(E)*-3-styrylquinoline (5)

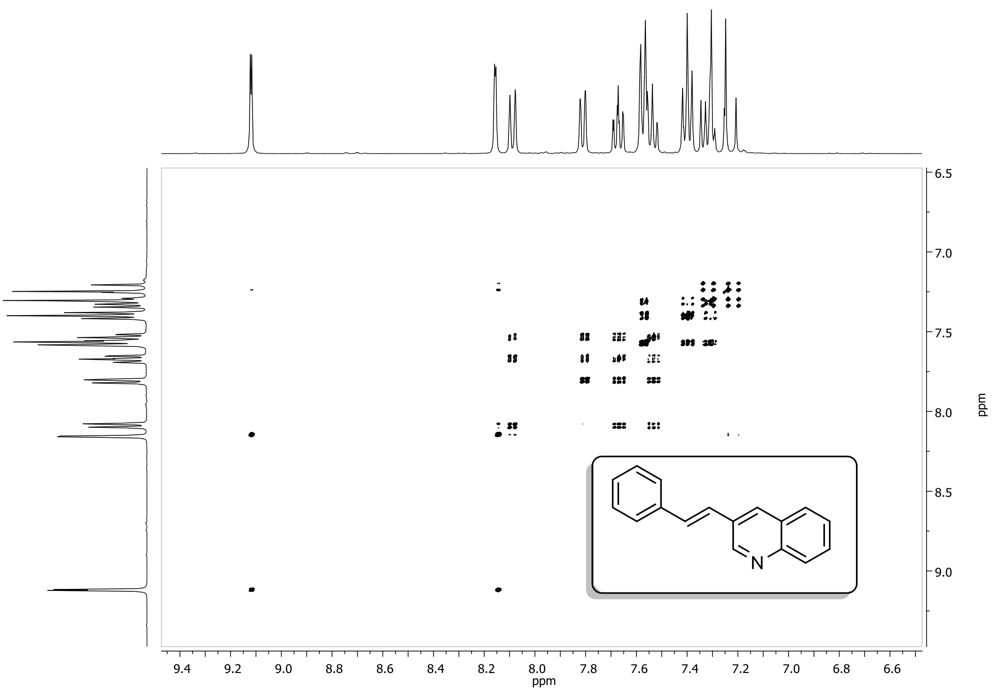

^{1}H-^{13}C HSQC NMR (400 MHz, $CDCl_3$) *(E)*-3-styrylquinoline (5)

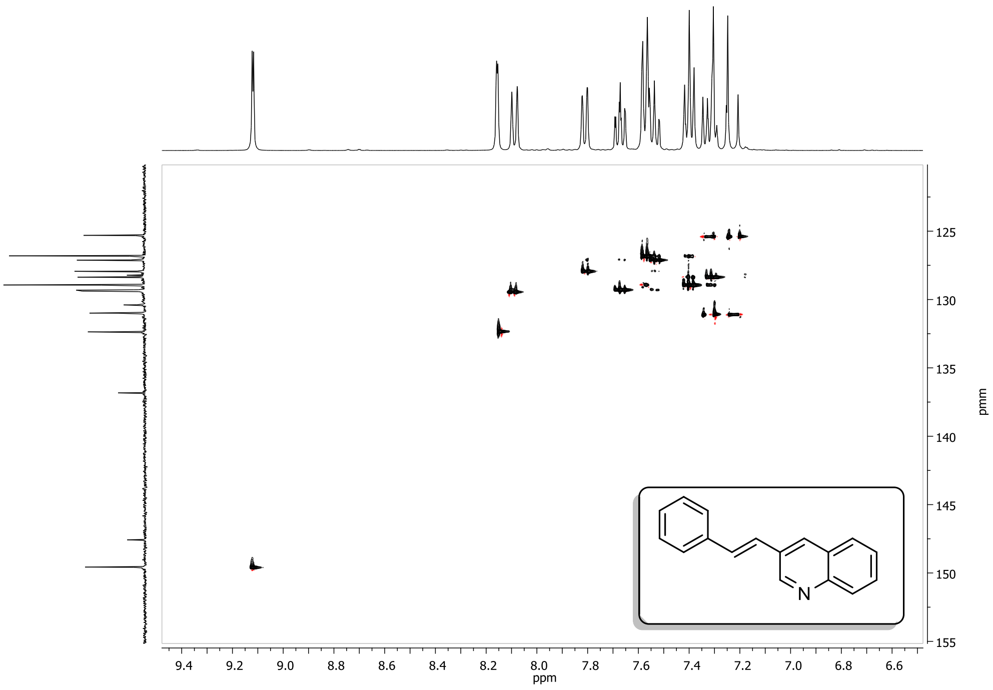

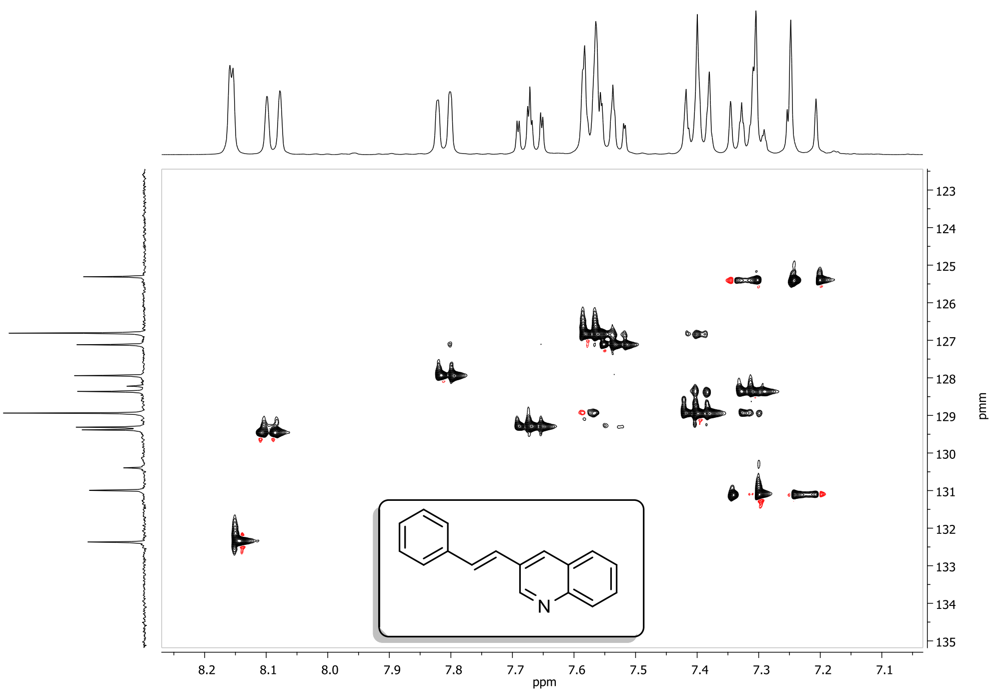

^{1}H-^{13}C HMBC NMR (400 MHz, $CDCl_3$) *(E)*-3-styrylquinoline (5)

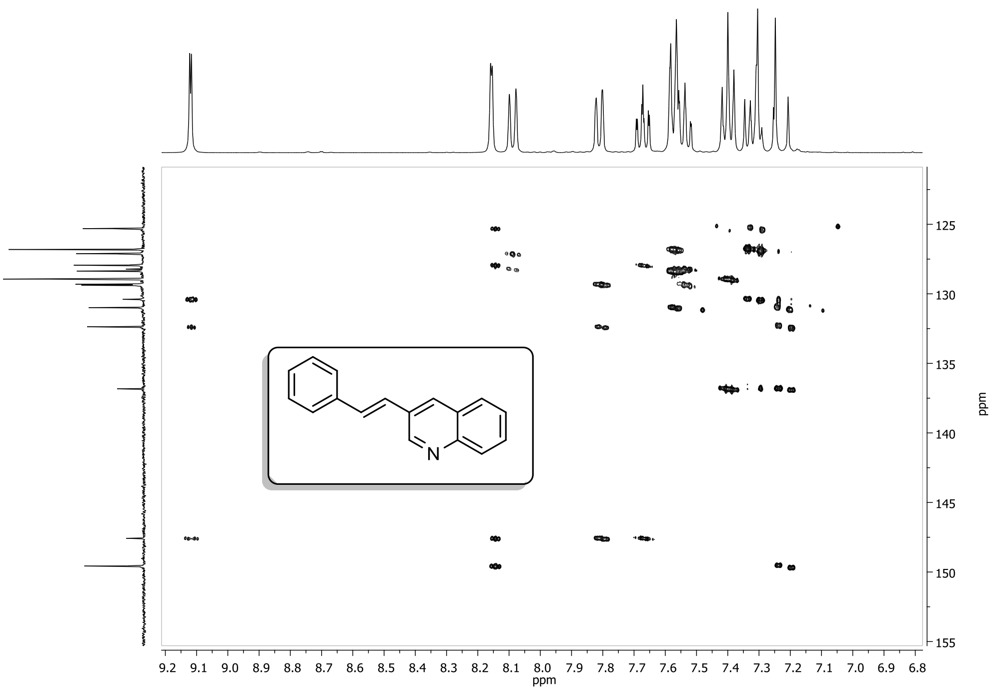

^{1}H NMR (400 MHz, $CDCl_3$) *(E)*-1,3-dimethoxy-5-styrylbenzene (6)

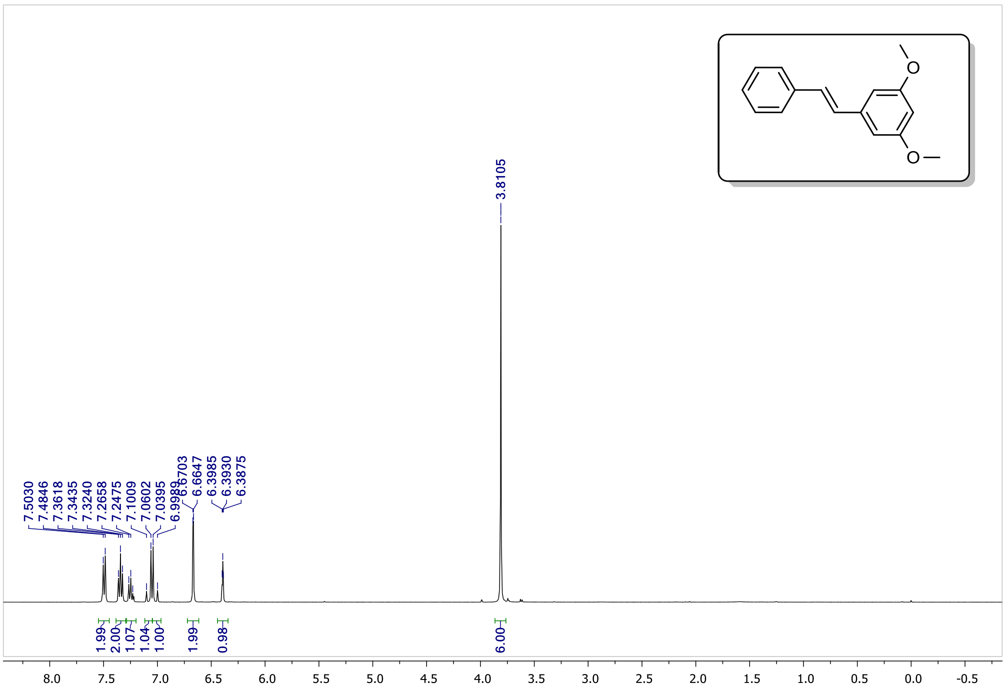

^{1}H NMR (400 MHz, $CDCl_3$) *(E)*-1,3-dimethoxy-5-styrylbenzene (6)

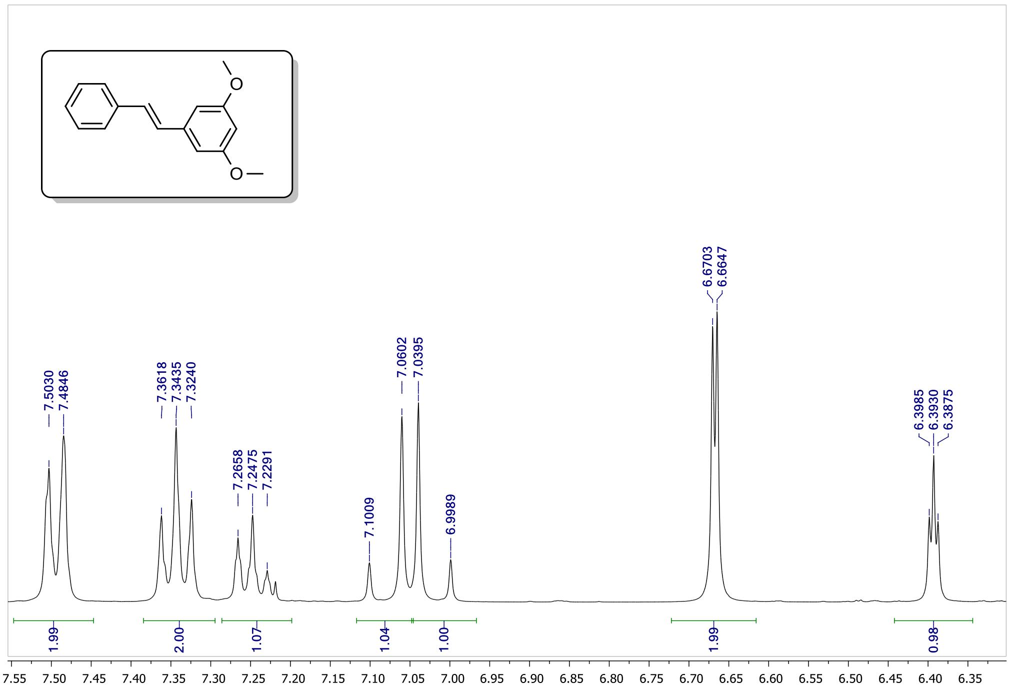

^{13}C NMR (101 MHz, CDCl$_3$) *(E)*-1,3-dimethoxy-5-styrylbenzene (6)

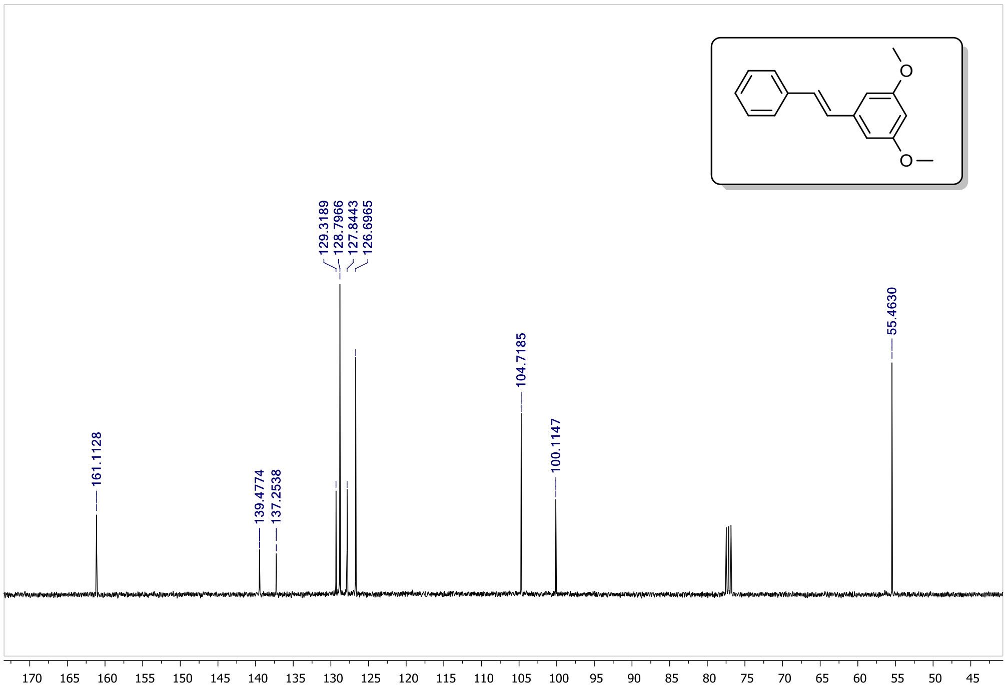

^{1}H-^{1}H COSY NMR (400 MHz, CDCl$_3$) *(E)*-1,3-dimethoxy-5-styrylbenzene (6)

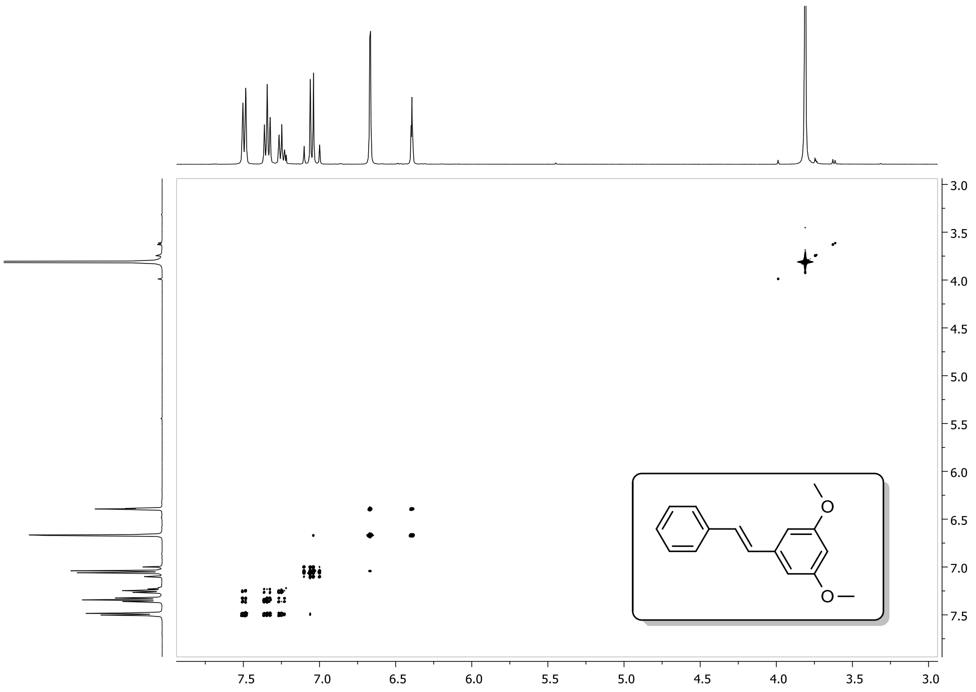

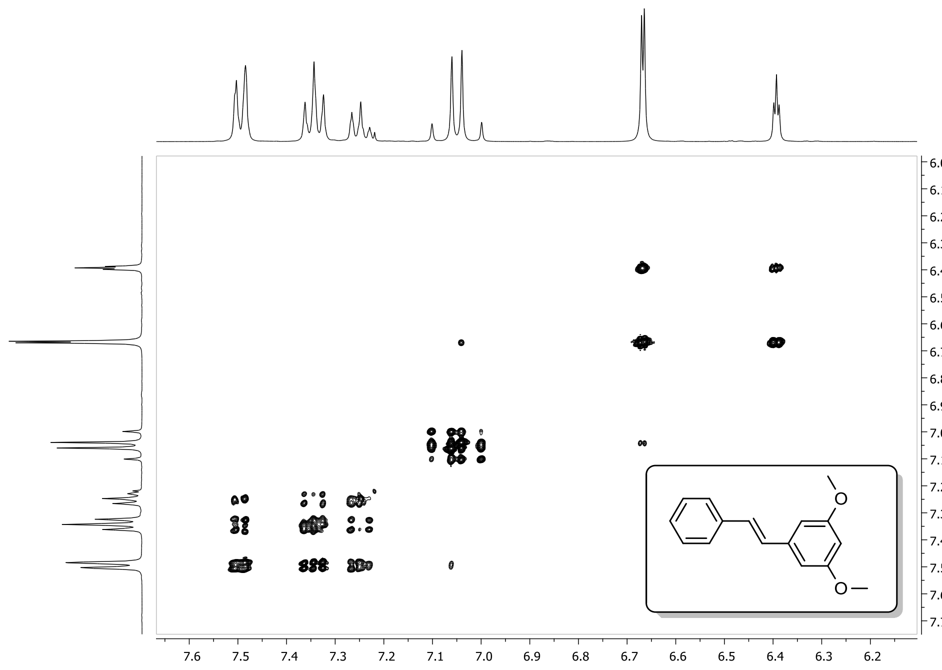

^{1}H-^{13}C HSQC NMR (400 MHz, $CDCl_3$) *(E)*-1,3-dimethoxy-5-styrylbenzene (6)

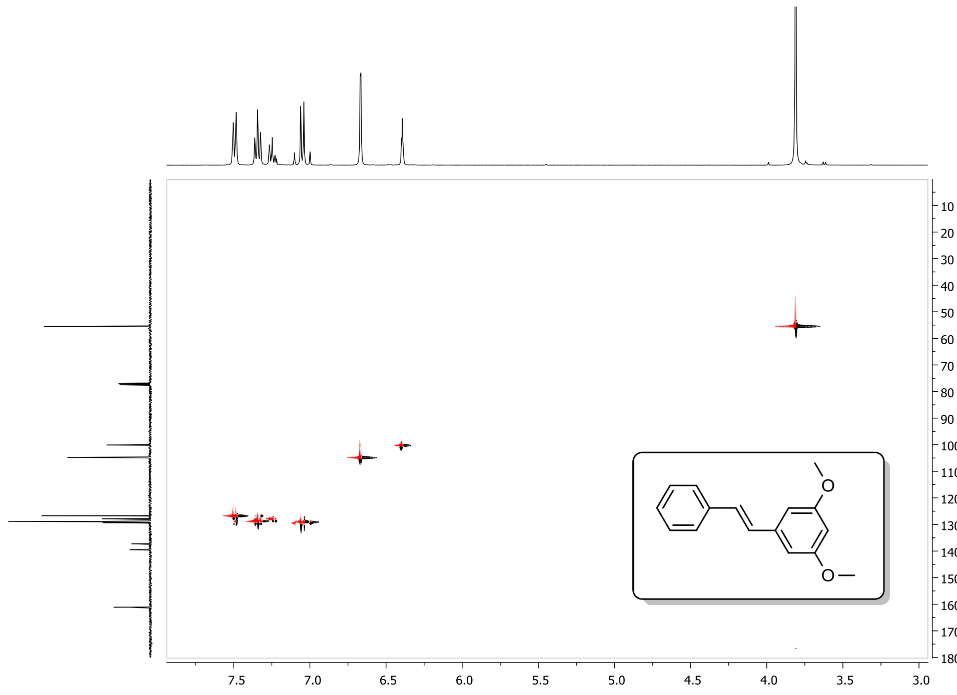

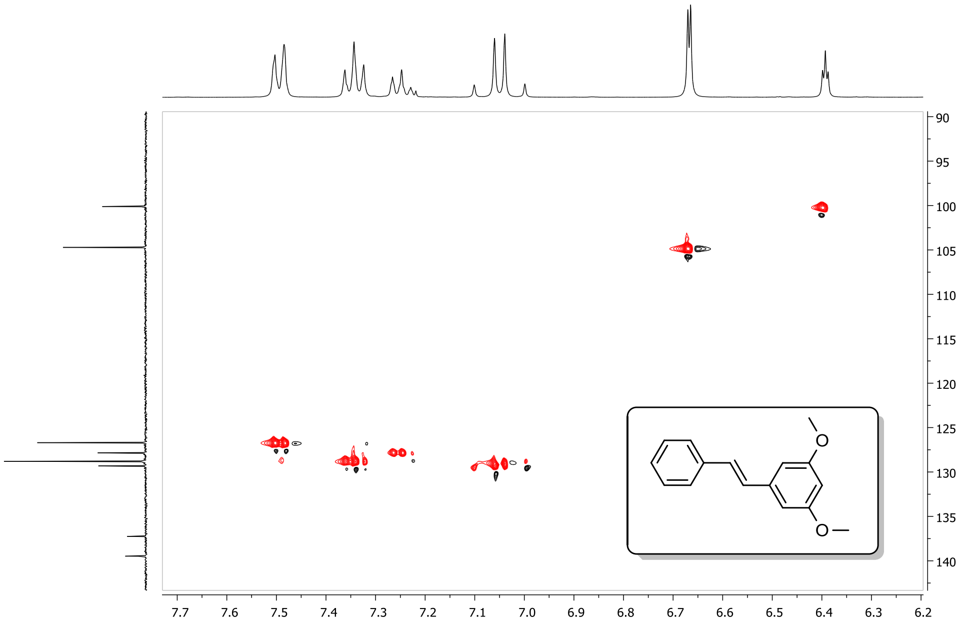

^{1}H-^{13}C HMBC NMR (400 MHz, $CDCl_3$) *(E)*-1,3-dimethoxy-5-styrylbenzene (6)

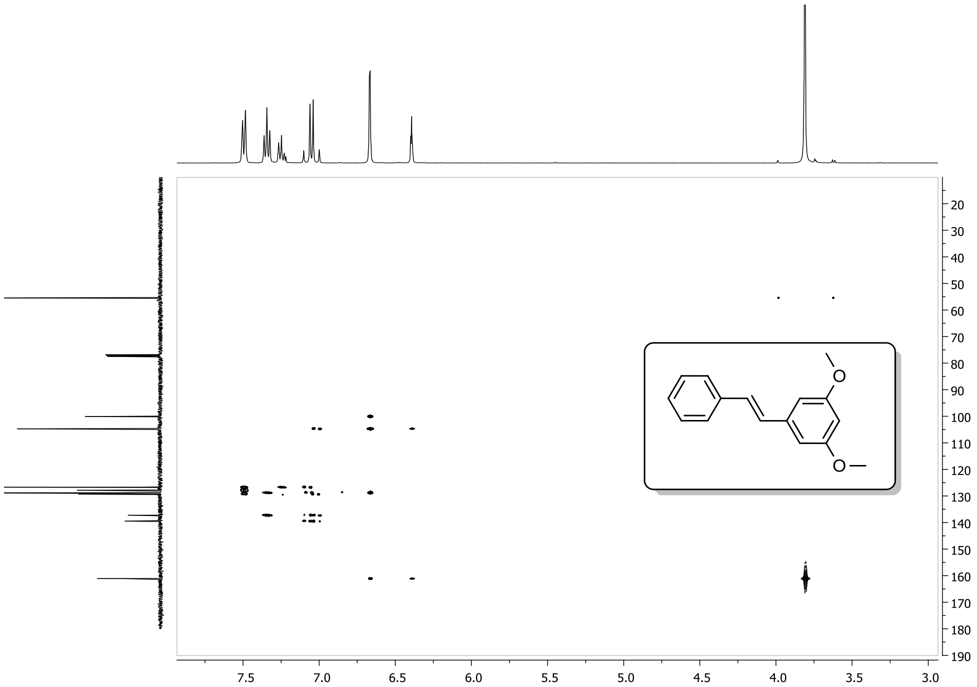

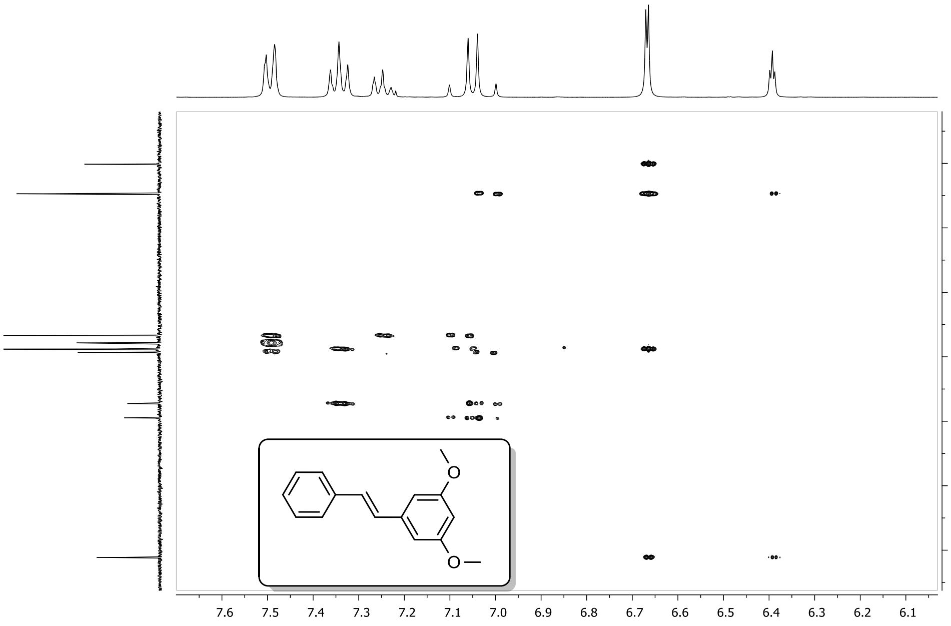

100
110
120
130
140
150
160
7.6
7.5
7.4
7.3
7.2
7.1
7.0
6.9
6.8
6.7
6.6
6.5
6.4
6.3
6.2
6.1
O
O

^{1}H NMR (400 MHz, $CDCl_3$) (*E*)-1-methoxy-4-styrylbenzene (7)

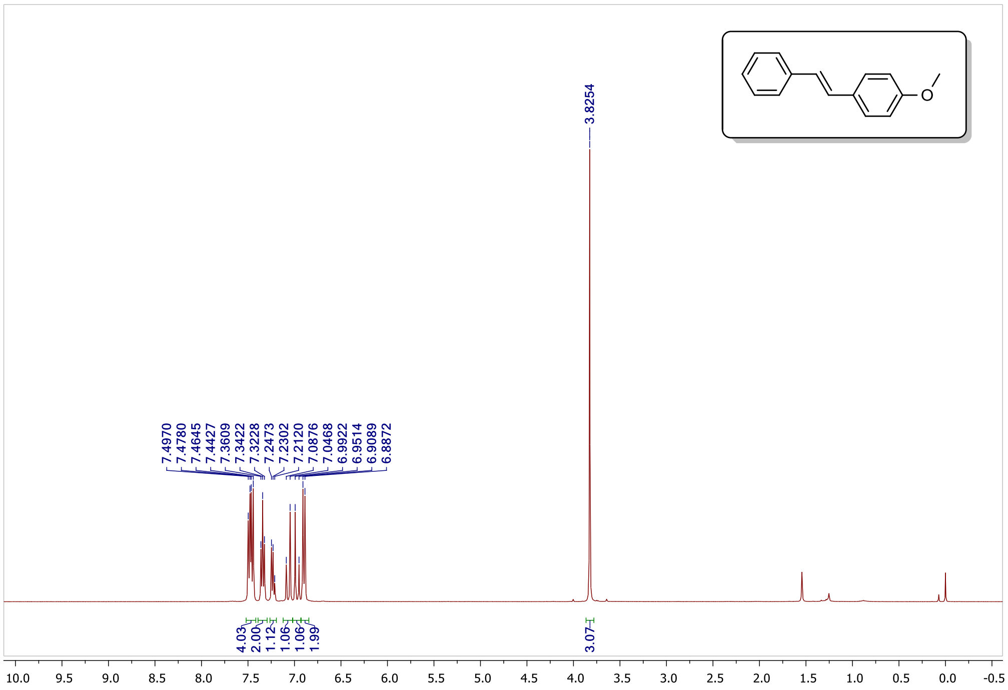

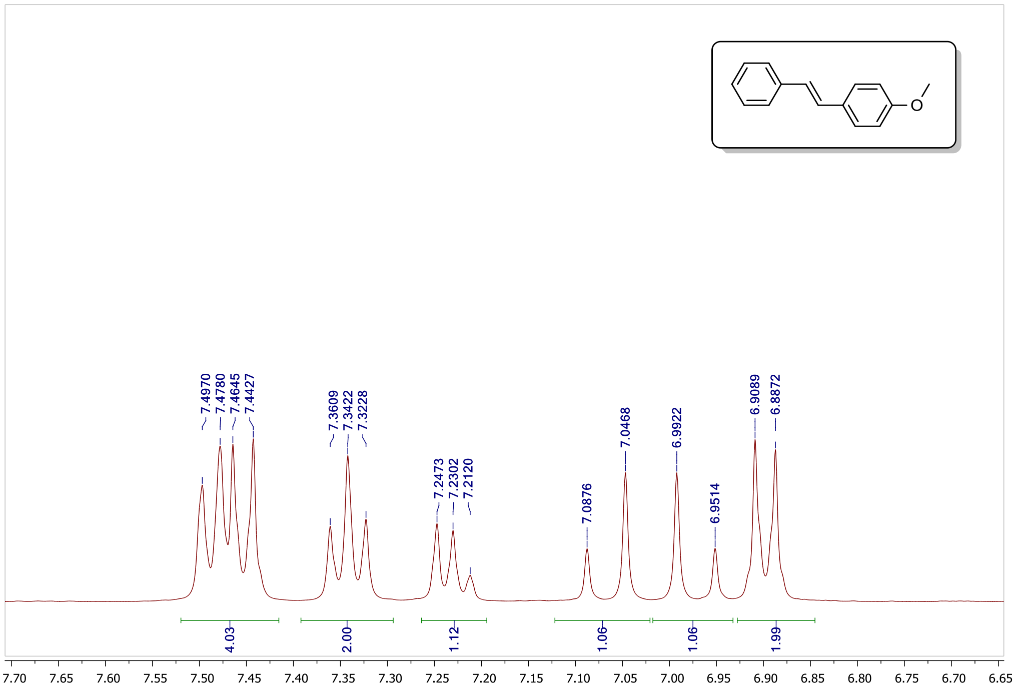

^{13}C NMR (101 MHz, $CDCl_3$) (*E*)-1-methoxy-4-styrylbenzene (7)

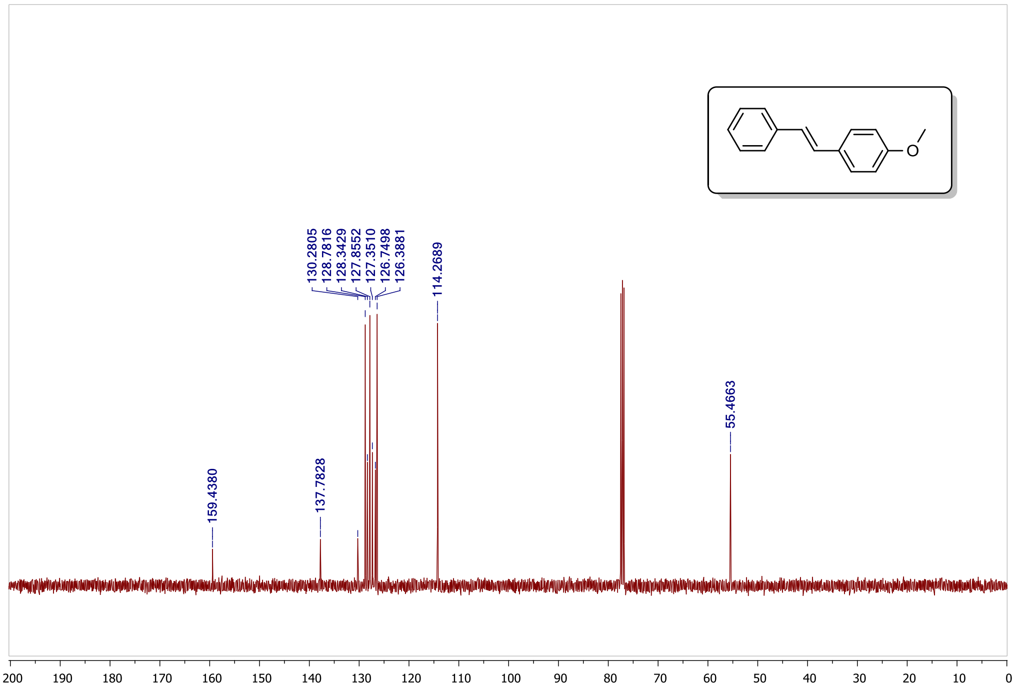

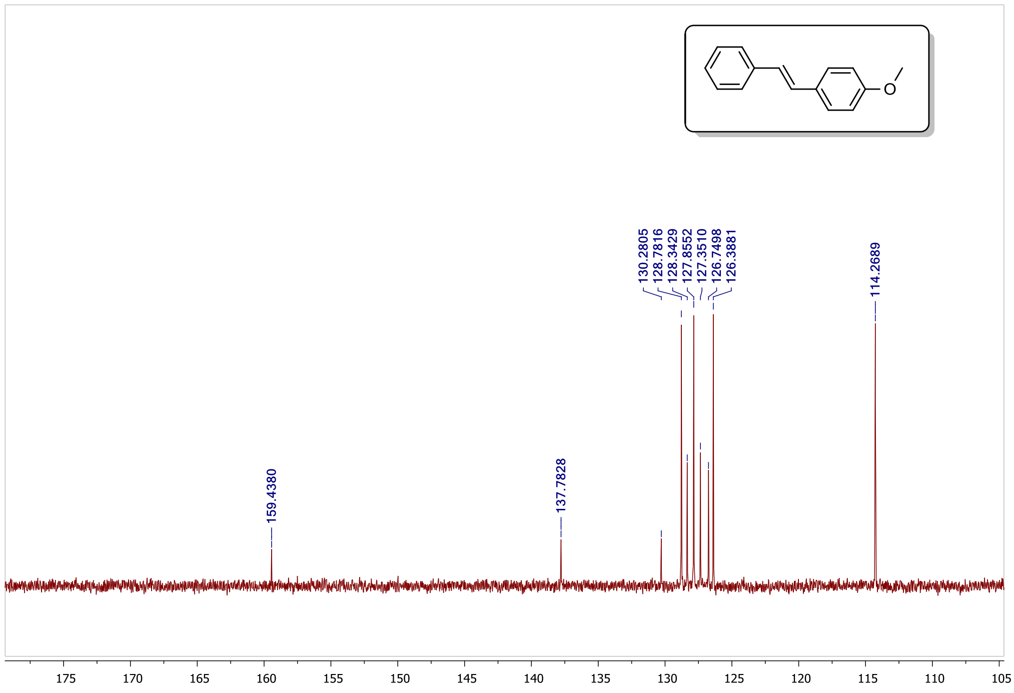

^{1}H-^{1}H COSY NMR (400 MHz, $CDCl_3$) (*E*)-1-methoxy-4-styrylbenzene (7)

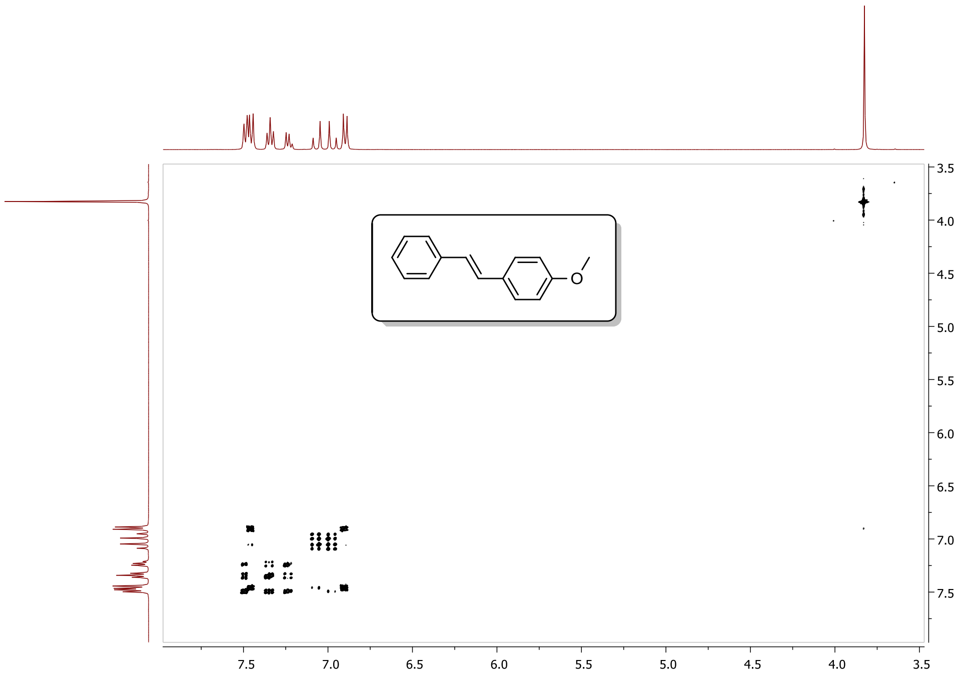

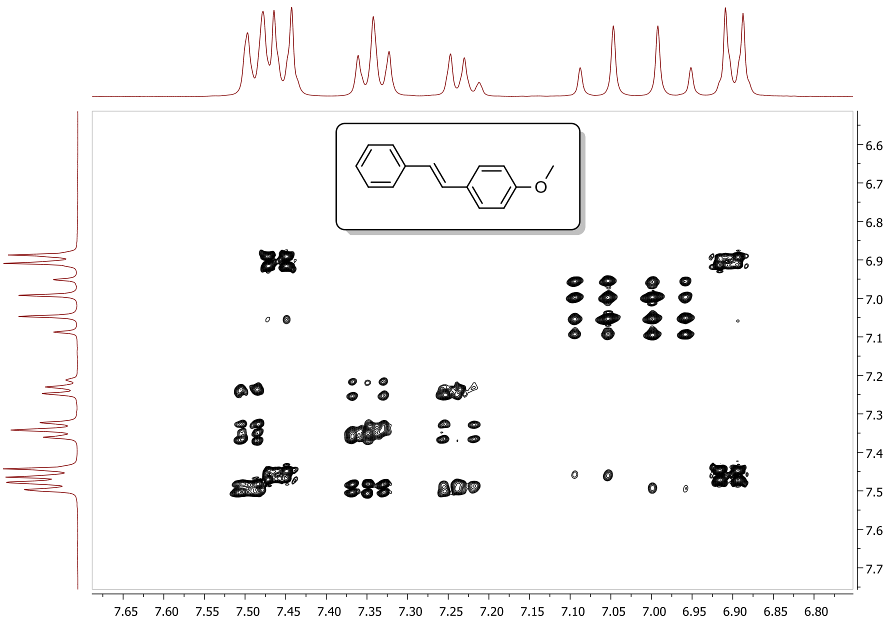

^{1}H-^{13}C HSQC NMR (400 MHz, $CDCl_3$) (*E*)-1-methoxy-4-styrylbenzene (7)

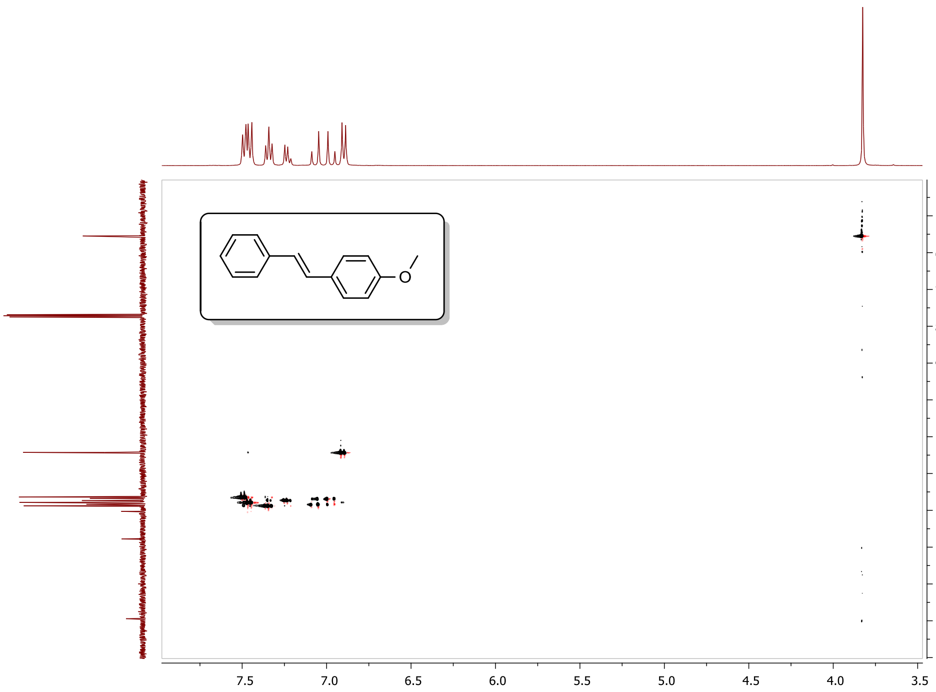

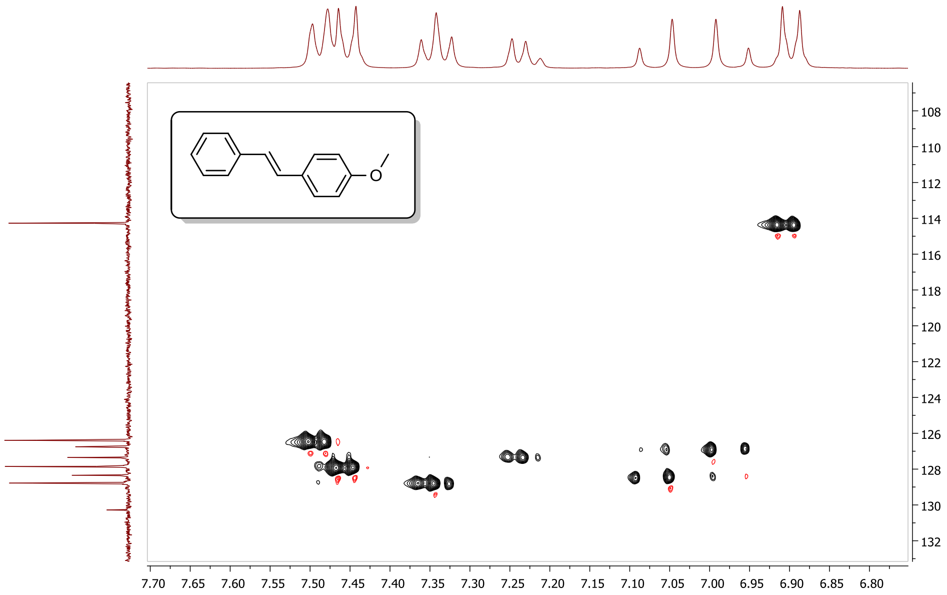

^{1}H-^{13}C HMBC NMR (400 MHz, $CDCl_3$) (*E*)-1-methoxy-4-styrylbenzene (7)

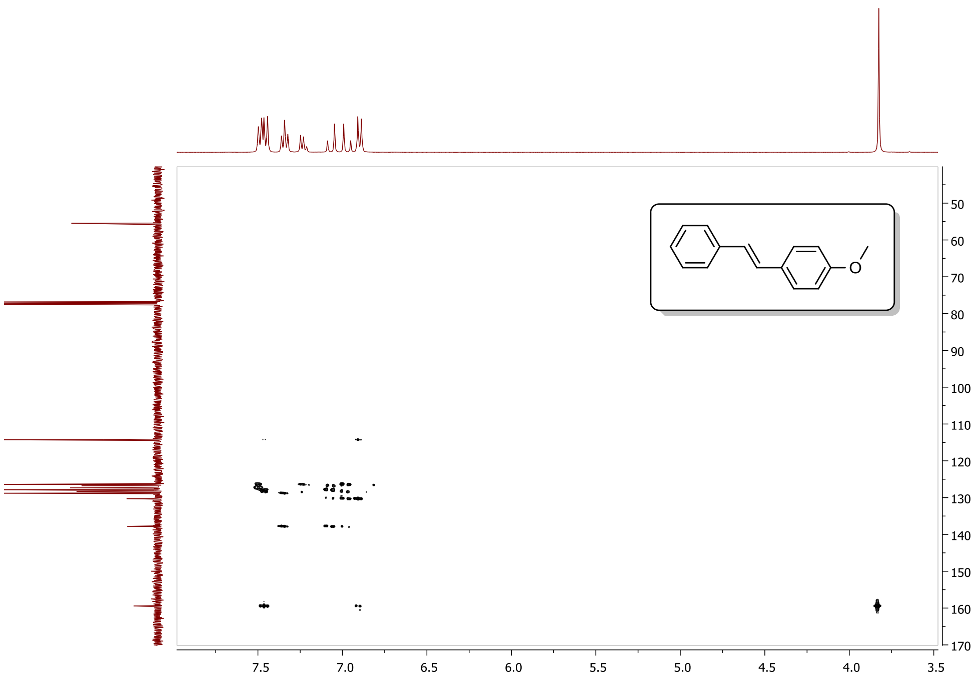

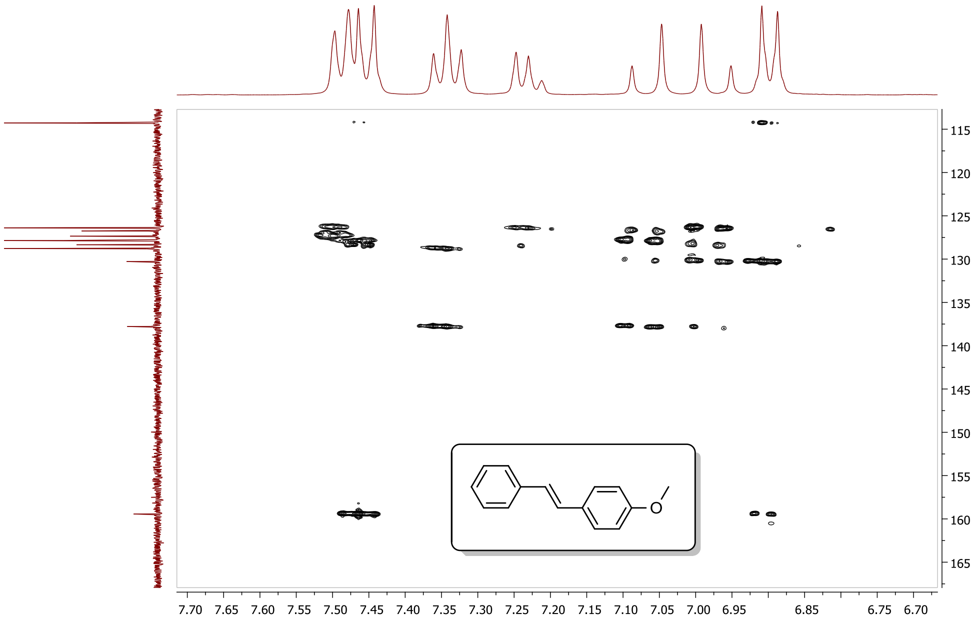

^{1}H NMR (400 MHz, CDCl$_3$) *(E)*-1-methyl-4-styrylbenzene (8)

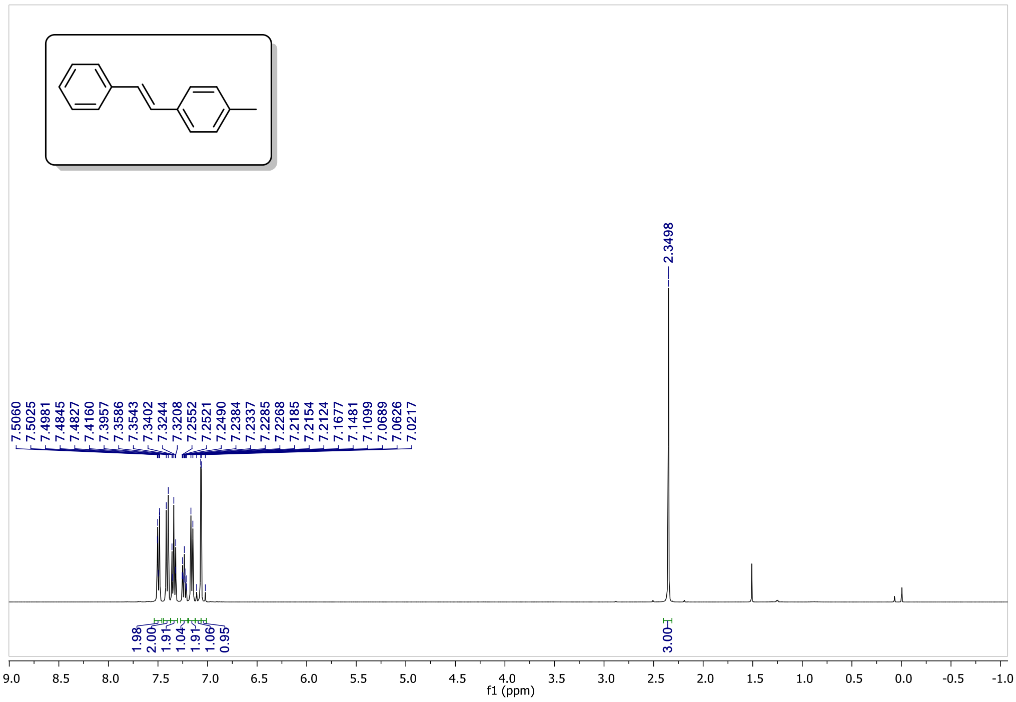

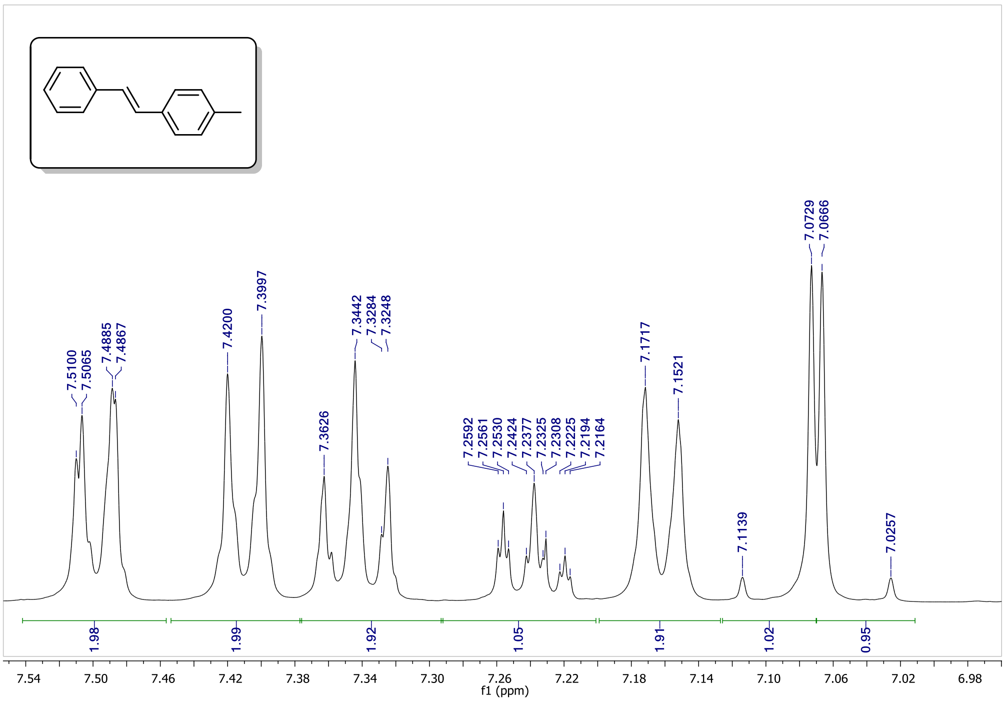

^{13}C NMR (100 MHz, $CDCl_3$) *(E)*-1-methyl-4-styrylbenzene (8)

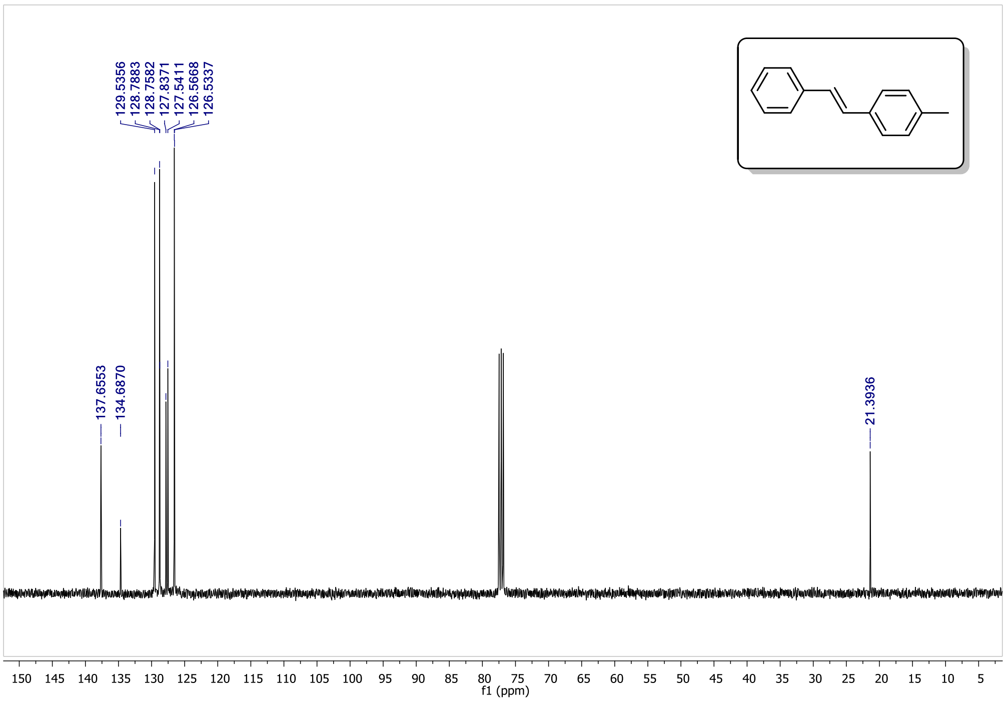

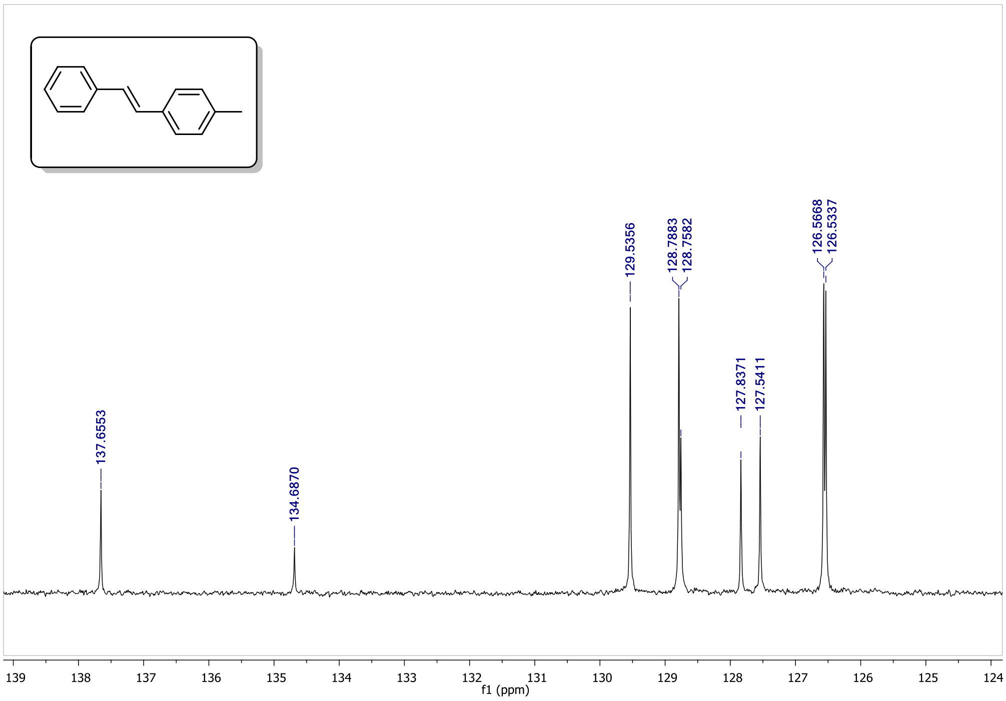

^{1}H-^{1}H COSY NMR (400 MHz, $CDCl_3$) *(E)*-1-methyl-4-styrylbenzene (8)

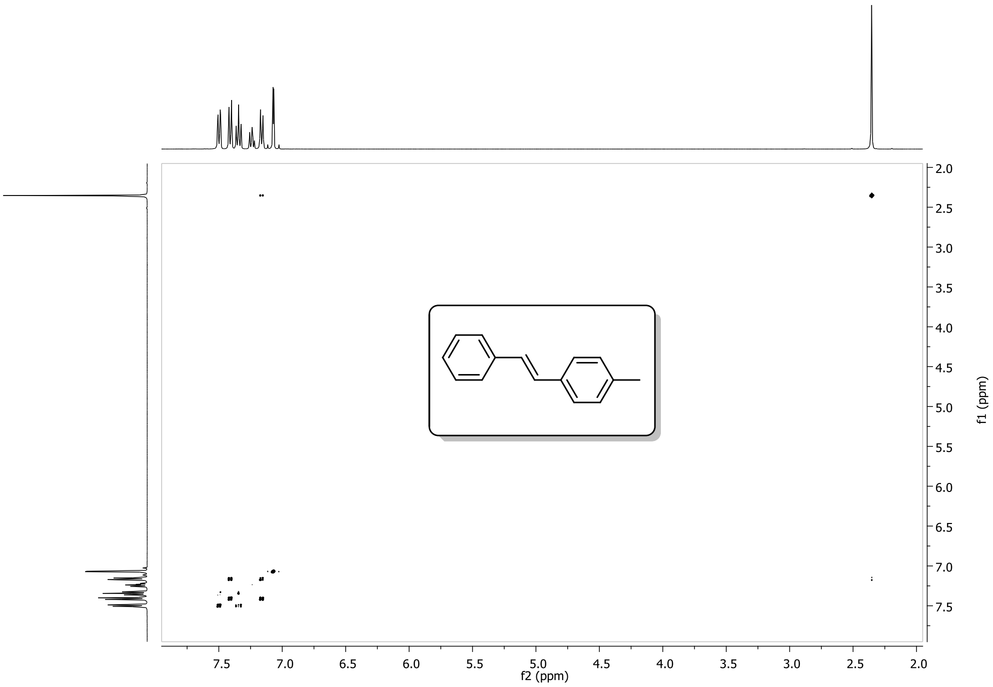

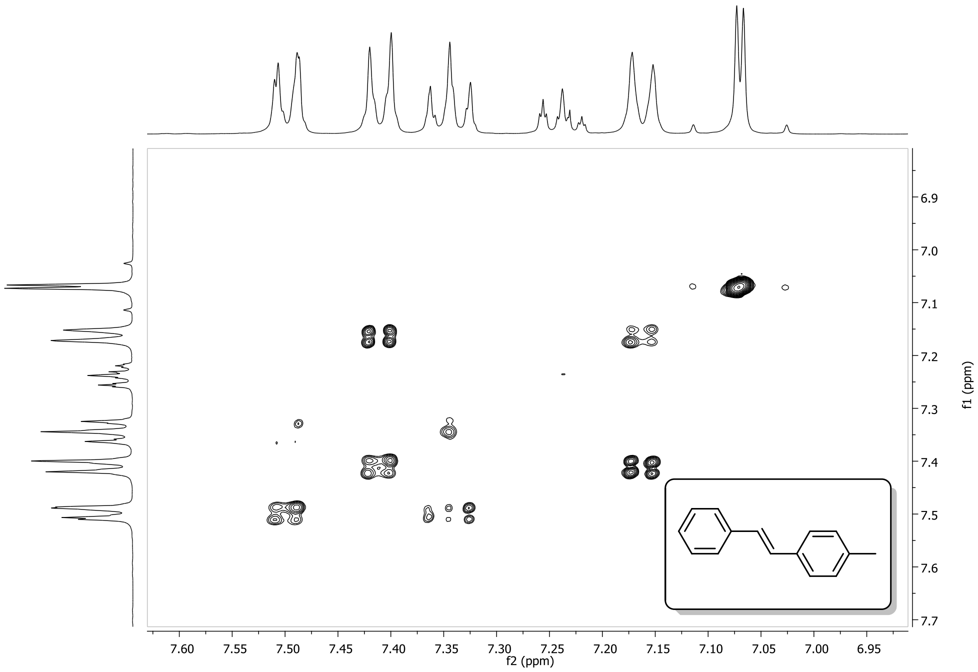

^{1}H-^{13}C HSQC NMR (400 MHz, $CDCl_3$) *(E)*-1-methyl-4-styrylbenzene (8)

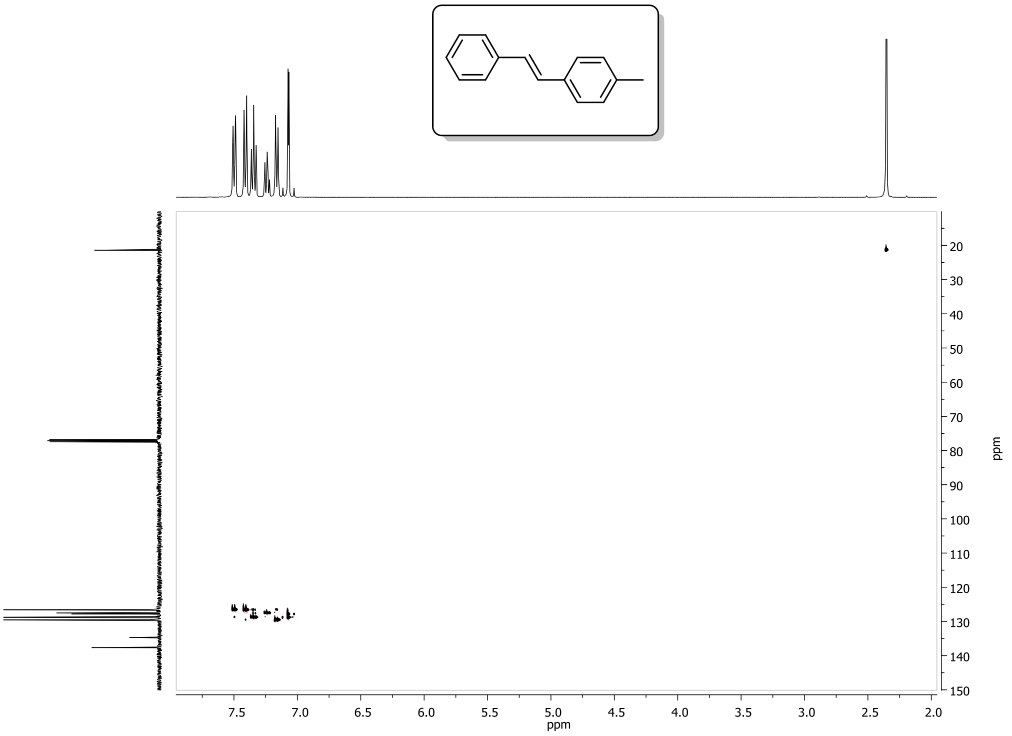

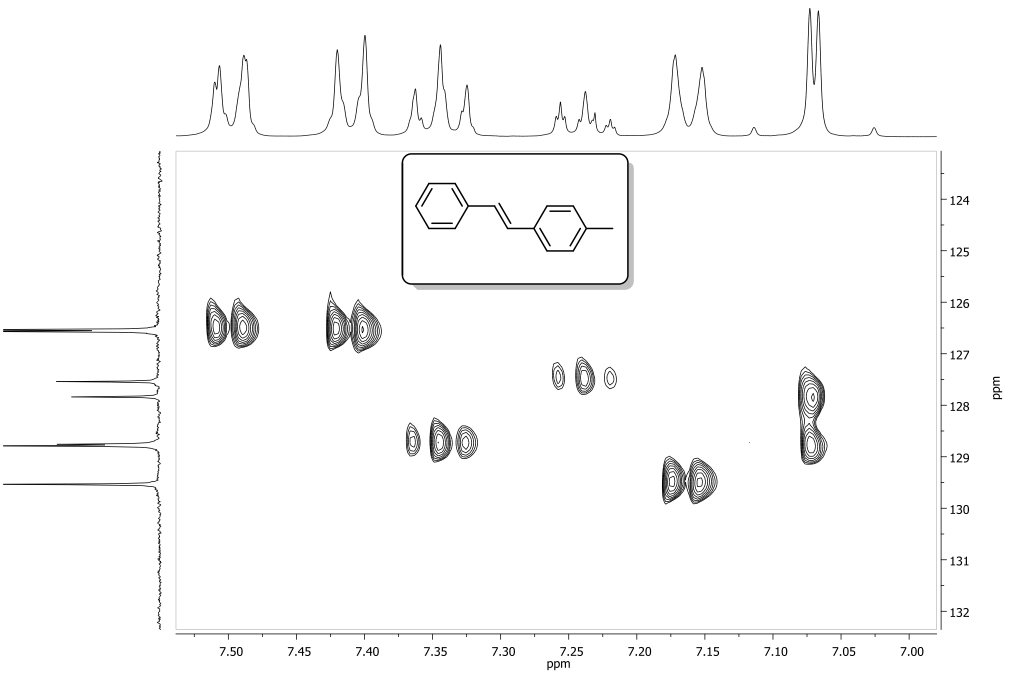

^{1}H-^{13}C HMBC NMR (400 MHz, $CDCl_3$) *(E)*-1-methyl-4-styrylbenzene (8)

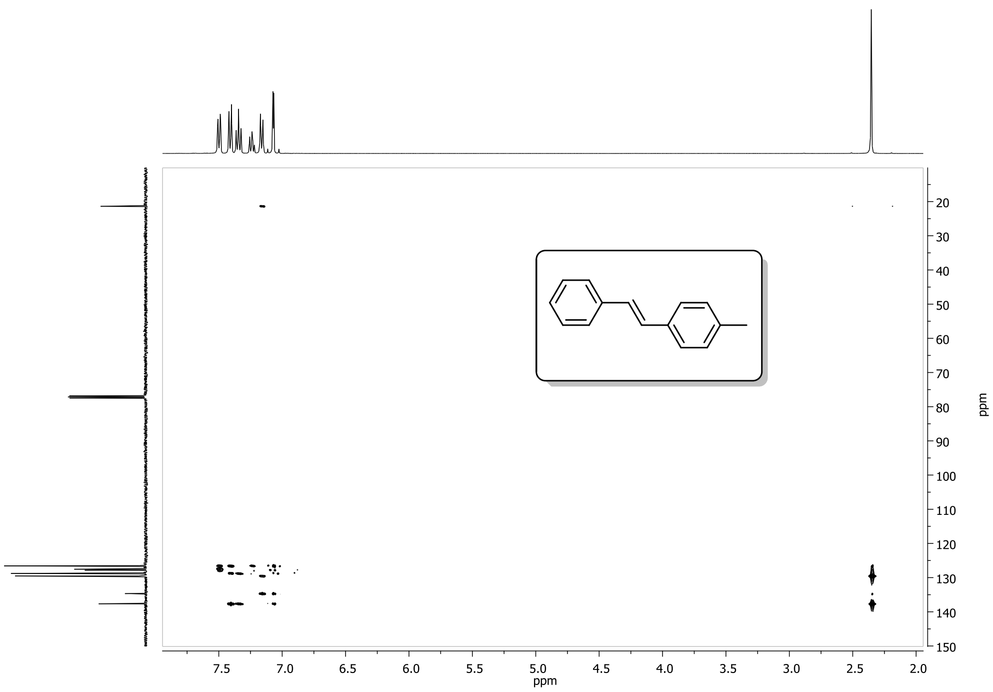

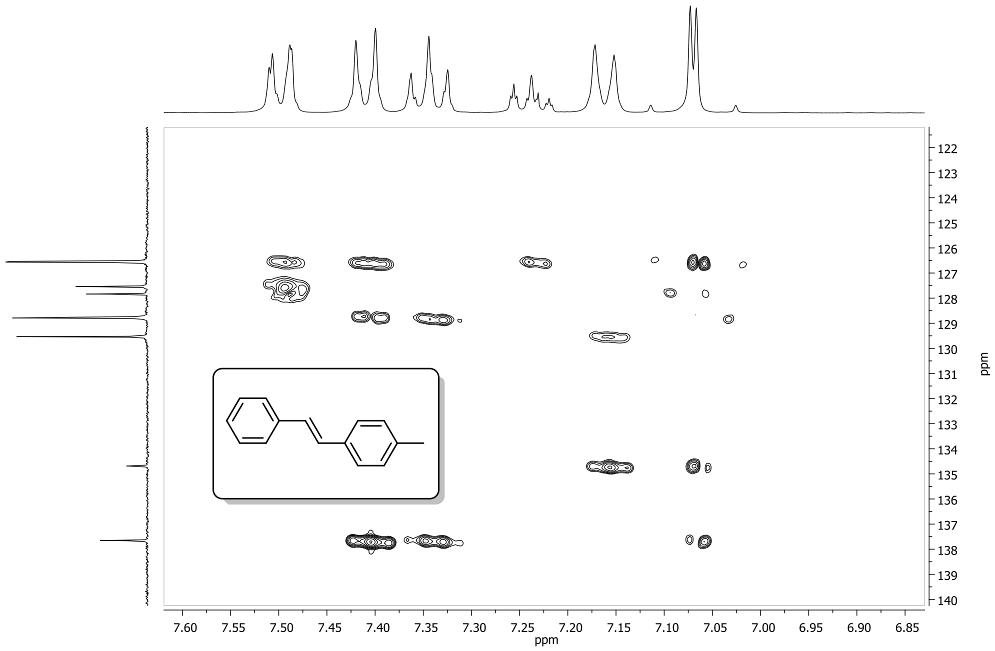

^{1}H NMR (400 MHz, $CDCl_3$) *(E)*-1-methyl-2-styrylbenzene (9)

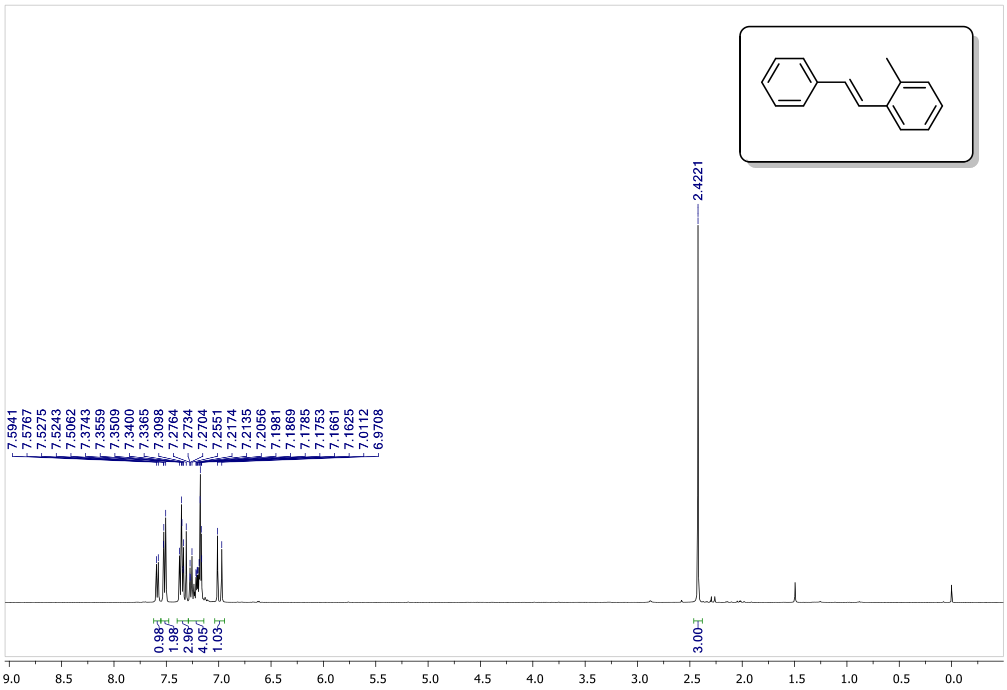

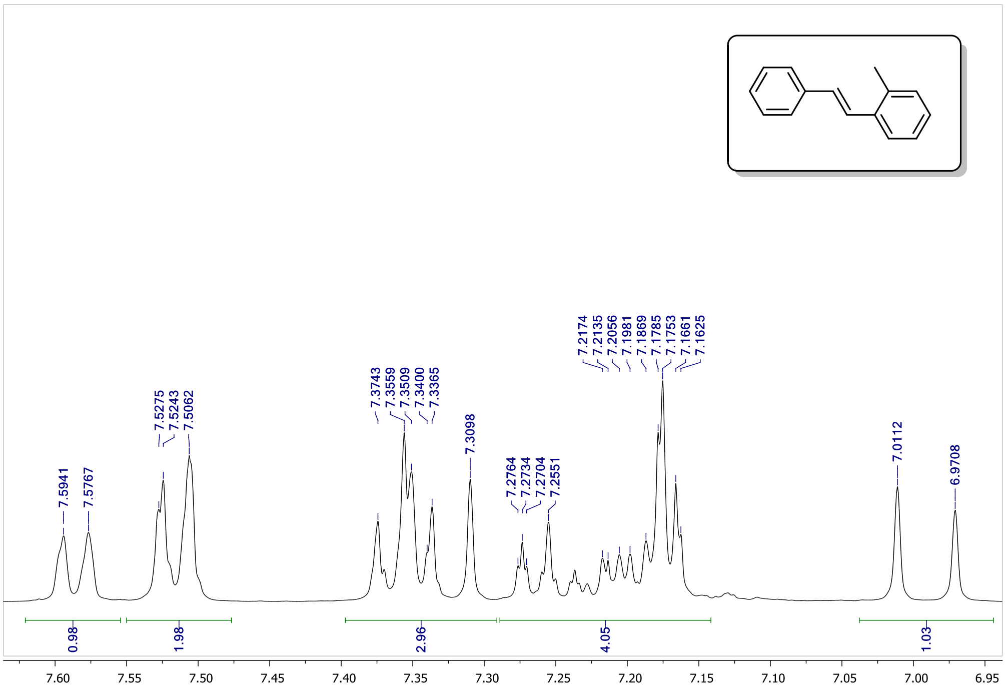

^{13}C NMR (101 MHz, $CDCl_3$) *(E)*-1-methyl-2-styrylbenzene (9)

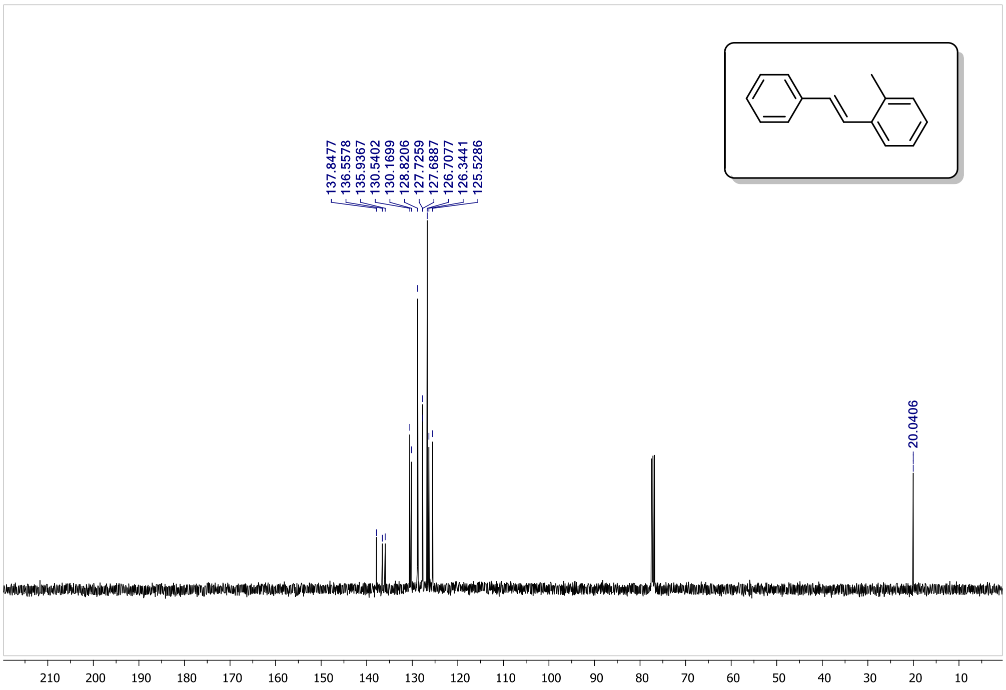

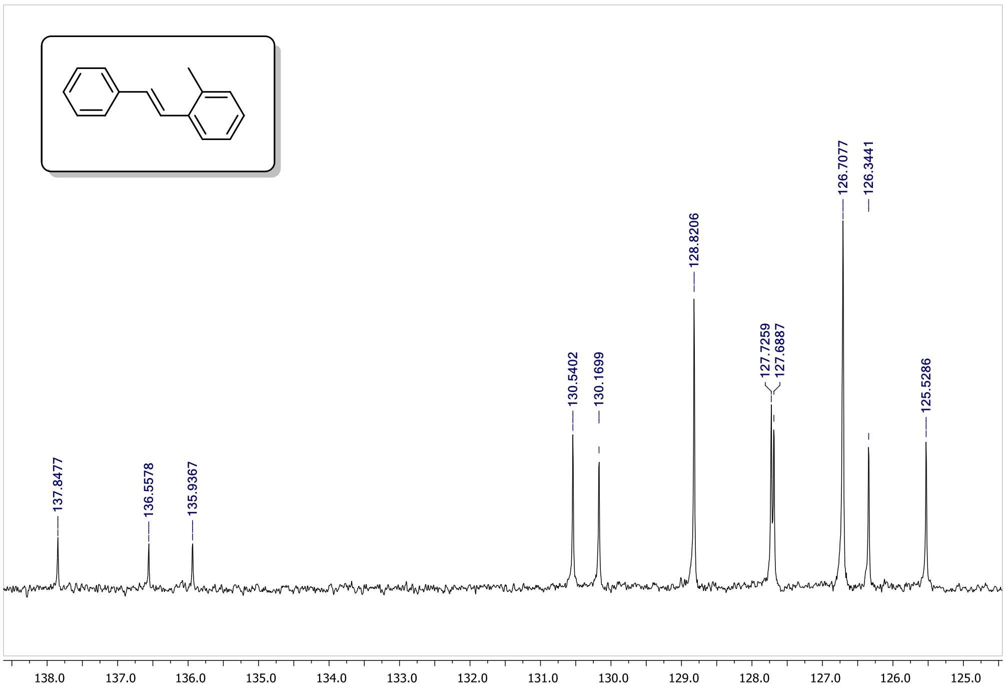

^{1}H-^{1}H COSY NMR (400 MHz, $CDCl_3$) *(E)*-1-methyl-2-styrylbenzene (9)

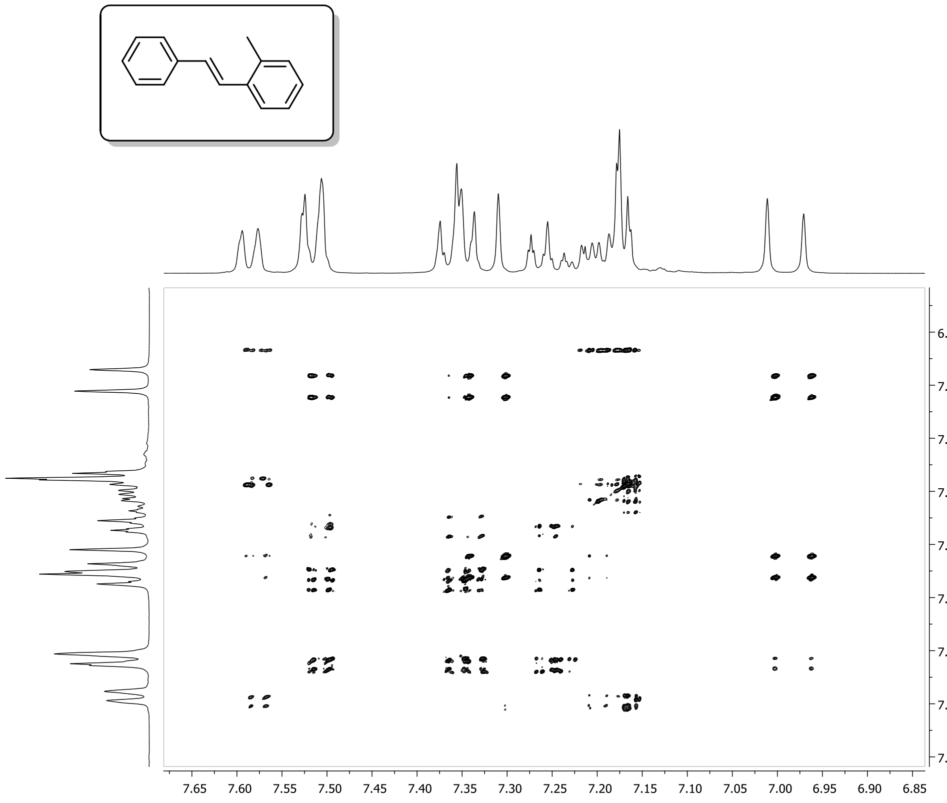

H-^{13}C HSQC NMR (400 MHz, $CDCl_3$) *(E)*-1-methyl-2-styrylbenzene (9)

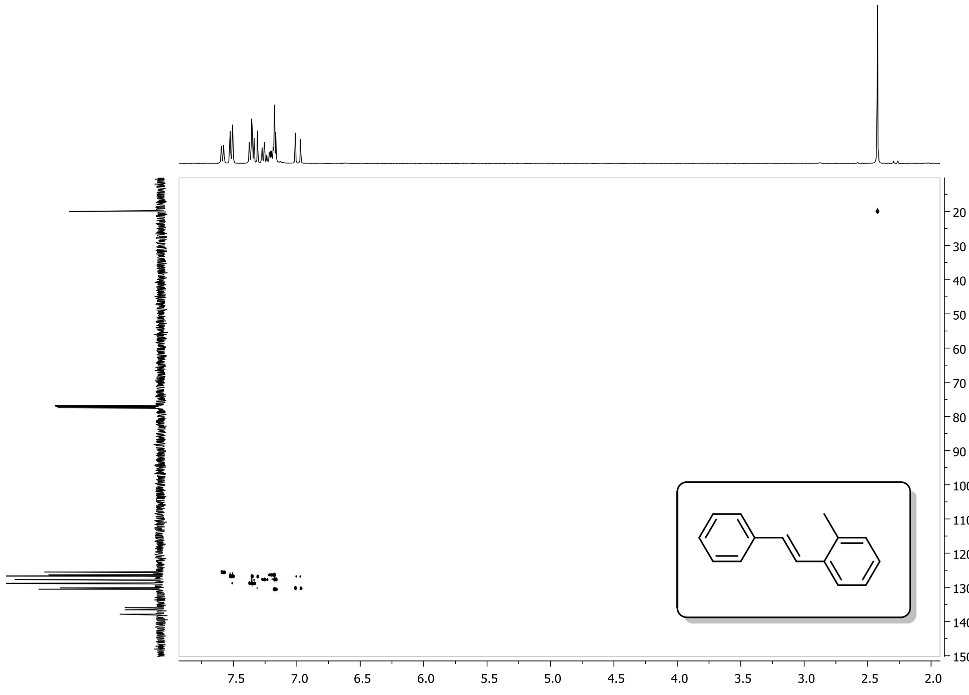

^{1}H-^{13}C HSQC NMR (400 MHz, $CDCl_3$) *(E)*-1-methyl-2-styrylbenzene (9)

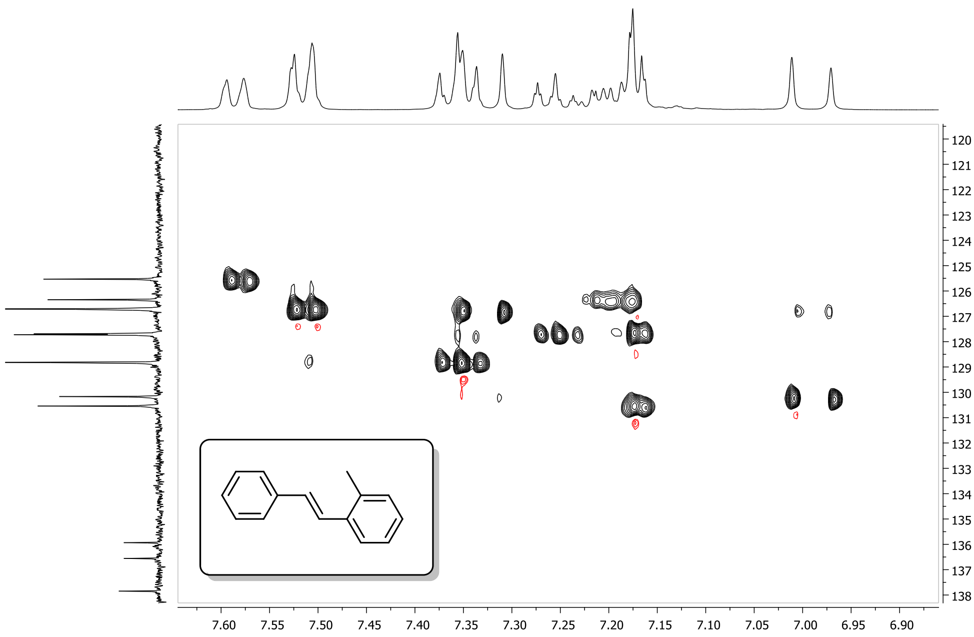

^{1}H-^{13}C HMBC NMR (400 MHz, $CDCl_3$) *(E)*-1-methyl-2-styrylbenzene (9)

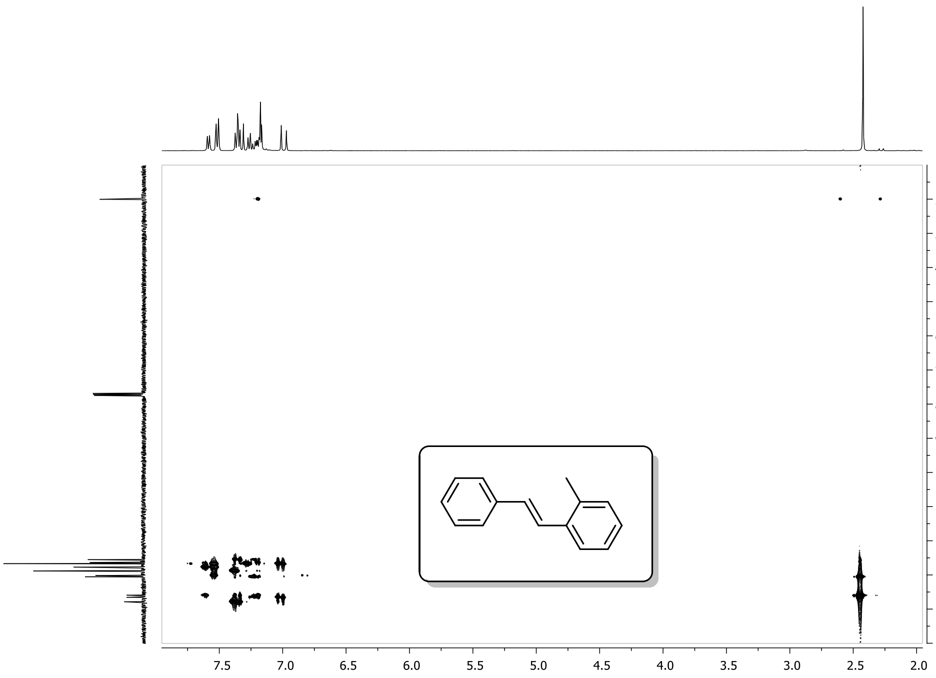

^{1}H-^{13}C HMBC NMR (400 MHz, CDCl$_3$) *(E)*-1-methyl-2-styrylbenzene (9)

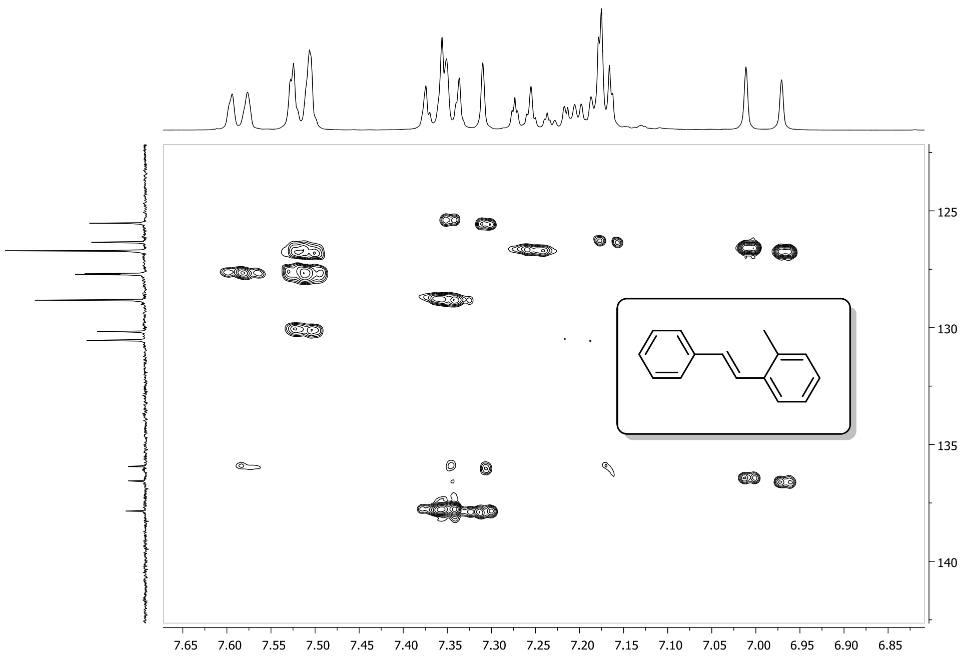

^{1}H NMR (400 MHz, Acetone-d_6) *(E)*-4-styrylphenol (10)

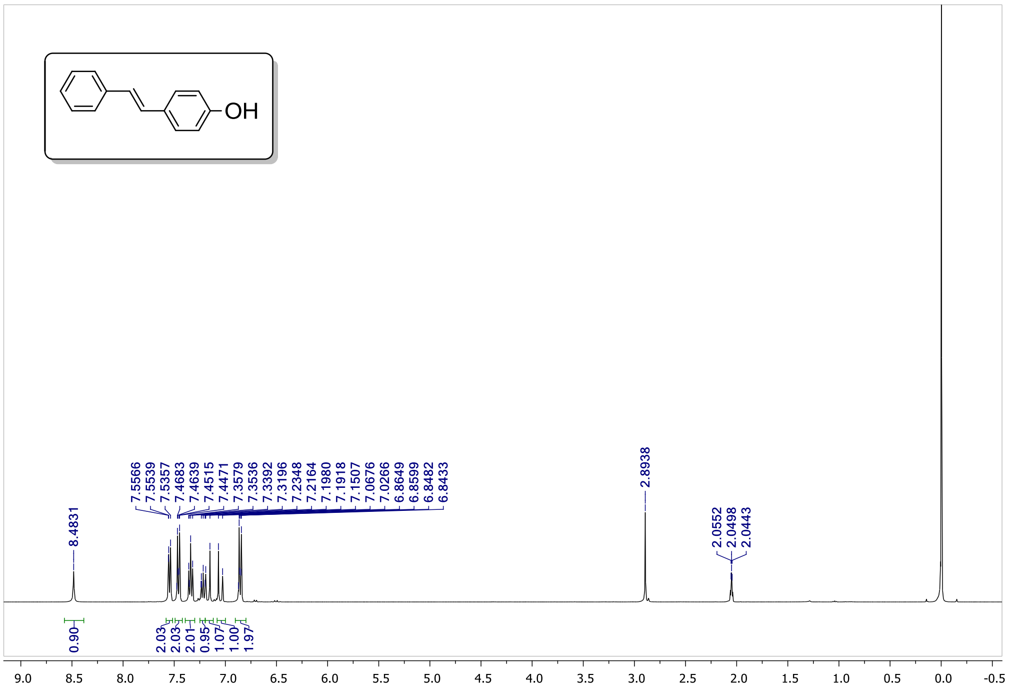

^{1}H NMR (400 MHz, Acetone-d_6) *(E)*-4-styrylphenol (10)

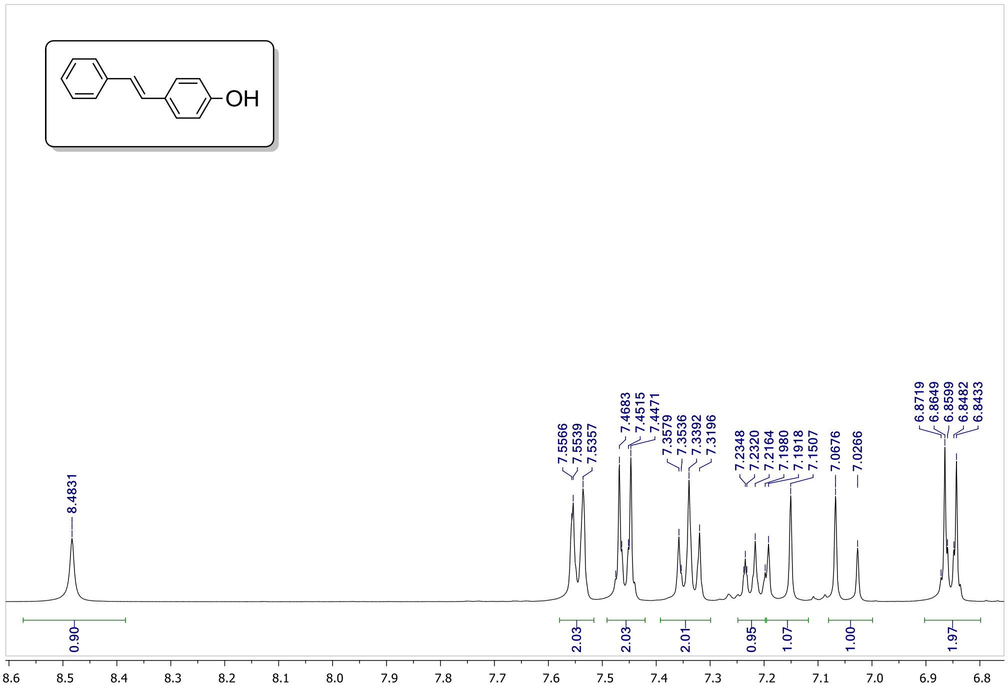

^{13}C NMR (101 MHz, Acetone-*d_6*) *(E)*-4-styrylphenol (10)

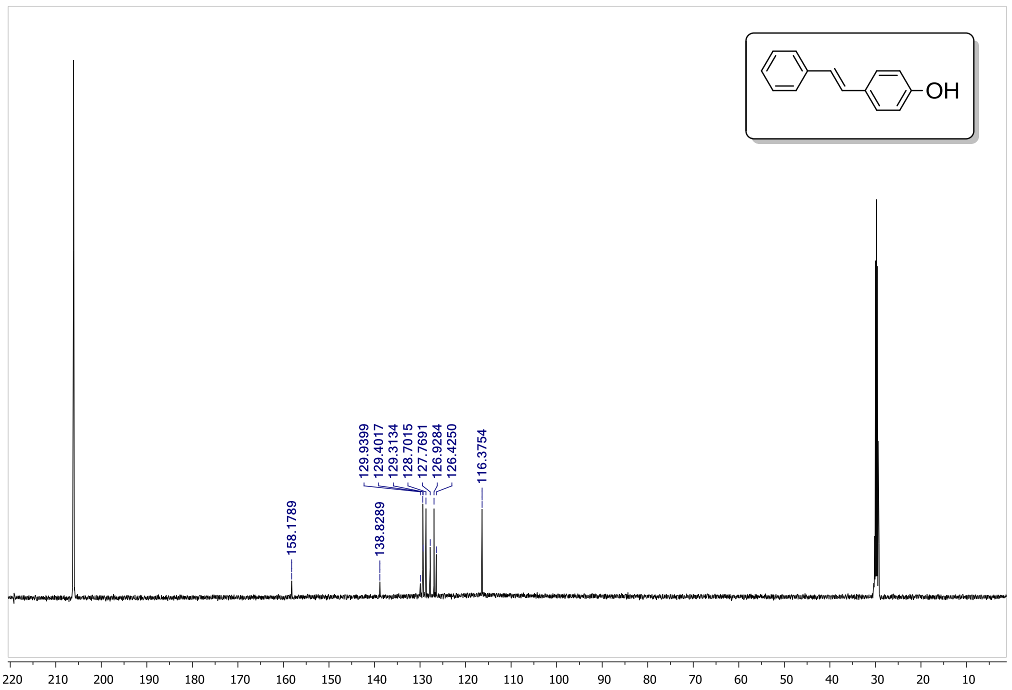

^{13}C NMR (101 MHz, Acetone-*d_6*) *(E)*-4-styrylphenol (10)

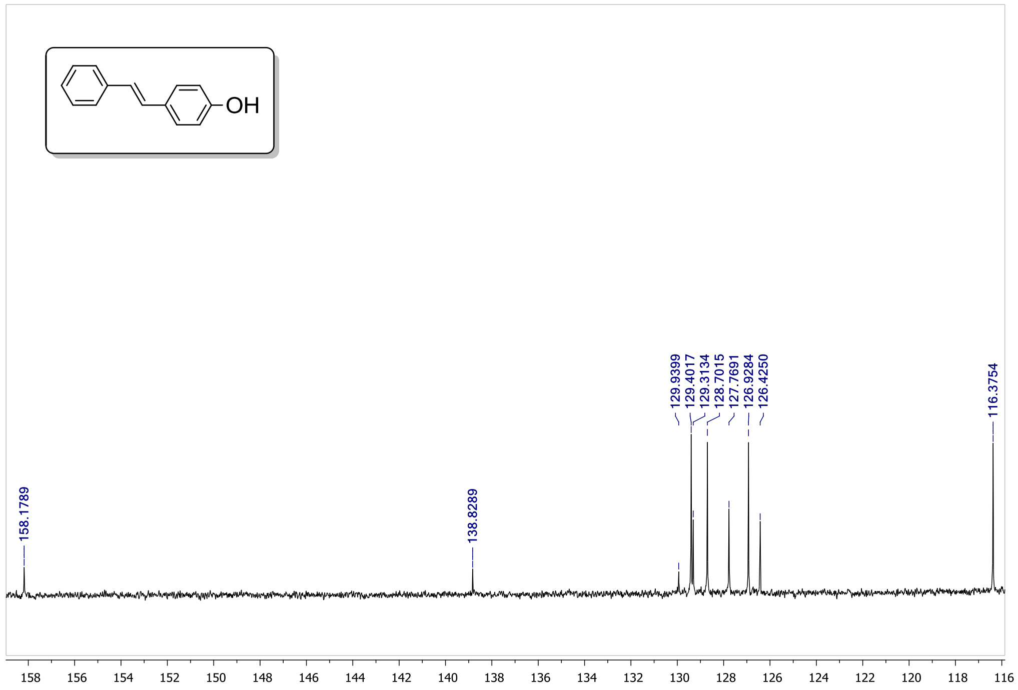

^{1}H-^{1}H COSY NMR (400 MHz, Acetone-*d_6*) *(E)*-4-styrylphenol (10)

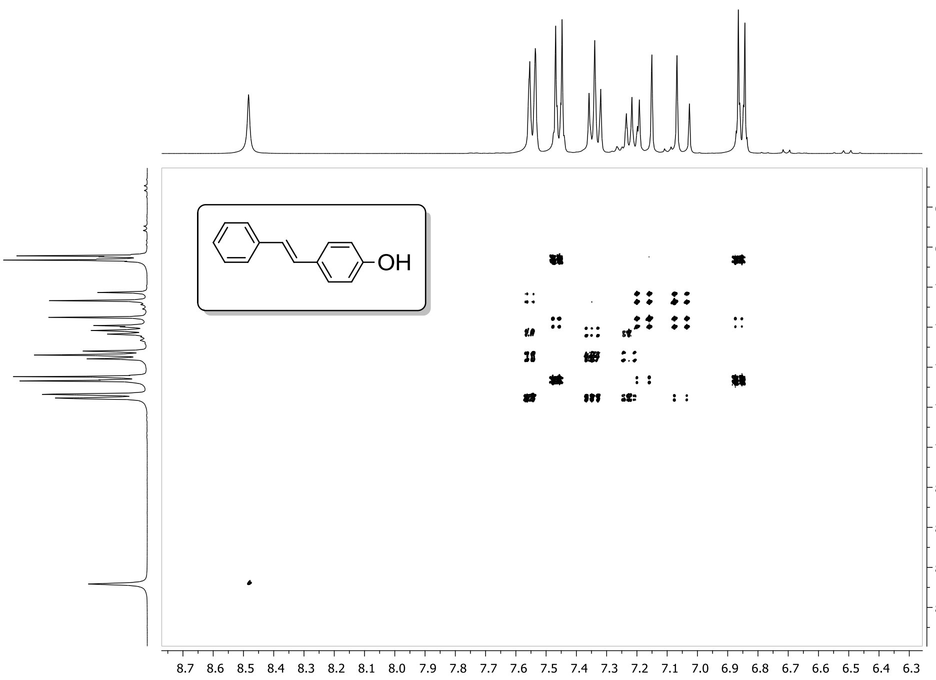

^{1}H-^{13}C HSQC NMR (400 MHz, Acetone-*d_6*) *(E)*-4-styrylphenol (10)

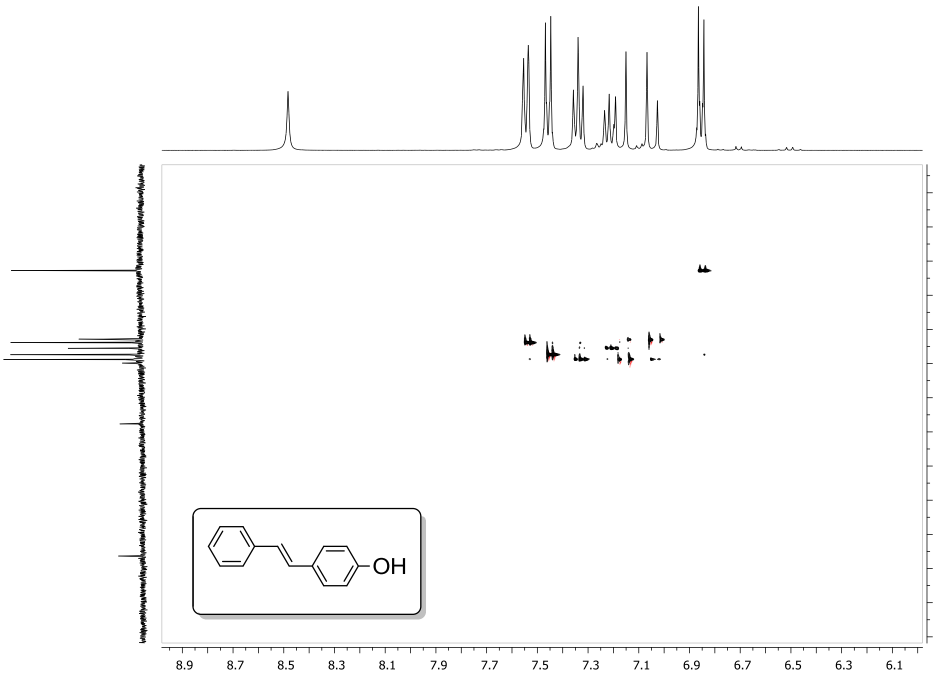

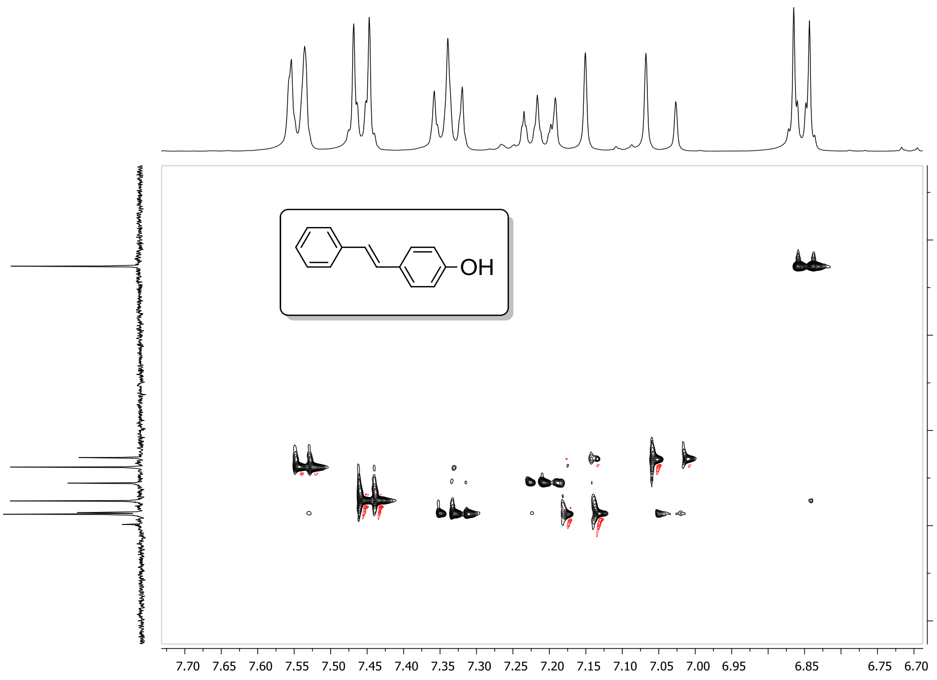

^1H-^{13}C **HMBC NMR (400 MHz, Acetone-*d_6*) *(E)*-4-styrylphenol (10)**

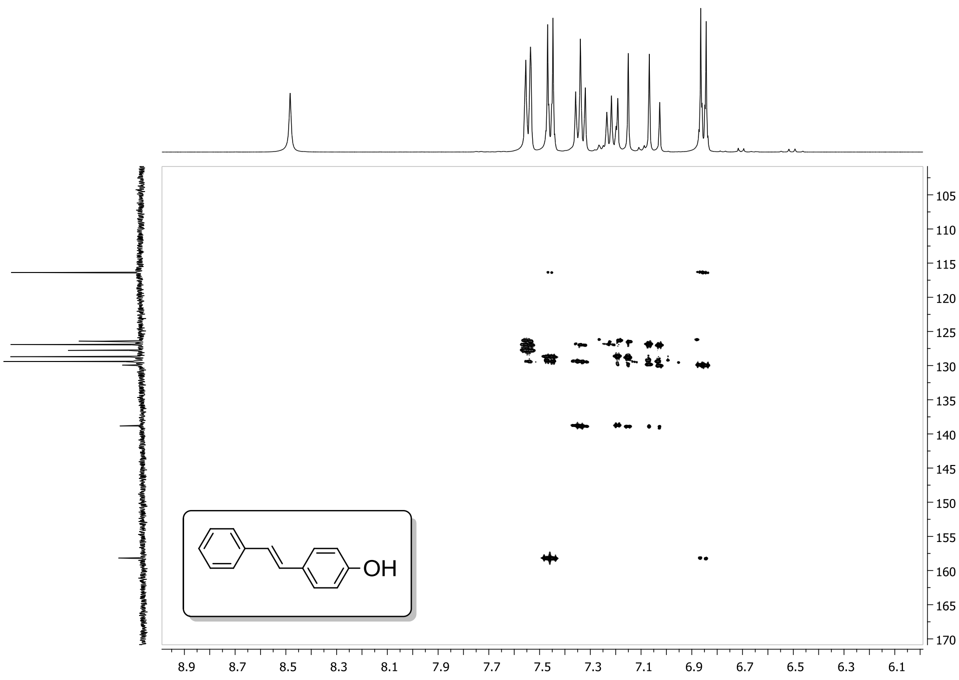

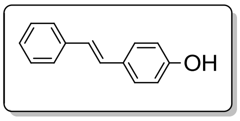
OH

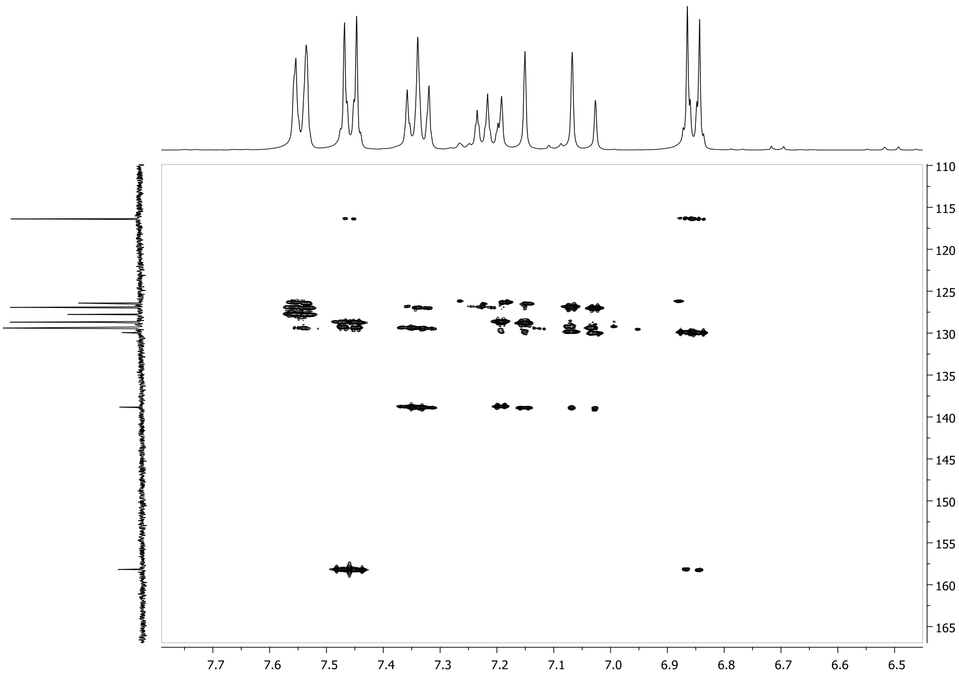
110
115
120
125
130
135
140
145
150
155
160
165
7.7
7.6
7.5
7.4
7.3
7.2
7.1
7.0
6.9
6.8
6.7
6.6
6.5

^{1}H NMR (400 MHz, $CDCl_3$) *(E)*-1-(4-(2-(pyridin-4-yl)vinyl)phenyl)ethanone (11)

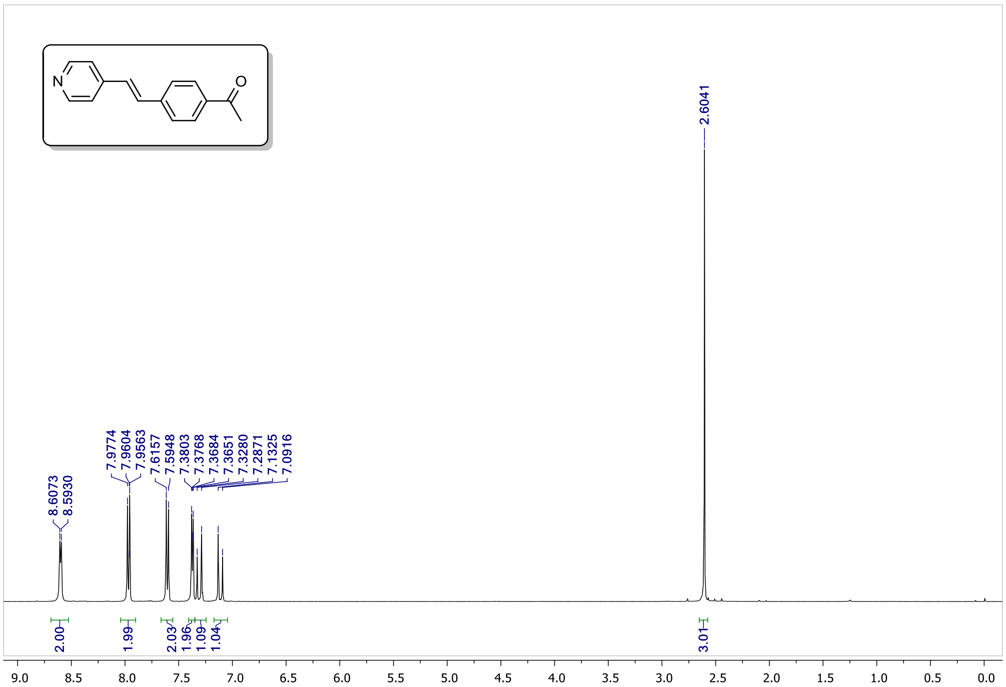

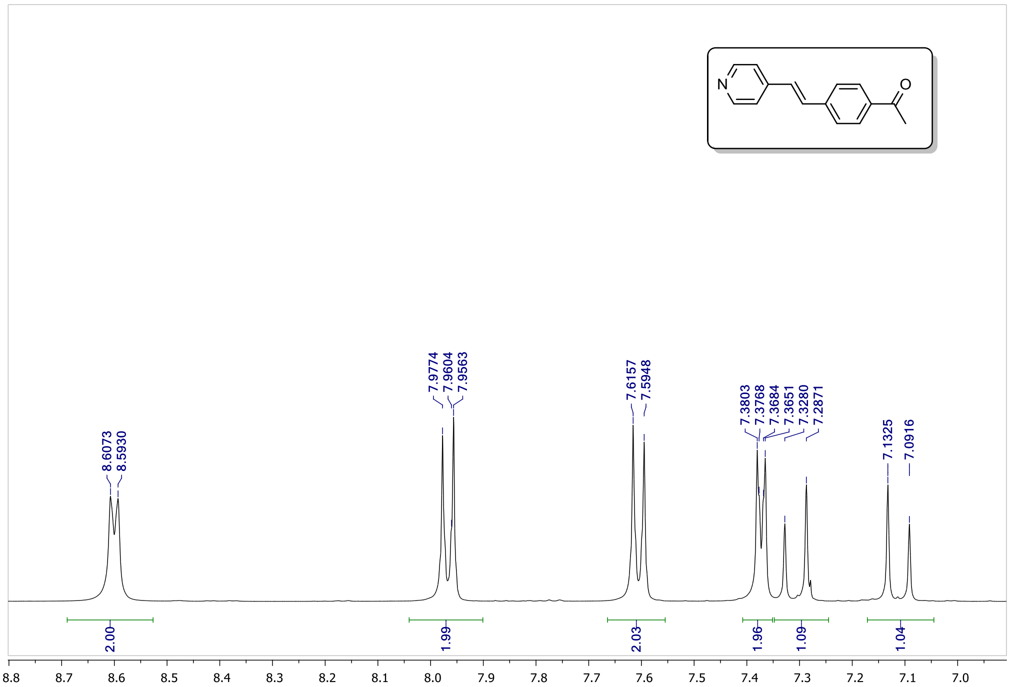

^{13}C NMR (101 MHz, $CDCl_3$) *(E)*-1-(4-(2-(pyridin-4-yl)vinyl)phenyl)ethanone (11)

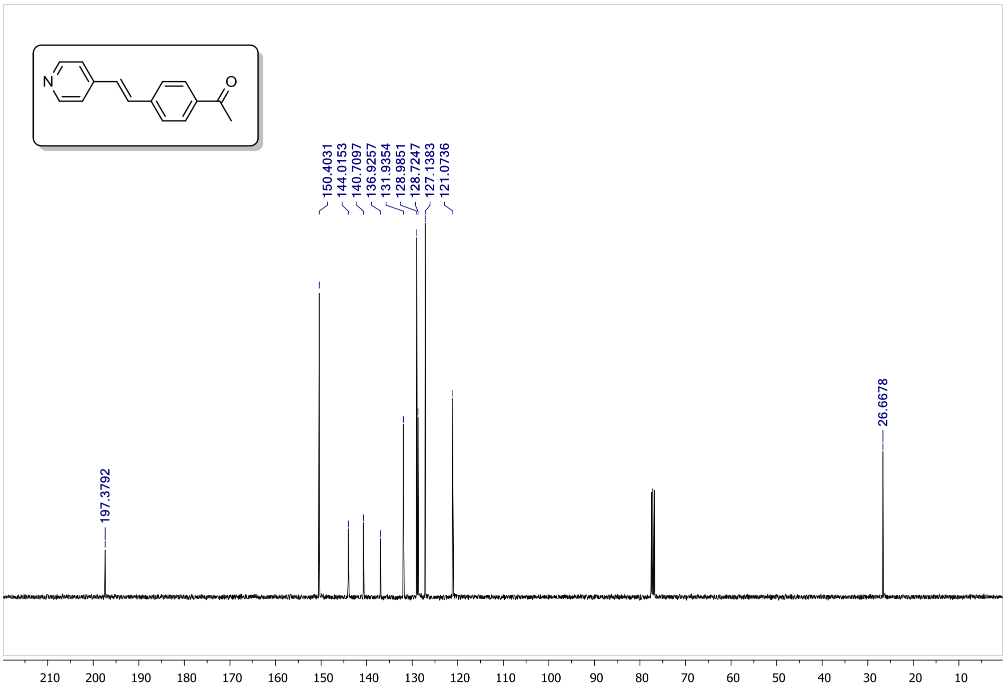

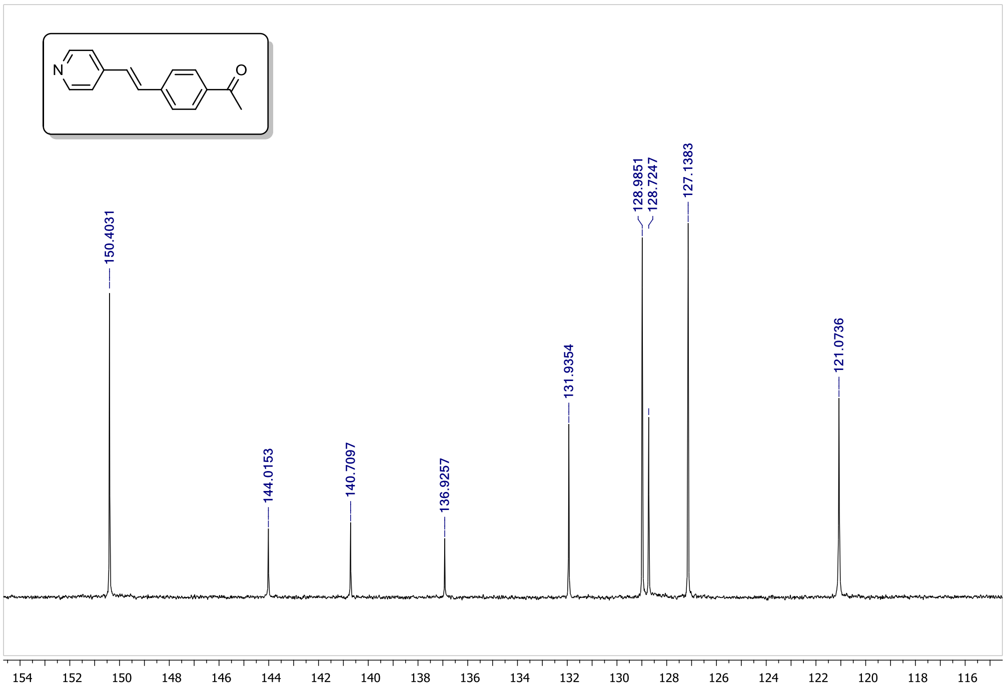

^{1}H-^{1}H COSY NMR (400 MHz, $CDCl_3$) *(E)*-1-(4-(2-(pyridin-4-yl)vinyl)phenyl)ethanone (11)

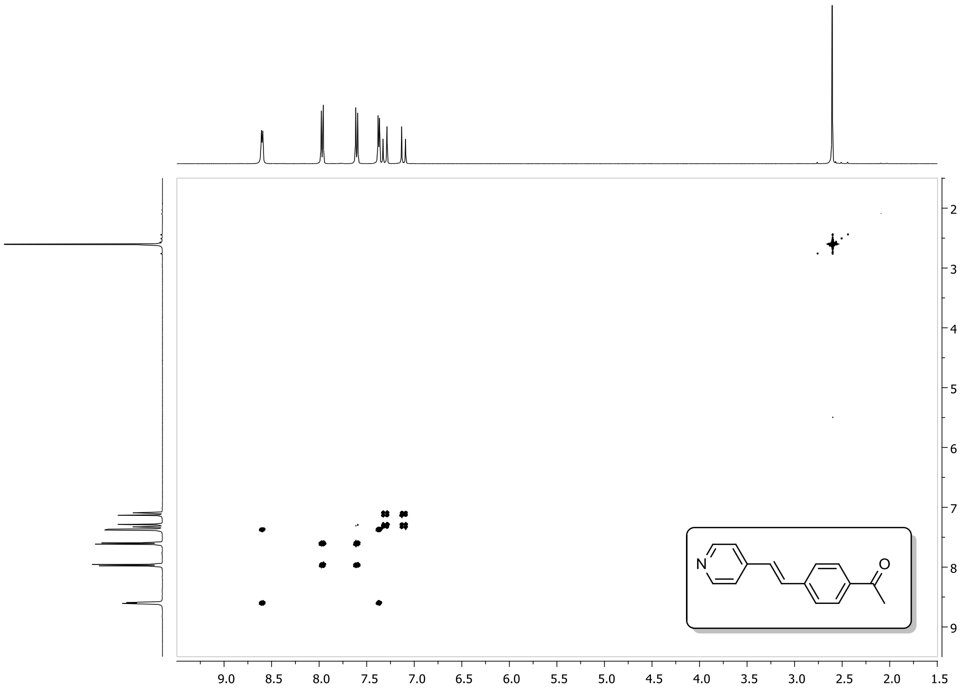

^{1}H-^{13}C HSQC NMR (400 MHz, $CDCl_3$) *(E)*-1-(4-(2-(pyridin-4-yl)vinyl)phenyl)ethanone (11)

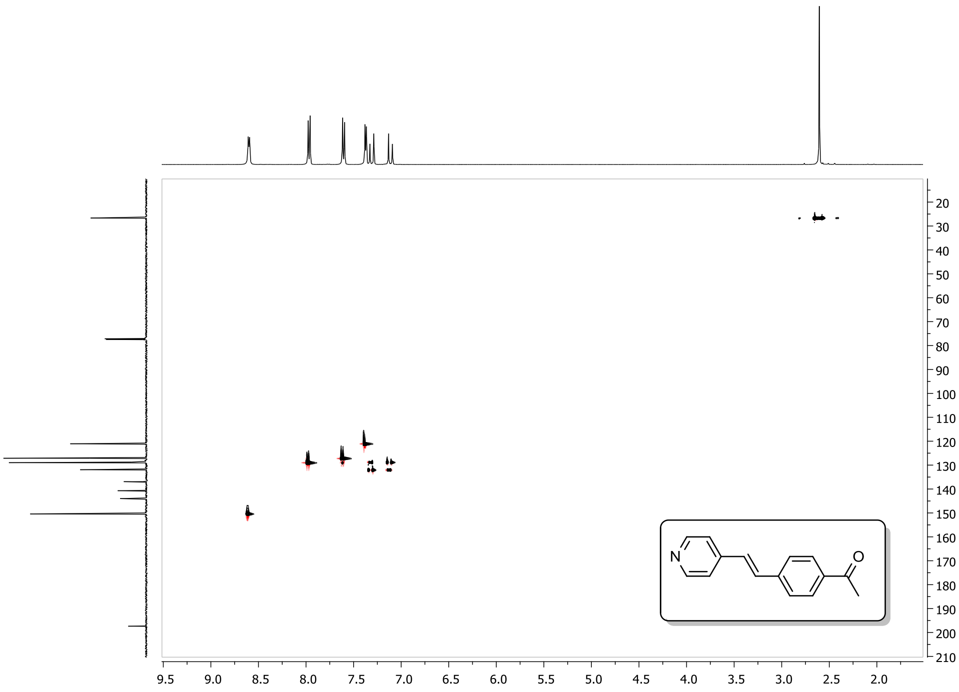

^{1}H-^{13}C HSQC NMR (400 MHz, $CDCl_3$) *(E)*-1-(4-(2-(pyridin-4-yl)vinyl)phenyl)ethanone (11)

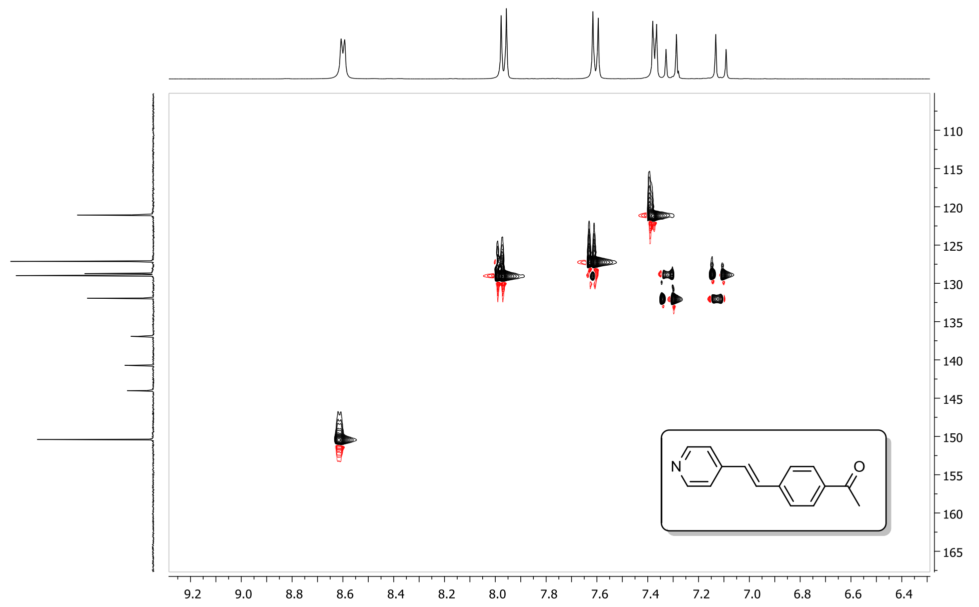

^{1}H-^{13}C HMBC NMR (400 MHz, $CDCl_3$) *(E)*-1-(4-(2-(pyridin-4-yl)vinyl)phenyl)ethanone (11)

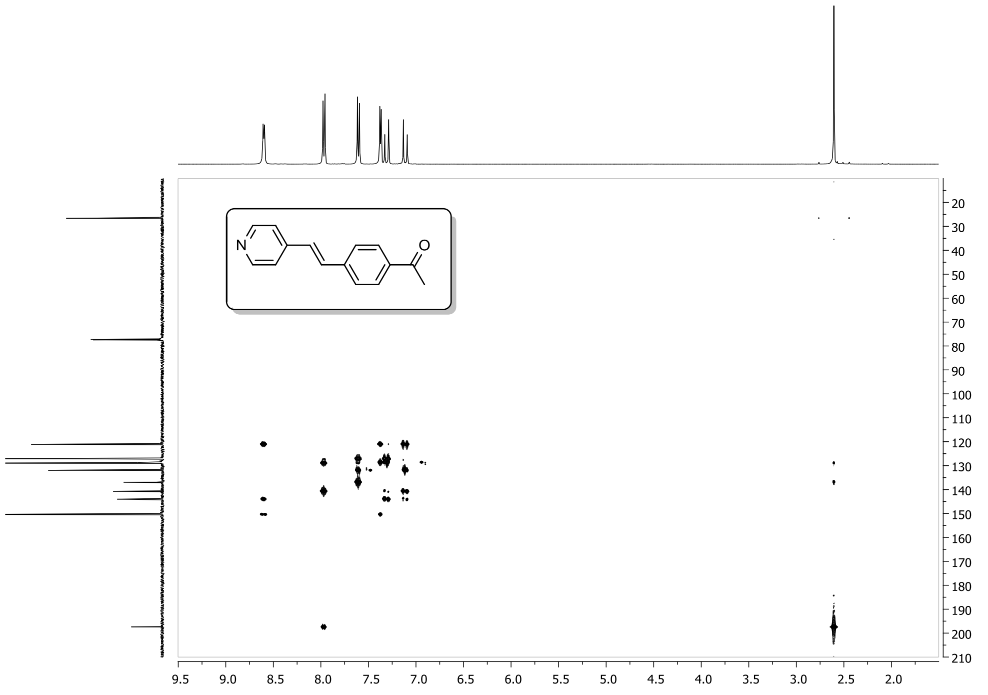

^{1}H-^{13}C HMBC NMR (400 MHz, CDCl$_3$) *(E)*-1-(4-(2-(pyridin-4-yl)vinyl)phenyl)ethanone (11)

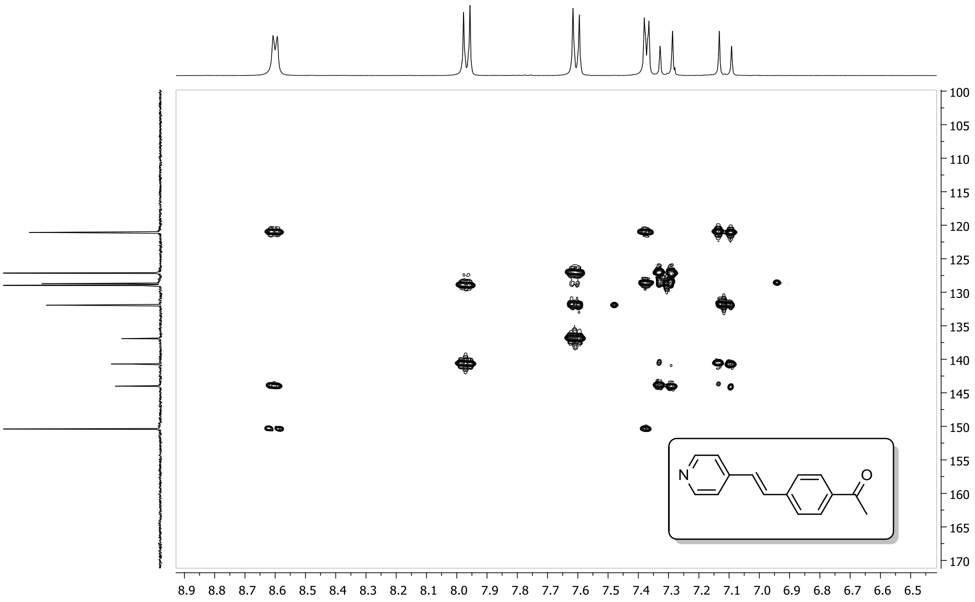

^{1}H NMR (400 MHz, CDCl$_3$) *(E)*-phenyl(4-(2-(pyridin-4-yl)vinyl)phenyl)methanone (12)

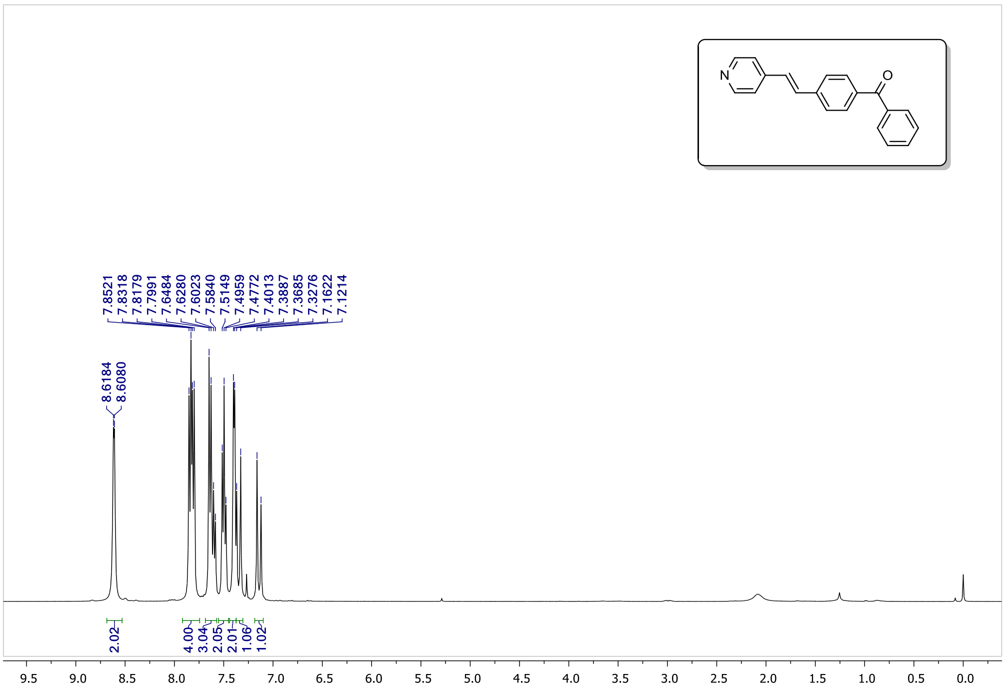

^{1}H NMR (400 MHz, CDCl$_3$) *(E)*-phenyl(4-(2-(pyridin-4-yl)vinyl)phenyl)methanone (12)

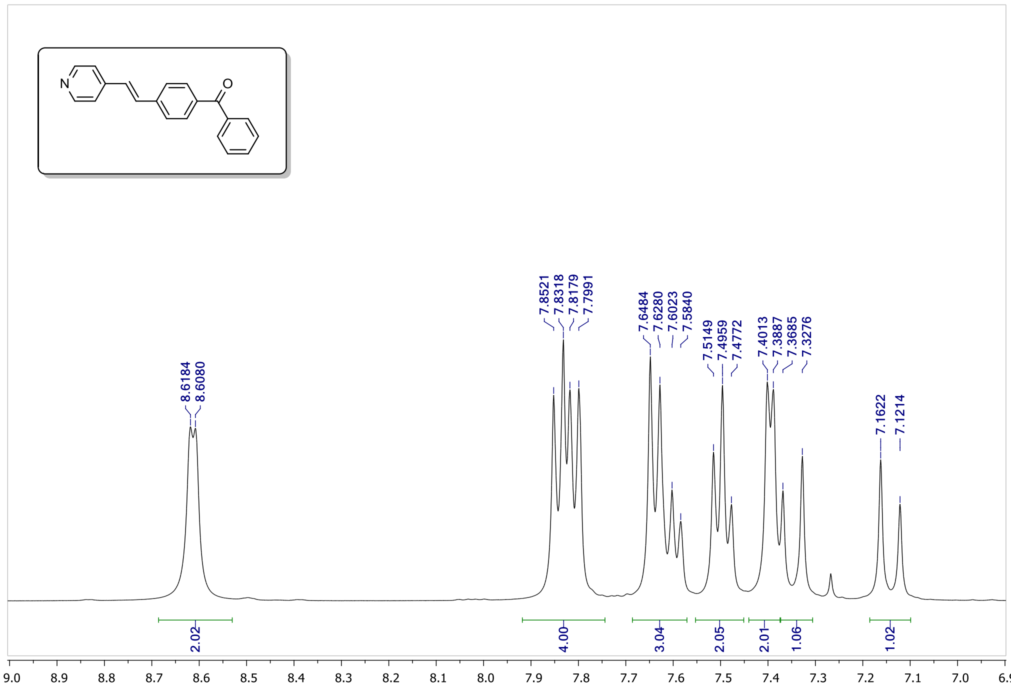

^{13}C NMR (101 MHz, CDCl$_3$) *(E)*-phenyl(4-(2-(pyridin-4-yl)vinyl)phenyl)methanone (12)

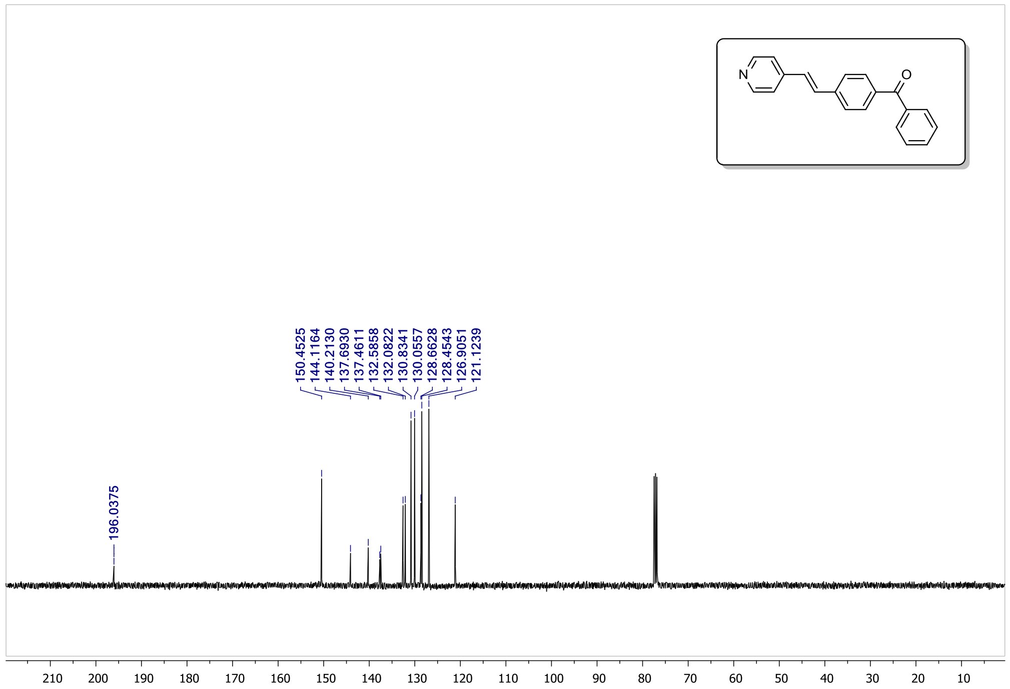

^{13}C NMR (101 MHz, $CDCl_3$) *(E)*-phenyl(4-(2-(pyridin-4-yl)vinyl)phenyl)methanone (12)

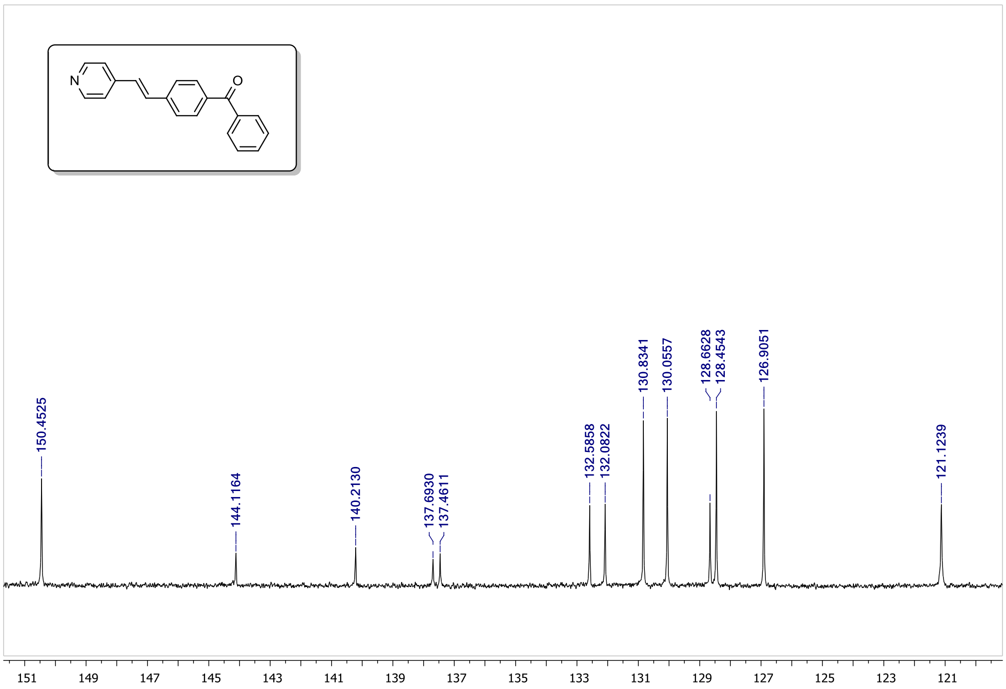

^{1}H-^{1}H COSY NMR (400 MHz, $CDCl_3$) *(E)*-phenyl(4-(2-(pyridin-4-yl)vinyl)phenyl)methanone (12)

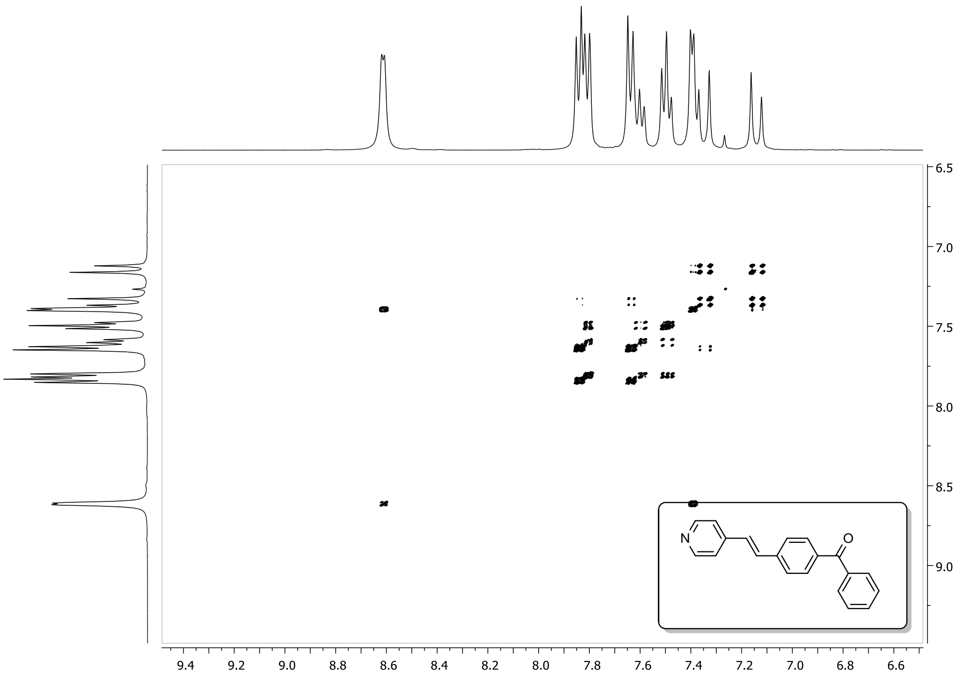

^{1}H-^{13}C HSQC NMR (400 MHz, $CDCl_3$) *(E)*-phenyl(4-(2-(pyridin-4-yl)vinyl)phenyl)methanone (12)

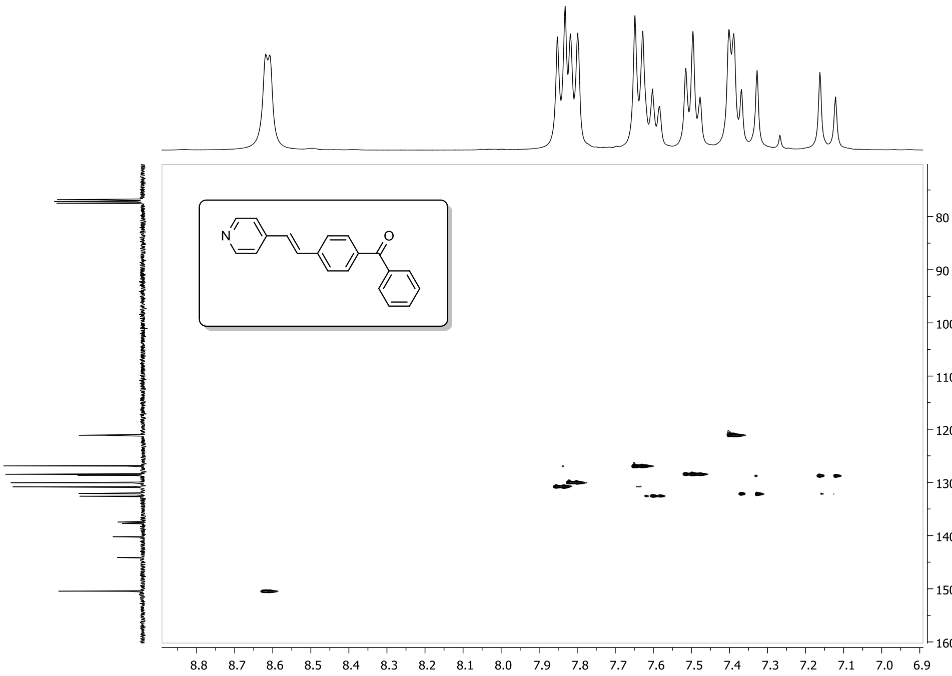

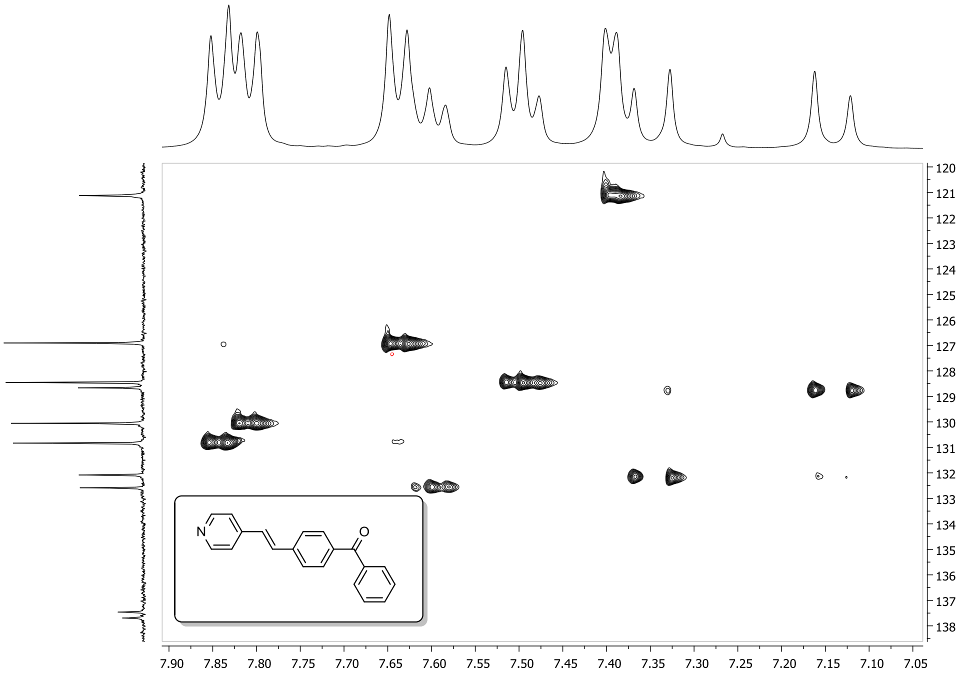

^{1}H-^{13}C HMBC NMR (400 MHz, $CDCl_3$) *(E)*-phenyl(4-(2-(pyridin-4-yl)vinyl)phenyl)methanone (12)

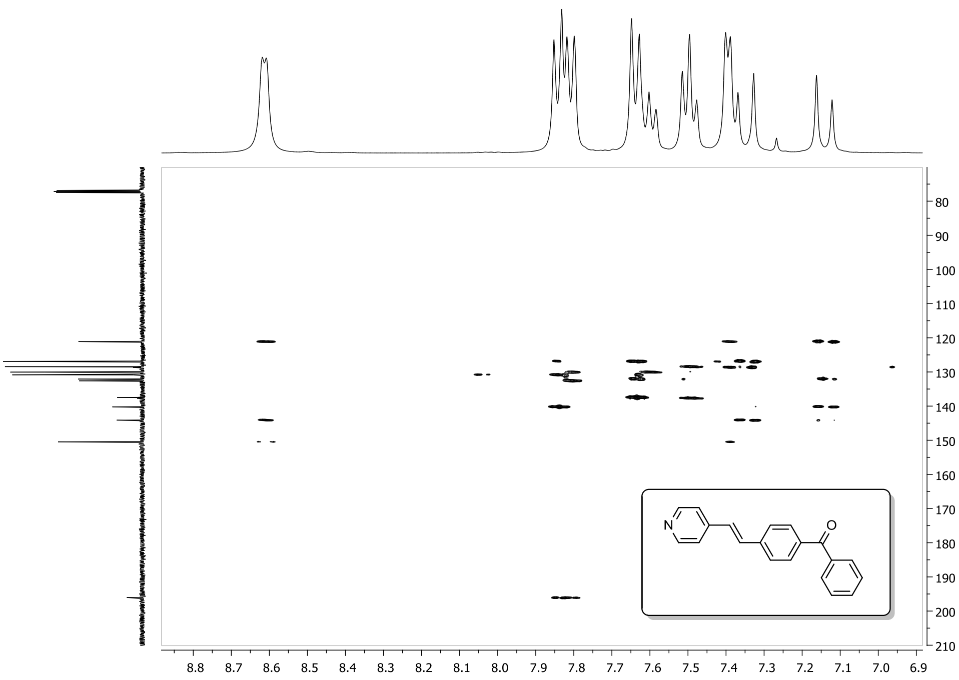

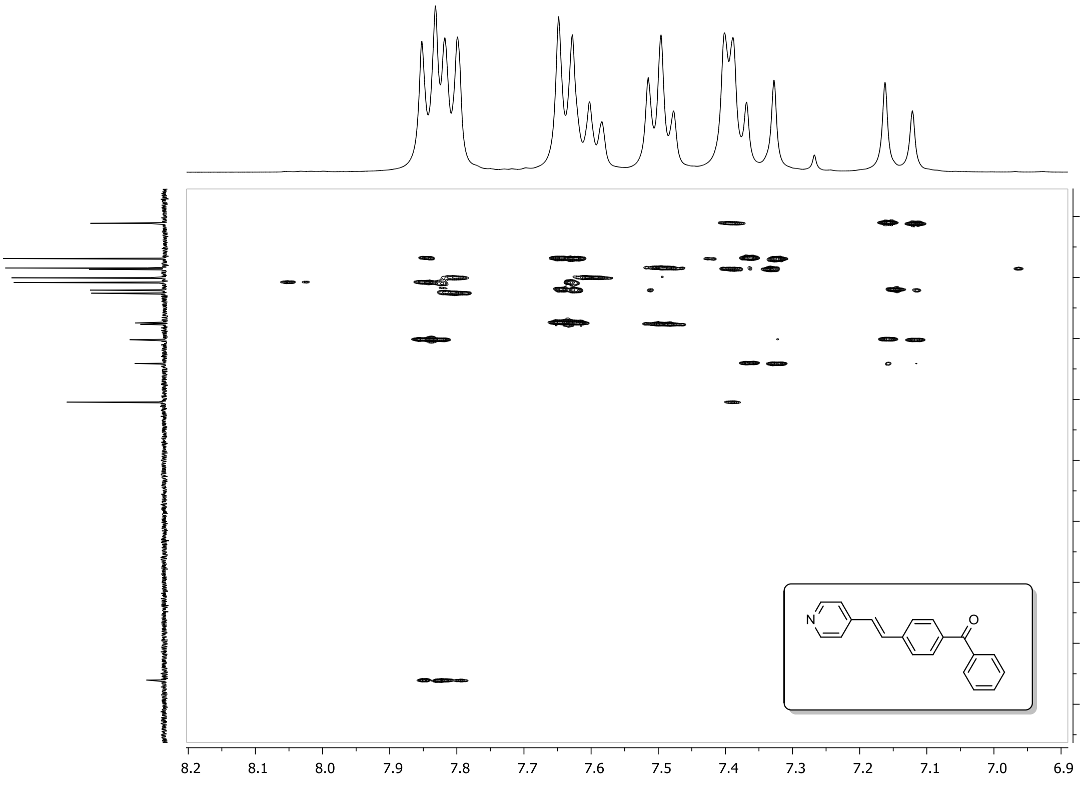

^{1}H NMR (400 MHz, $CDCl_3$) *(E)*-3-(2-(pyridin-4-yl)vinyl)quinoline (13)

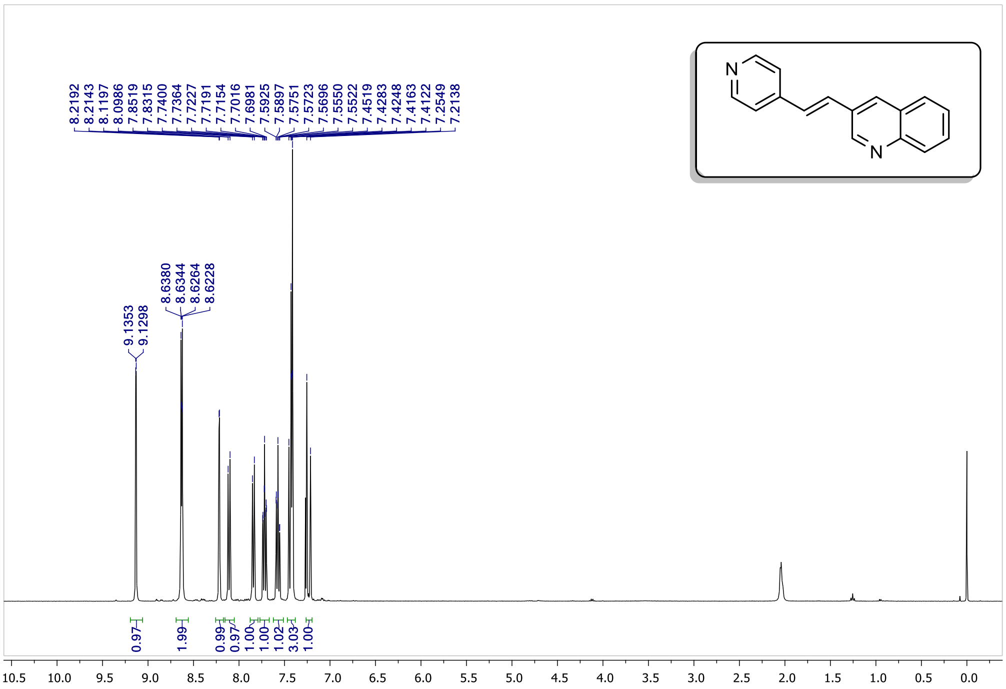

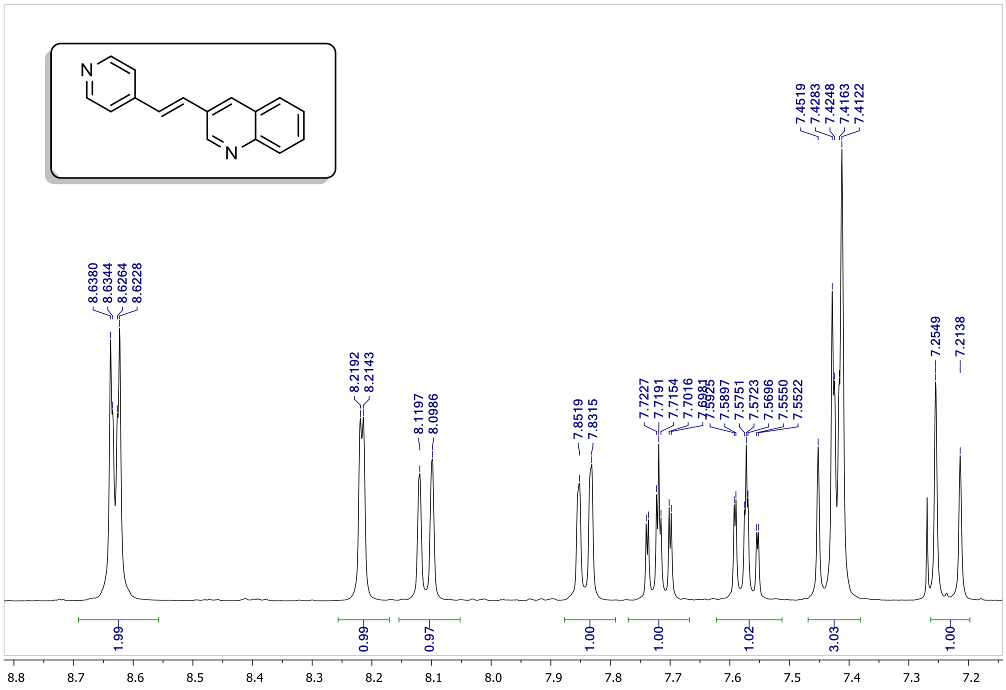

^{13}C NMR (101 MHz, $CDCl_3$) *(E)*-3-(2-(pyridin-4-yl)vinyl)quinoline (13)

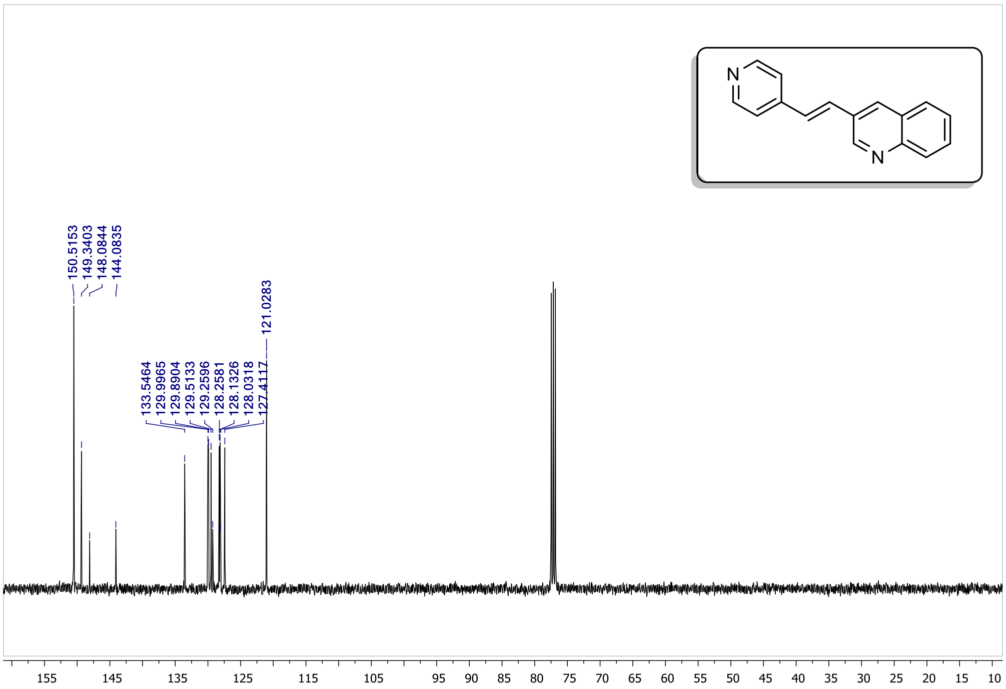

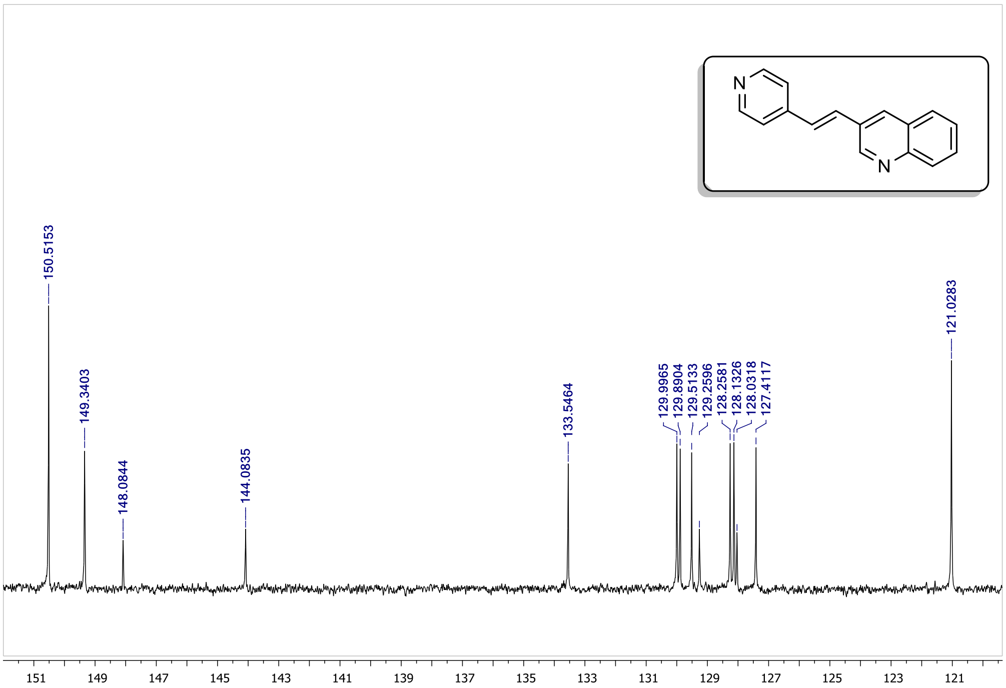

^{1}H-^{1}H COSY NMR (400 MHz, $CDCl_3$) *(E)*-3-(2-(pyridin-4-yl)vinyl)quinoline (13)

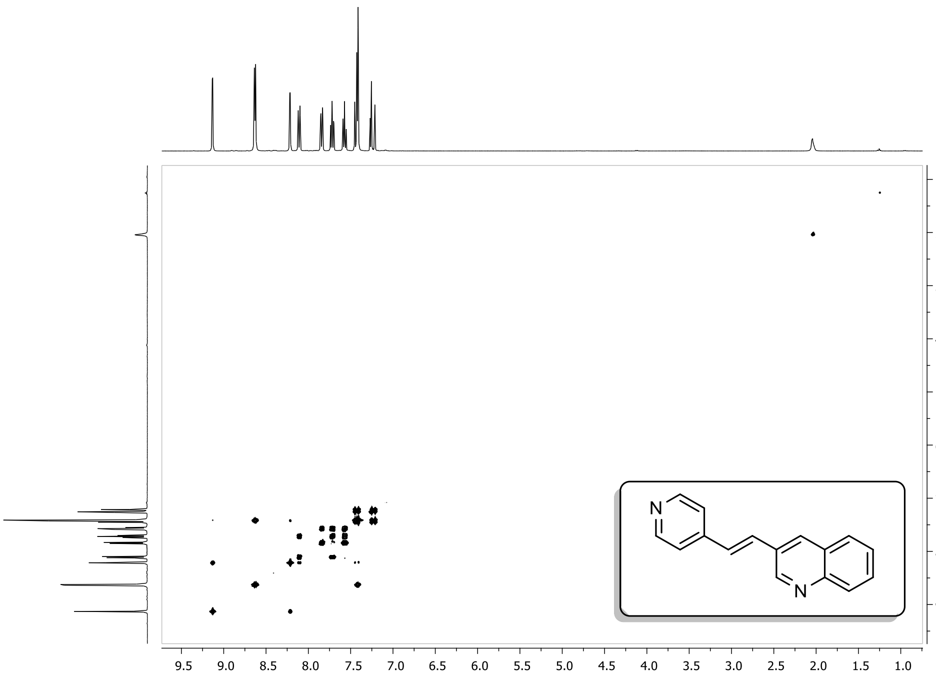

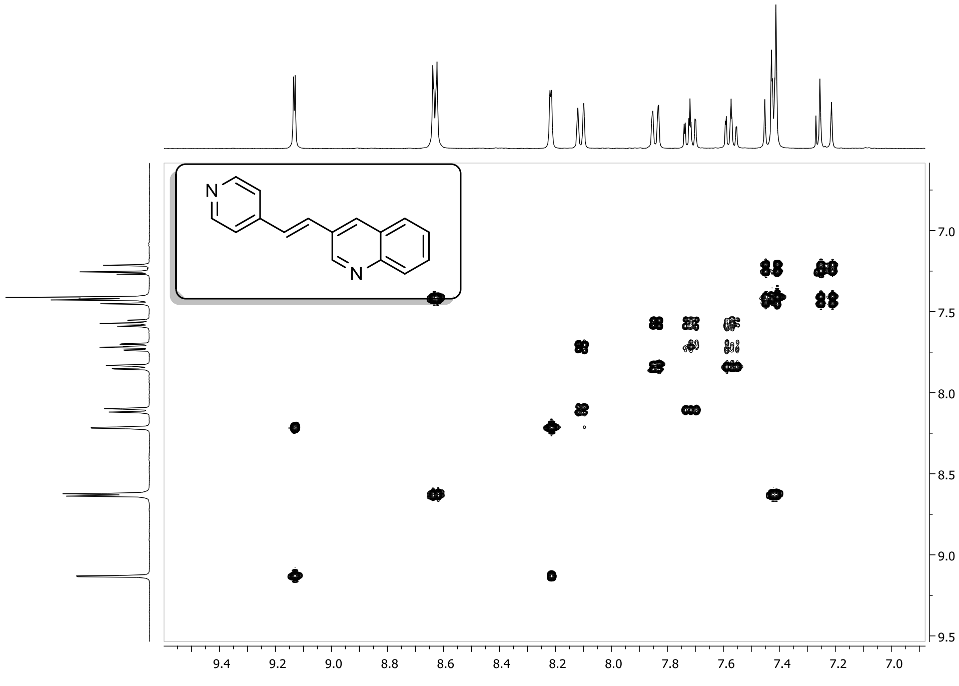

^{1}H-^{13}C HSQC NMR (400 MHz, $CDCl_3$) *(E)*-3-(2-(pyridin-4-yl)vinyl)quinoline (13)

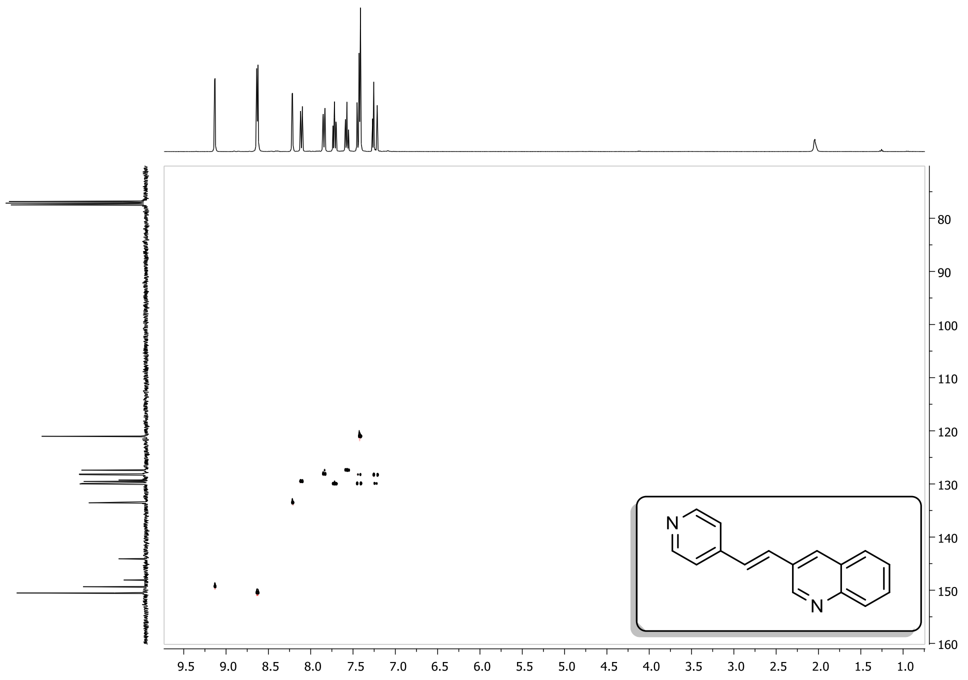

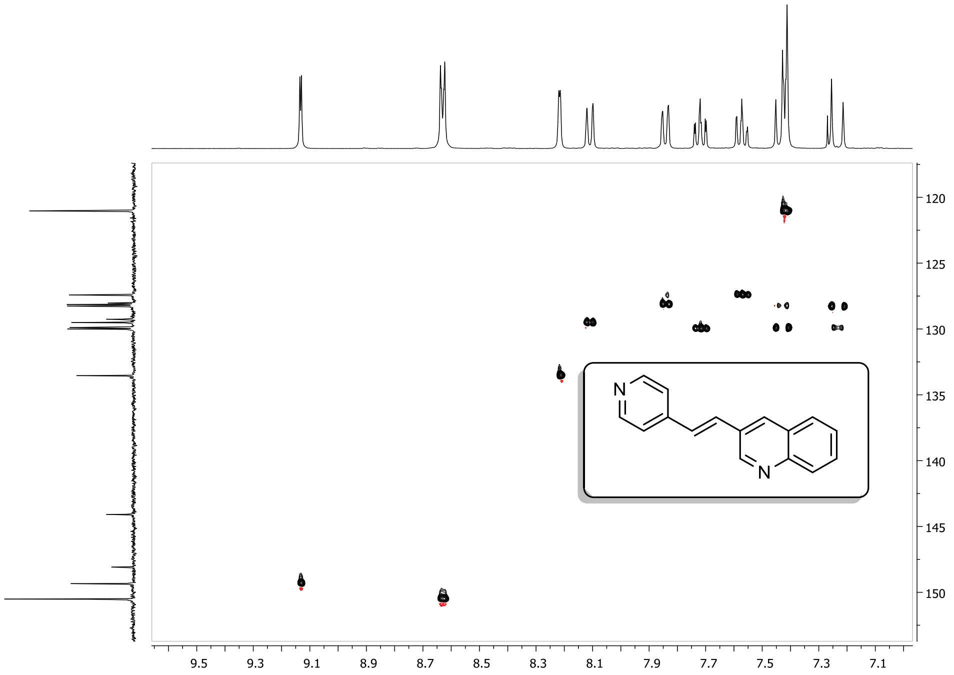

^{1}H-^{13}C HMBC NMR (400 MHz, $CDCl_3$) *(E)*-3-(2-(pyridin-4-yl)vinyl)quinoline (13)

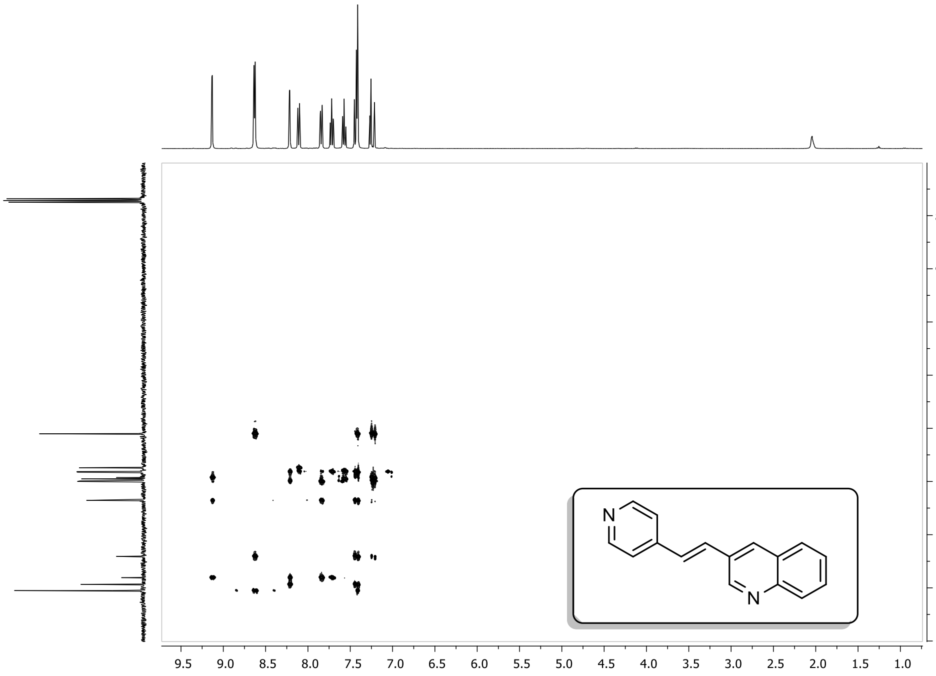

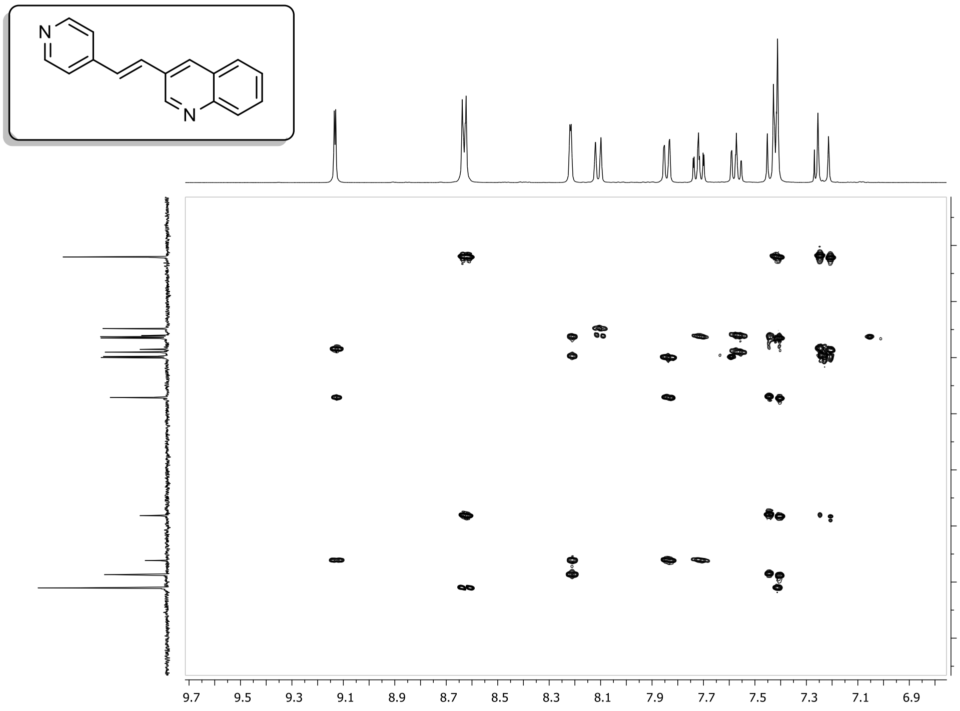

^{1}H NMR (400 MHz, CDCl$_3$) *(E)*-4-(3, 5-dimethoxystyryl)pyridine (14)

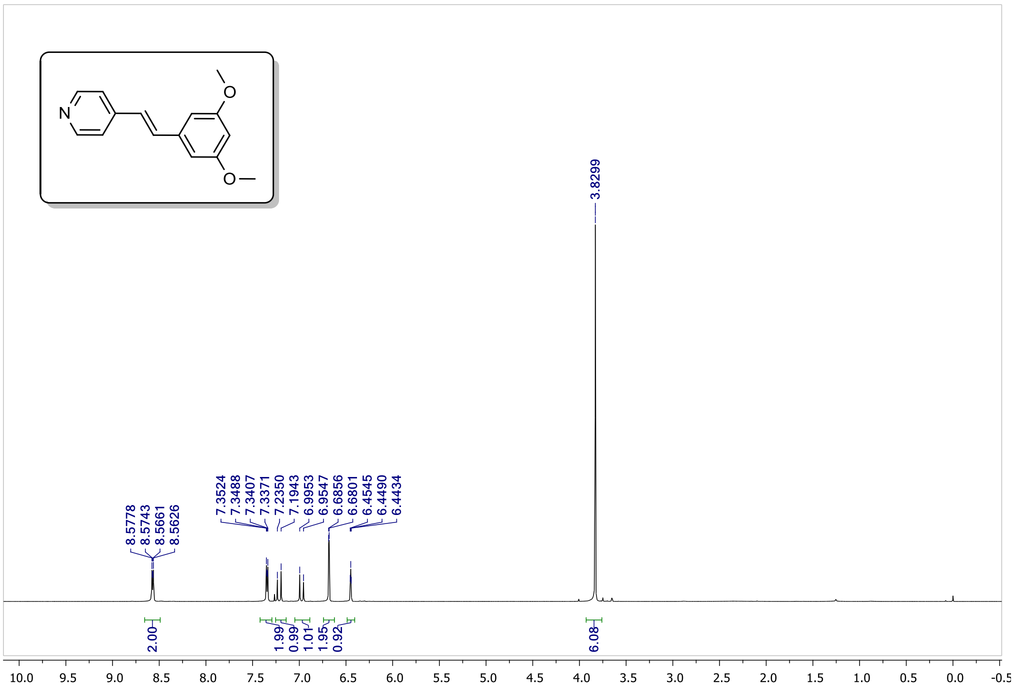

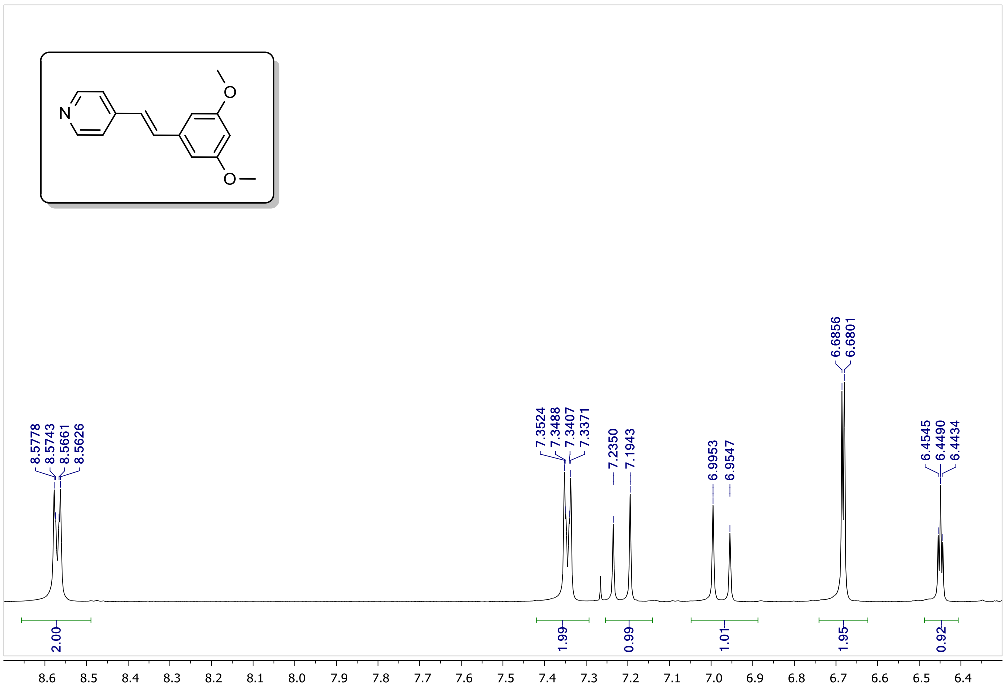

^{13}C NMR (101 MHz, CDCl$_3$) *(E)*-4-(3, 5-dimethoxystyryl)pyridine (14)

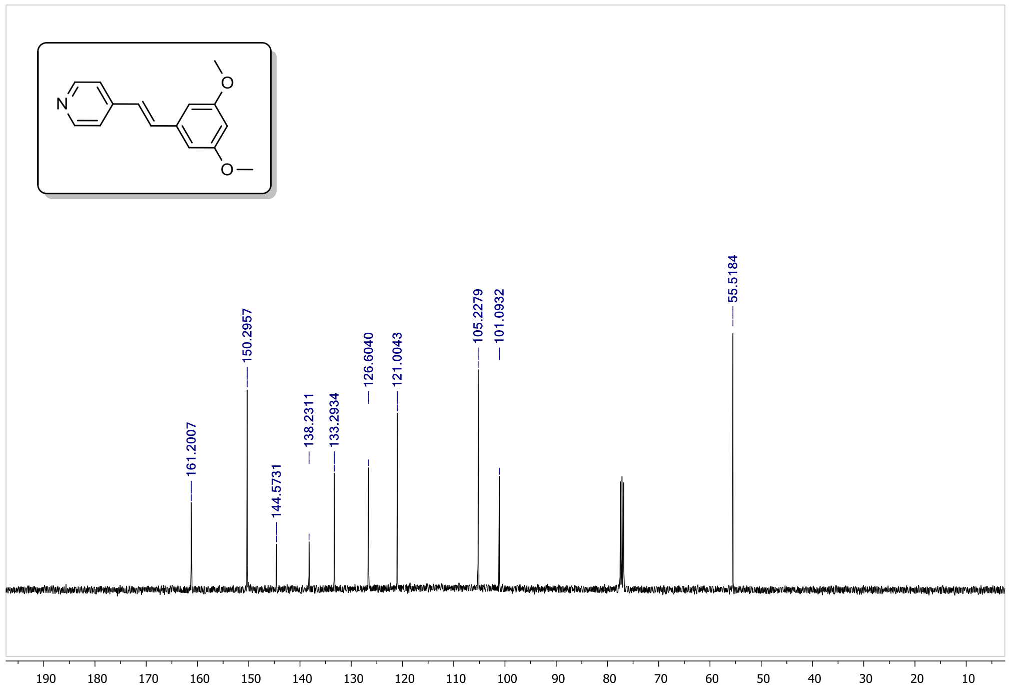

^{1}H-^{1}H COSY NMR (400 MHz, CDCl$_3$) *(E)*-4-(3, 5-dimethoxystyryl)pyridine (14)

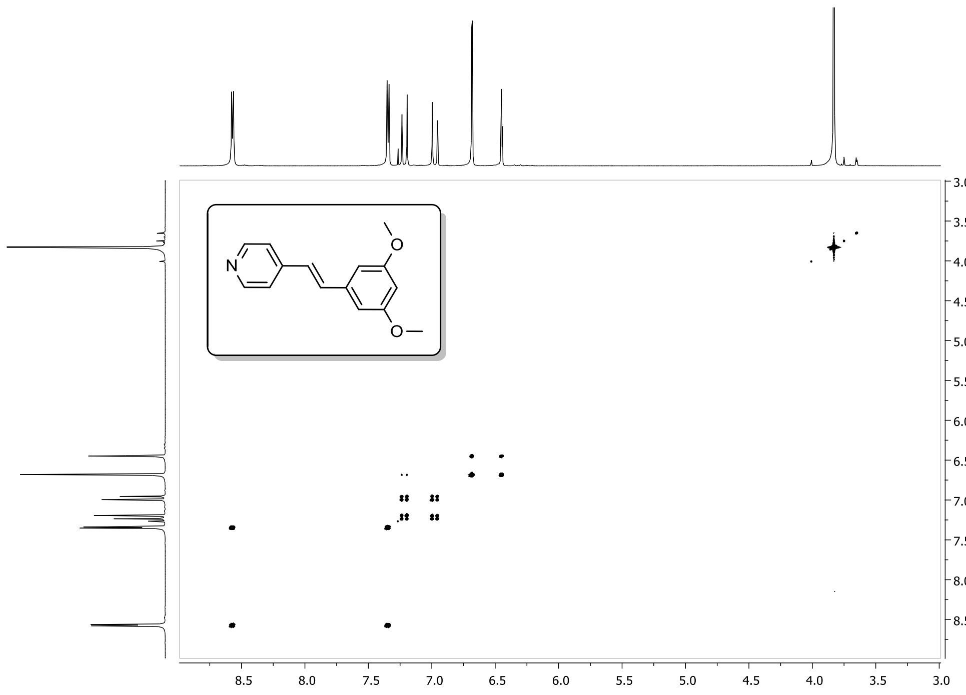

^{1}H-^{13}C HSQC NMR (400 MHz, $CDCl_3$) *(E)*-4-(3, 5-dimethoxystyryl)pyridine (14)

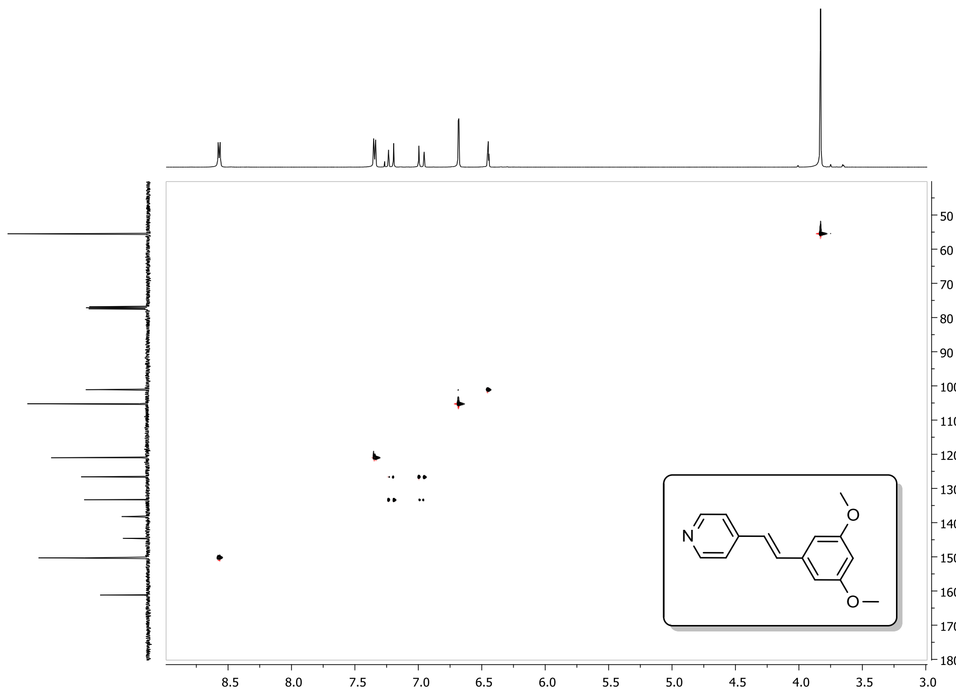

^{1}H-^{13}C HMBC NMR (400 MHz, $CDCl_3$) *(E)*-4-(3, 5-dimethoxystyryl)pyridine (14)

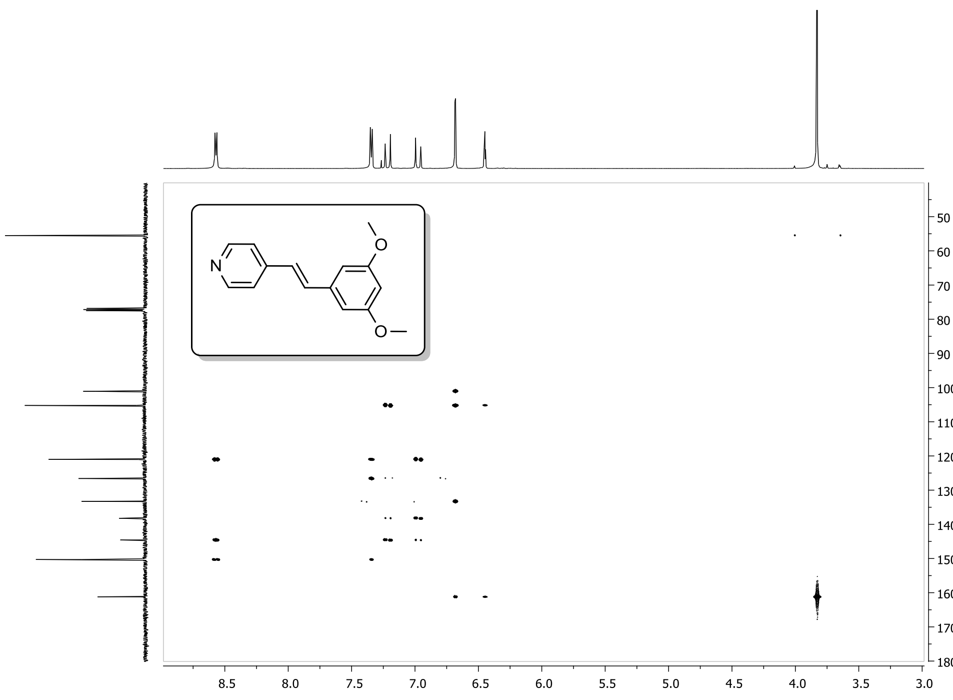

^{1}H NMR (400 MHz, $CDCl_3$) 1-(4-(2,2-diphenylvinyl)phenyl)ethanone (15)

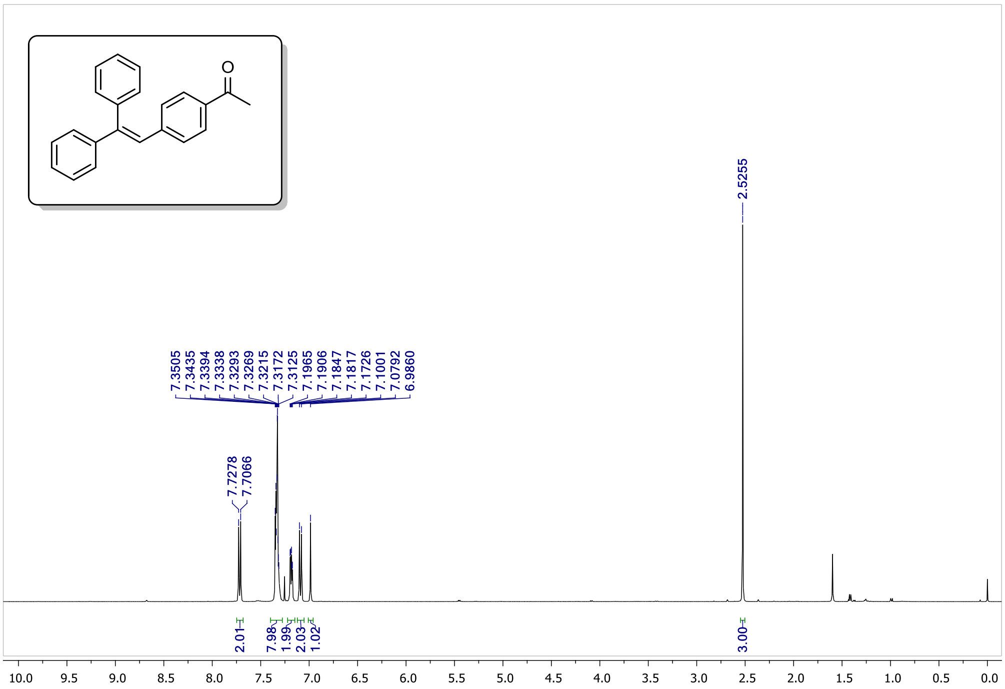

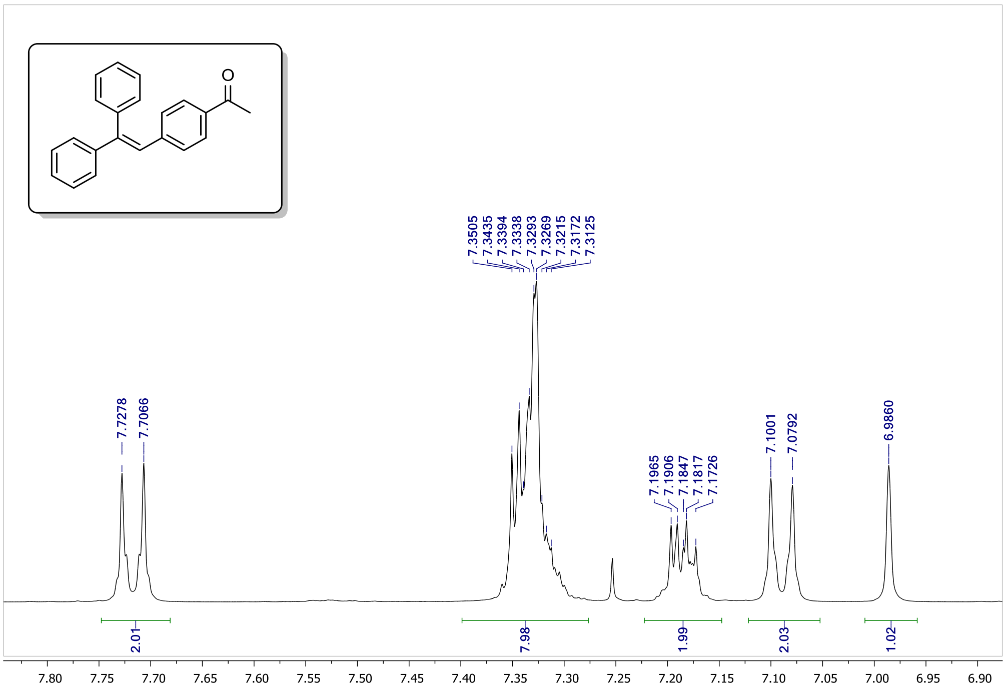

^{13}C NMR (101 MHz, $CDCl_3$) 1-(4-(2,2-diphenylvinyl)phenyl)ethanone (15)

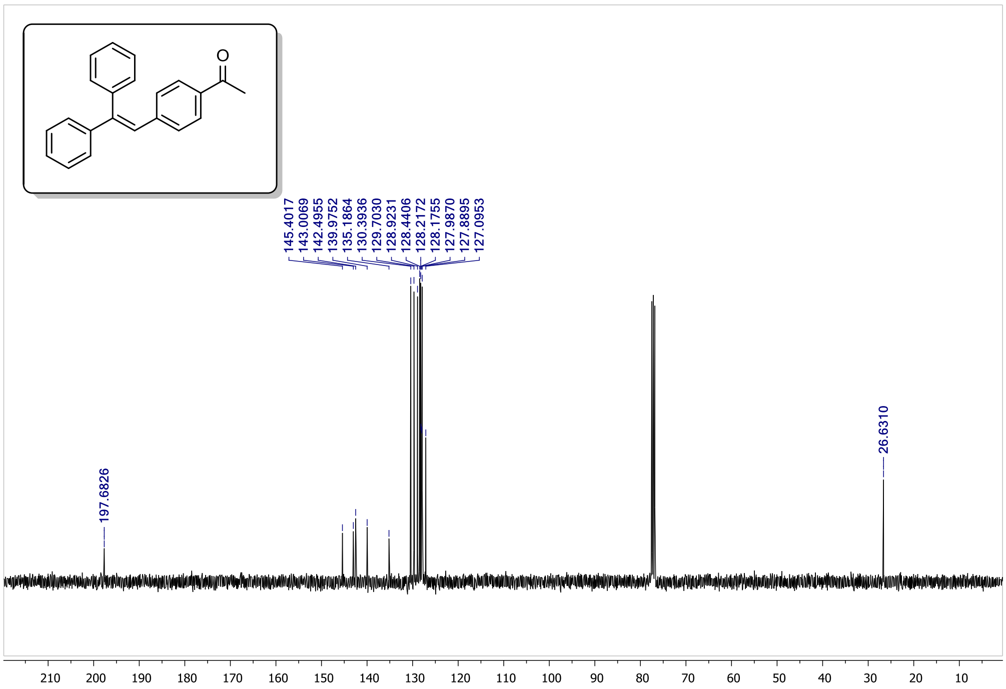

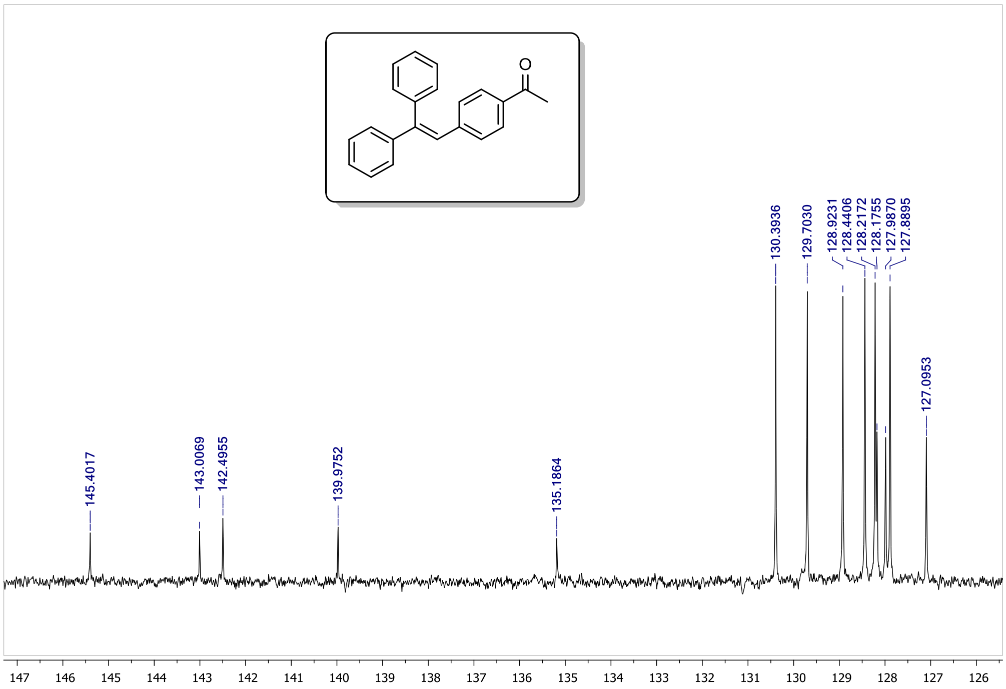

^{1}H-^{1}H COSY NMR (400 MHz, $CDCl_3$) 1-(4-(2,2-diphenylvinyl)phenyl)ethanone (15)

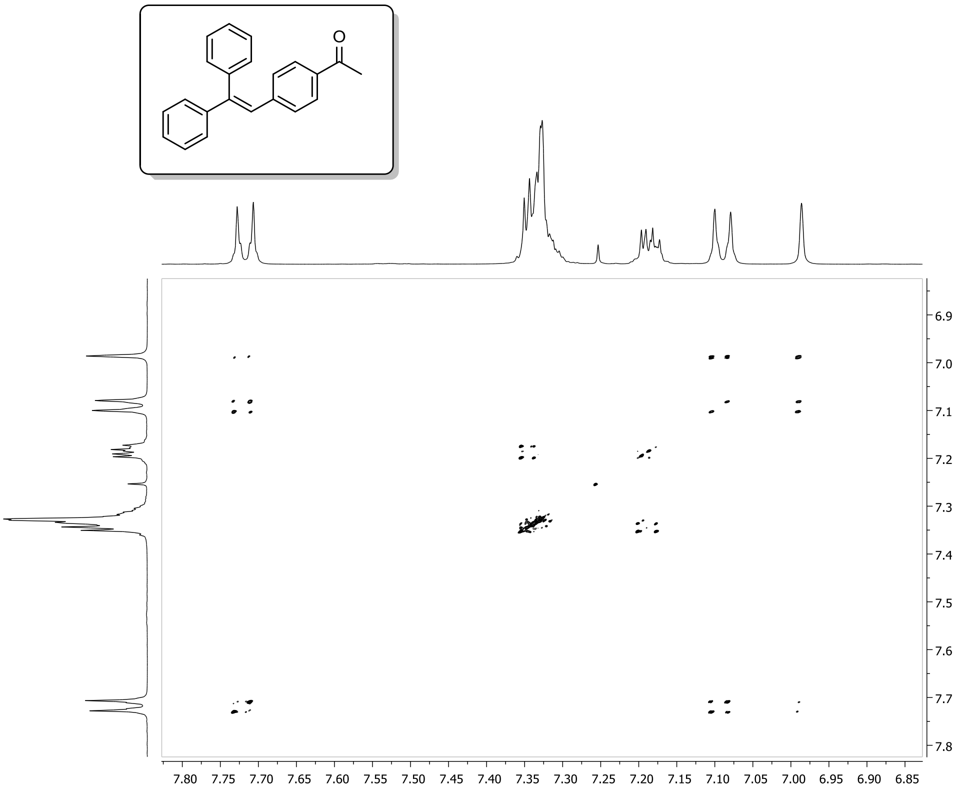

^{1}H-

^{13}C

HSQC NMR (400 MHz, $CDCl_3$) 1-(4-(2,2-diphenylvinyl)phenyl)ethanone (15)

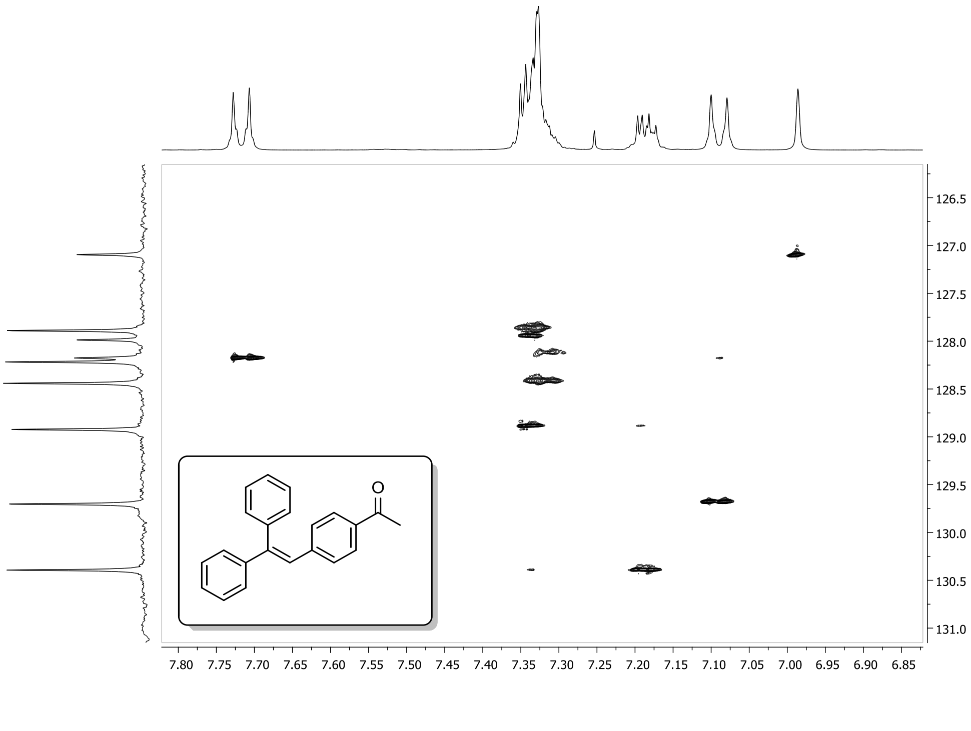

^{1}H-^{13}C HMBC NMR (400 MHz, $CDCl_3$) 1-(4-(2,2-diphenylvinyl)phenyl)ethanone (15)

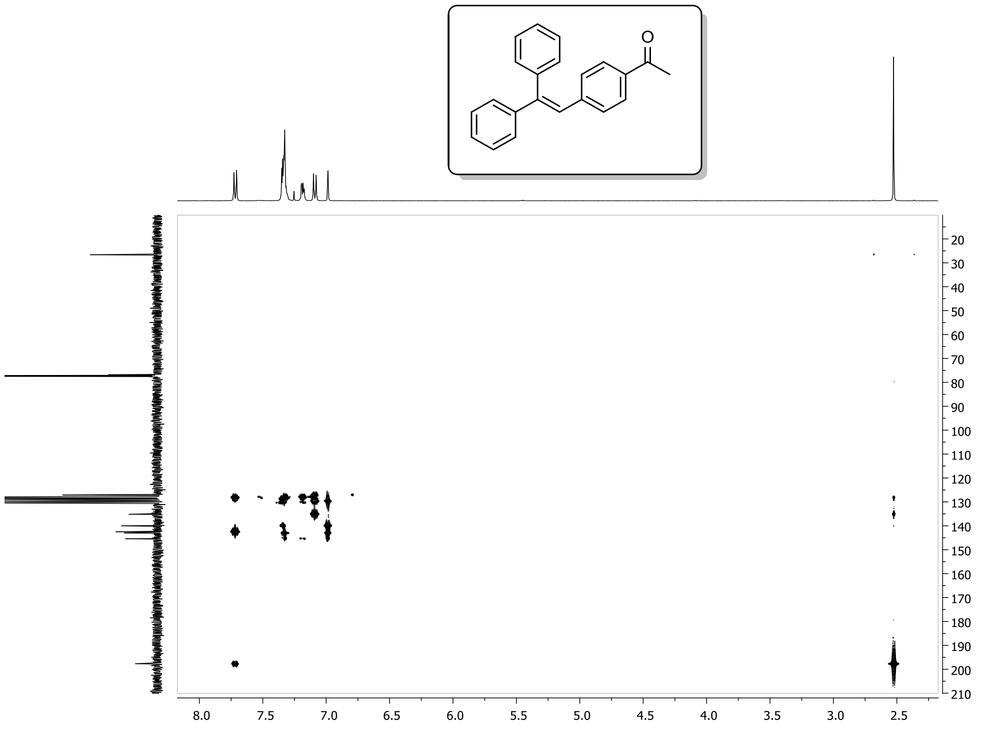

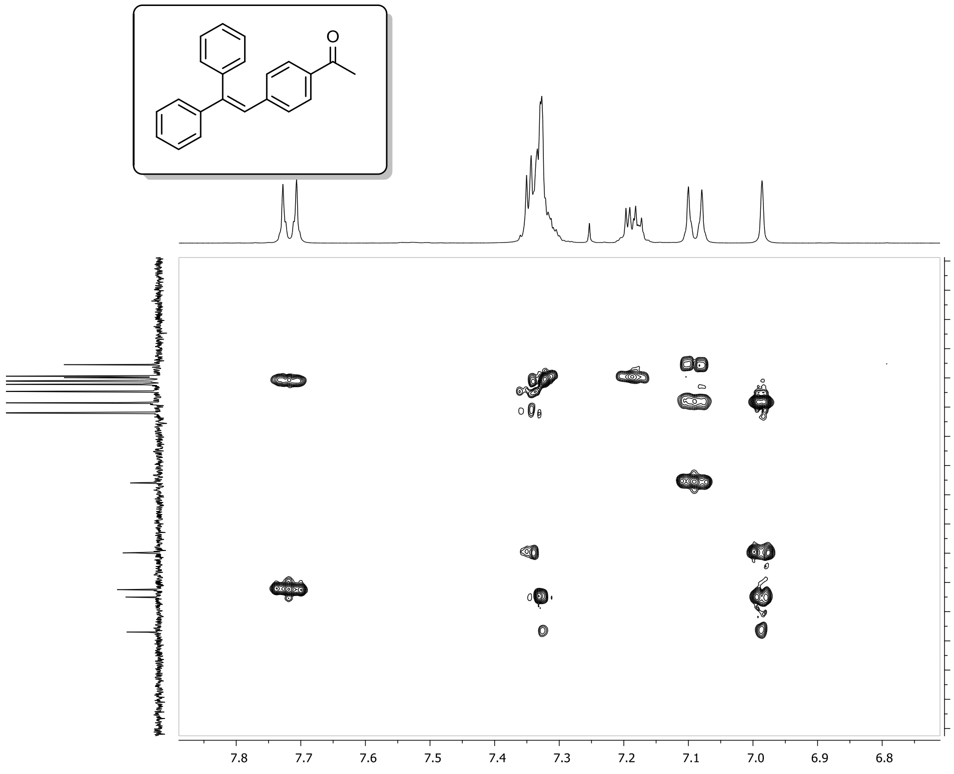

^{1}H NMR (400 MHz, $CDCl_3$) (*E*)-1,1'-(ethene-1,2-diylbis(4,1-phenylene))diethanone (17)

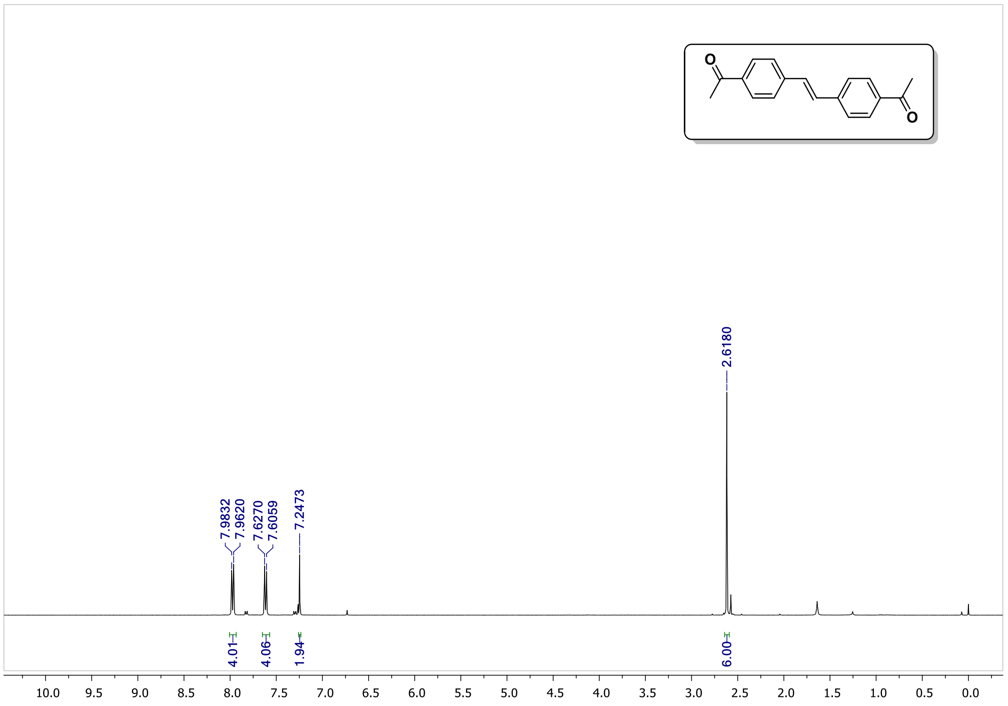

^{13}C NMR (101 MHz, $CDCl_3$) (*E*)-1,1'-(ethene-1,2-diylbis(4,1-phenylene))diethanone (17)

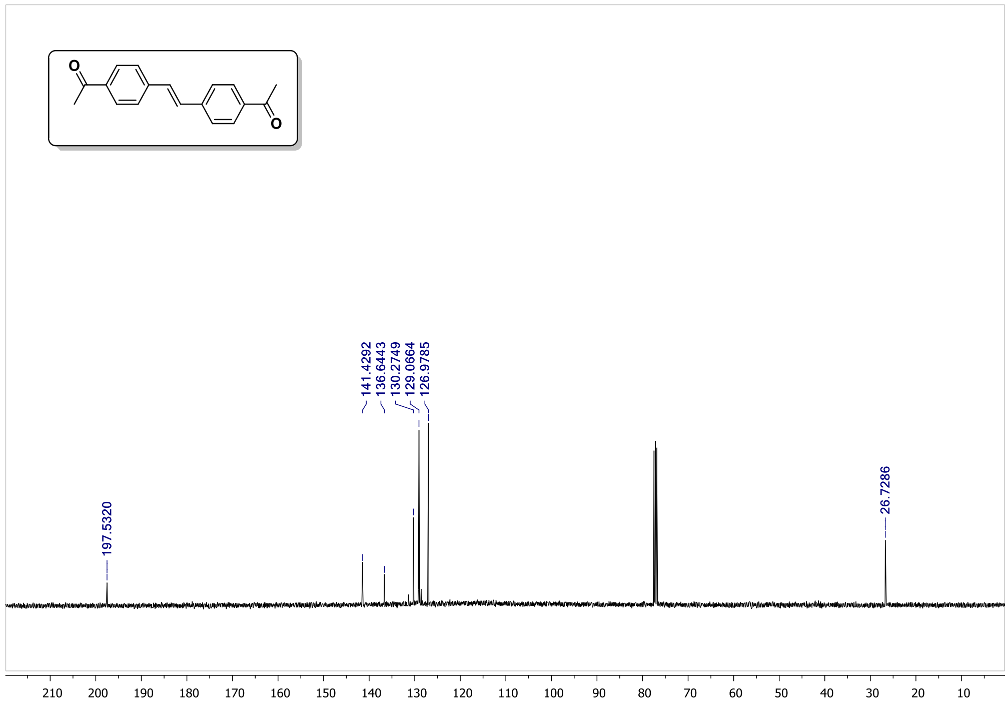

^{1}H-^{1}H COSY NMR (400 MHz, $CDCl_3$) (*E*)-1,1'-(ethene-1,2-diylbis(4,1-phenylene))diethanone (17)

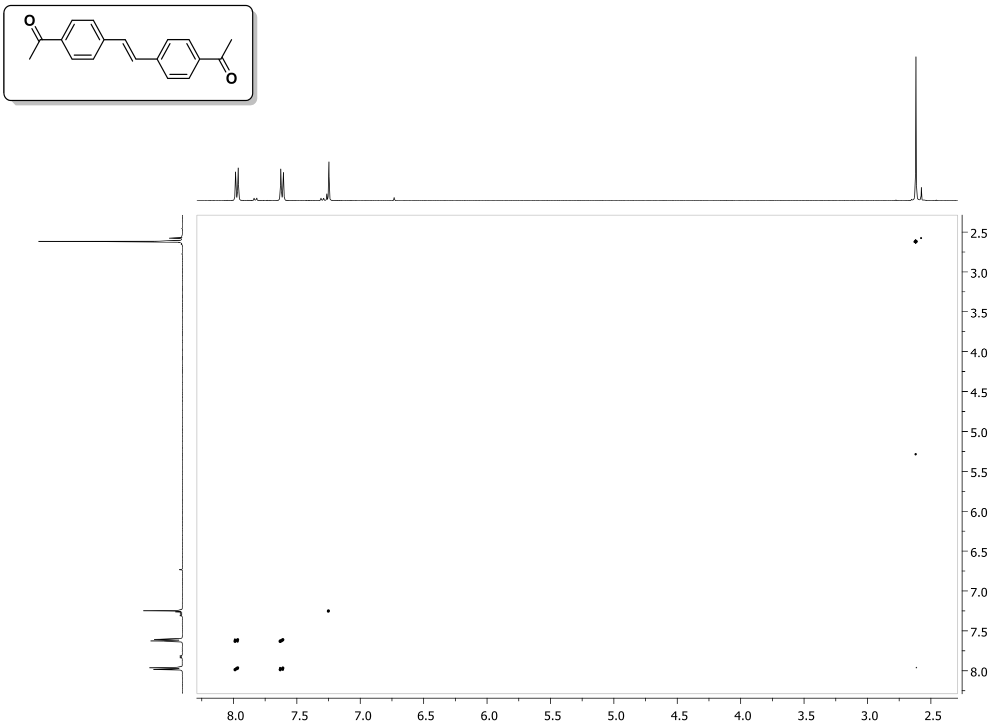

^{1}H-^{13}C HSQC NMR (400 MHz, $CDCl_3$) (*E*)-1,1'-(ethene-1,2-diylbis(4,1-phenylene))diethanone (17)

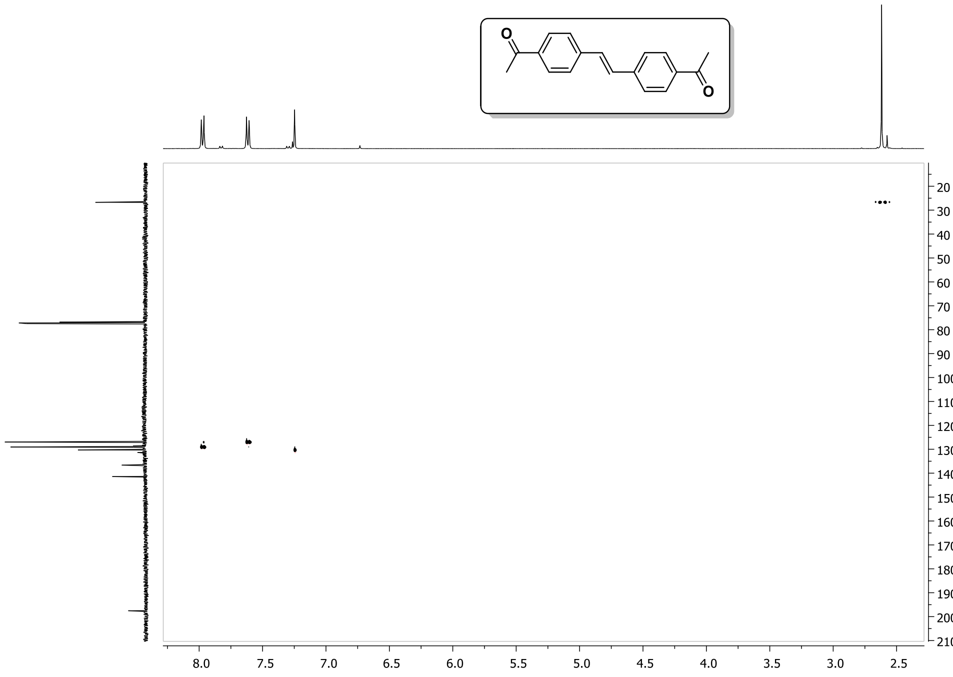

^{1}H-^{13}C HSQC NMR (400 MHz, $CDCl_3$) (*E*)-1,1'-(ethene-1,2-diylbis(4,1-phenylene))diethanone (17)

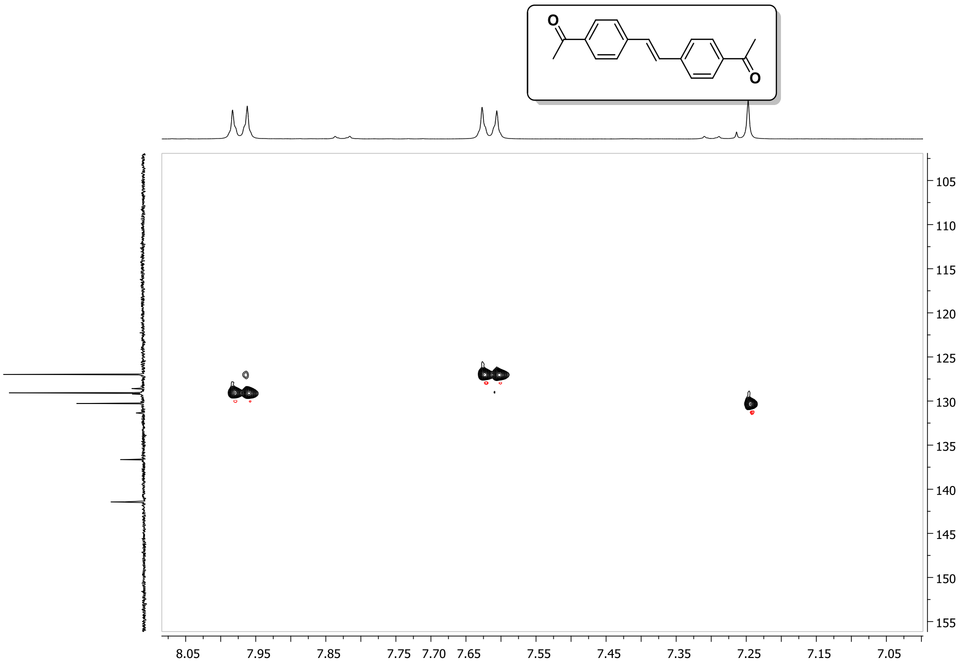

^{1}H-^{13}C HMBC NMR (400 MHz, $CDCl_3$) (*E*)-1,1'-(ethene-1,2-diylbis(4,1-phenylene))diethanone (17)

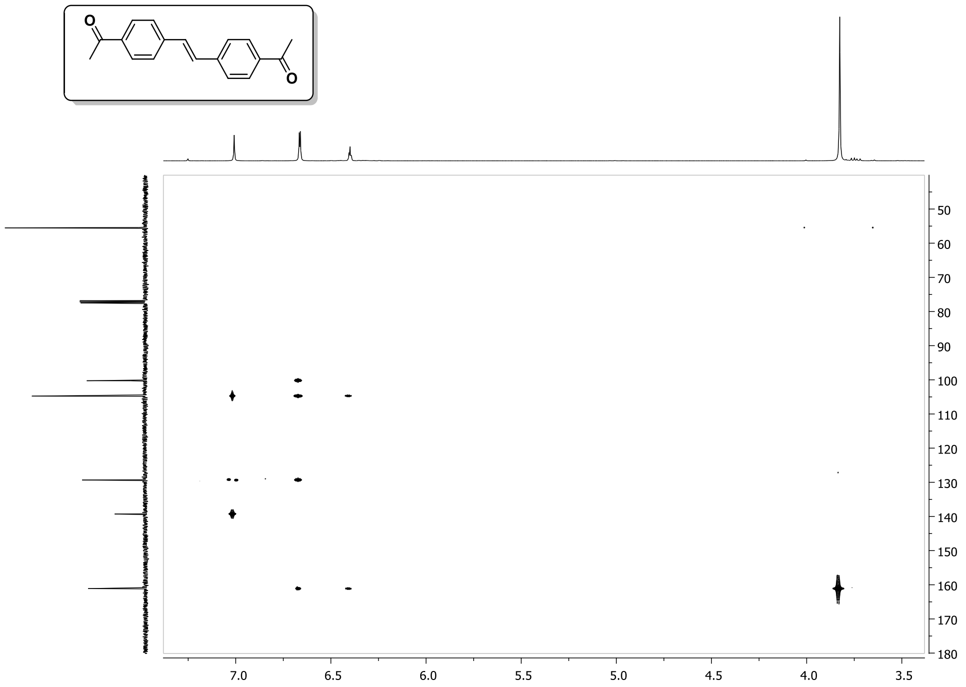

^{1}H NMR (400 MHz, $CDCl_3$) (*E*)-1,2-bis(3,5-dimethoxyphenyl)ethane (18)

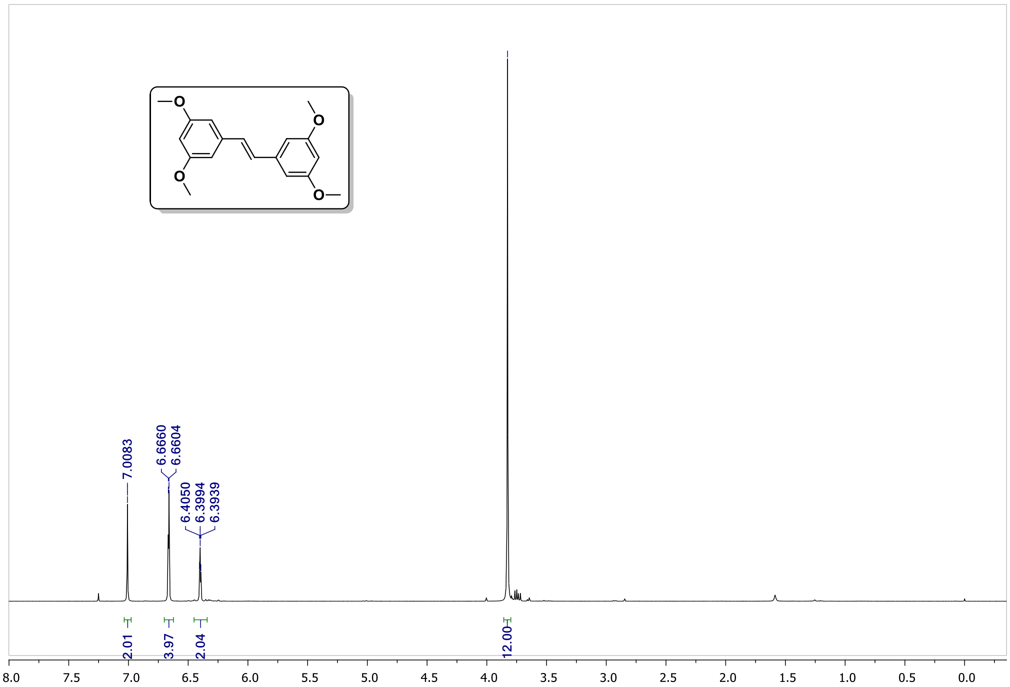

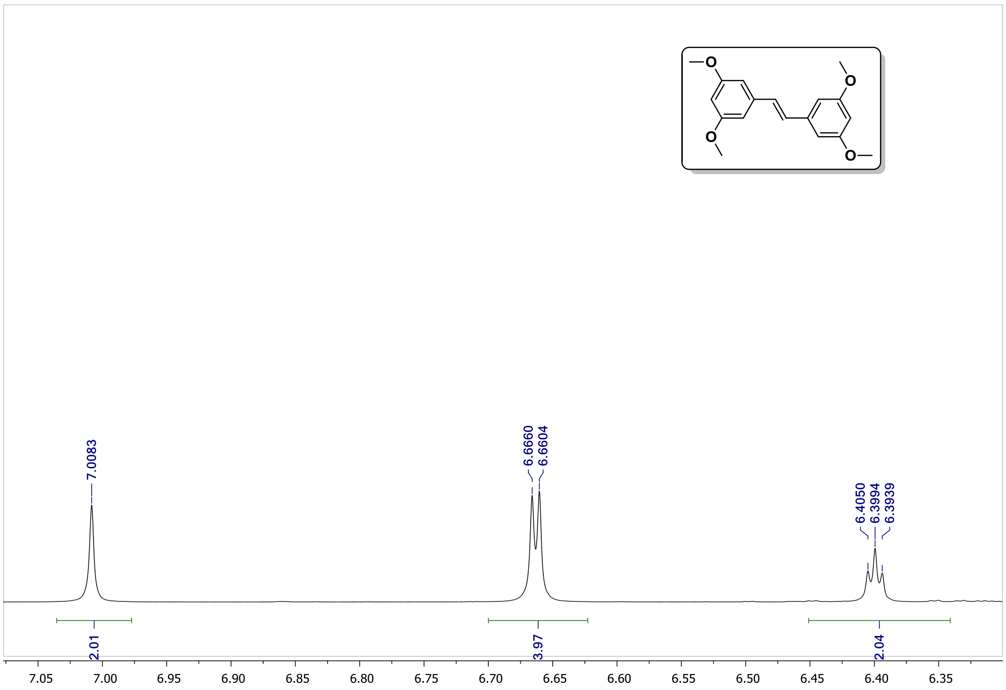

^{13}C NMR (101 MHz, CDCl$_3$) (*E*)-1,2-bis(3,5-dimethoxyphenyl)ethane (18)

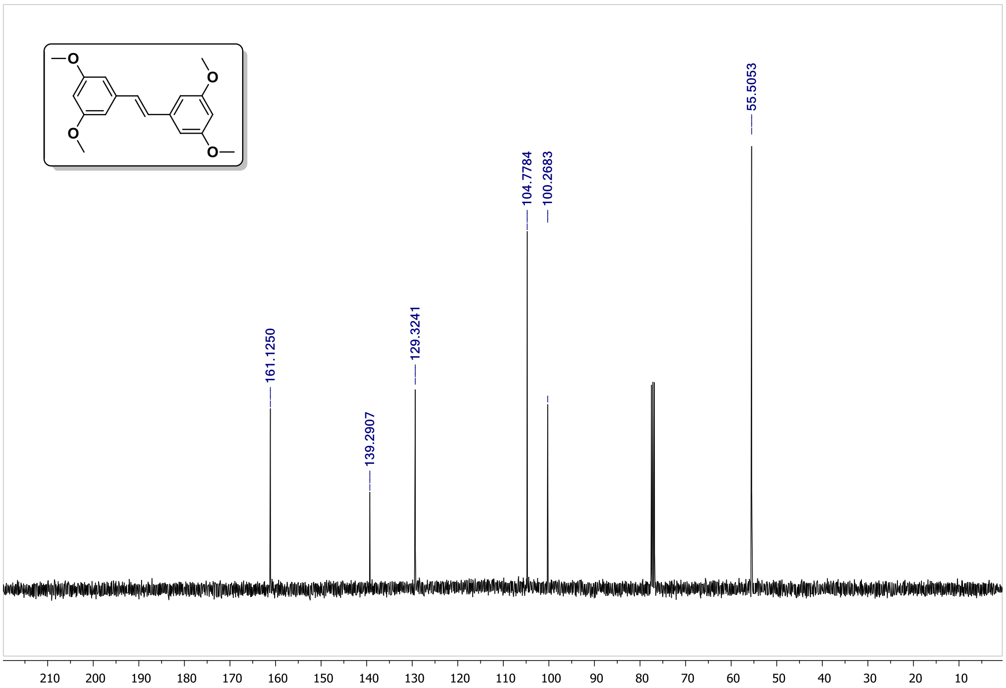

^{1}H-^{1}H COSY NMR (400 MHz, CDCl$_3$) (*E*)-1,2-bis(3,5-dimethoxyphenyl)ethane (18)

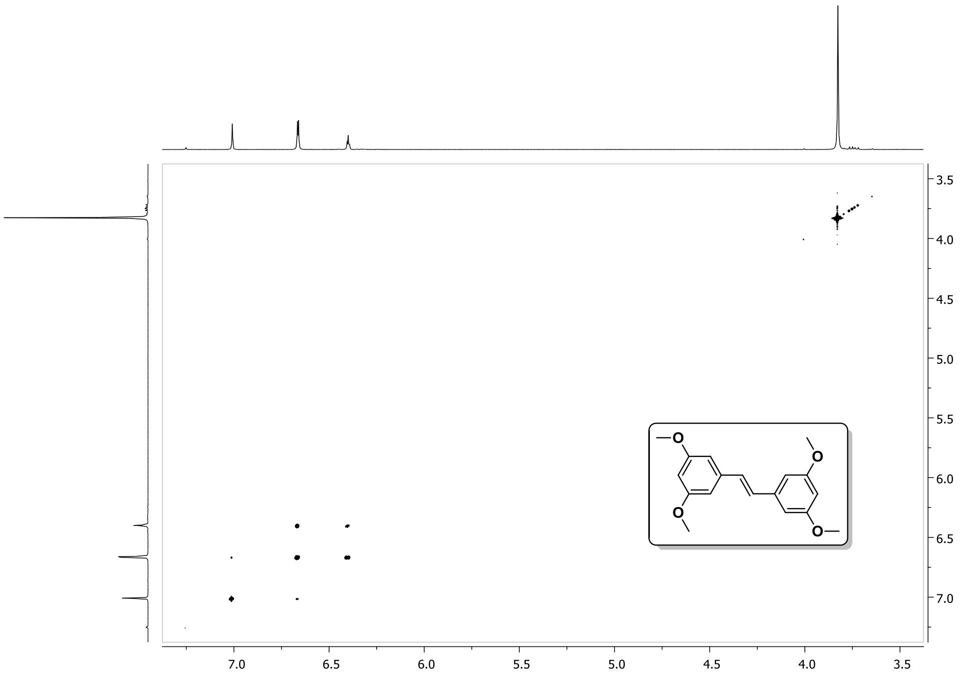

^{1}H-^{13}C HSQC NMR (400 MHz, $CDCl_3$) (*E*)-1,2-bis(3,5-dimethoxyphenyl)ethane (18)

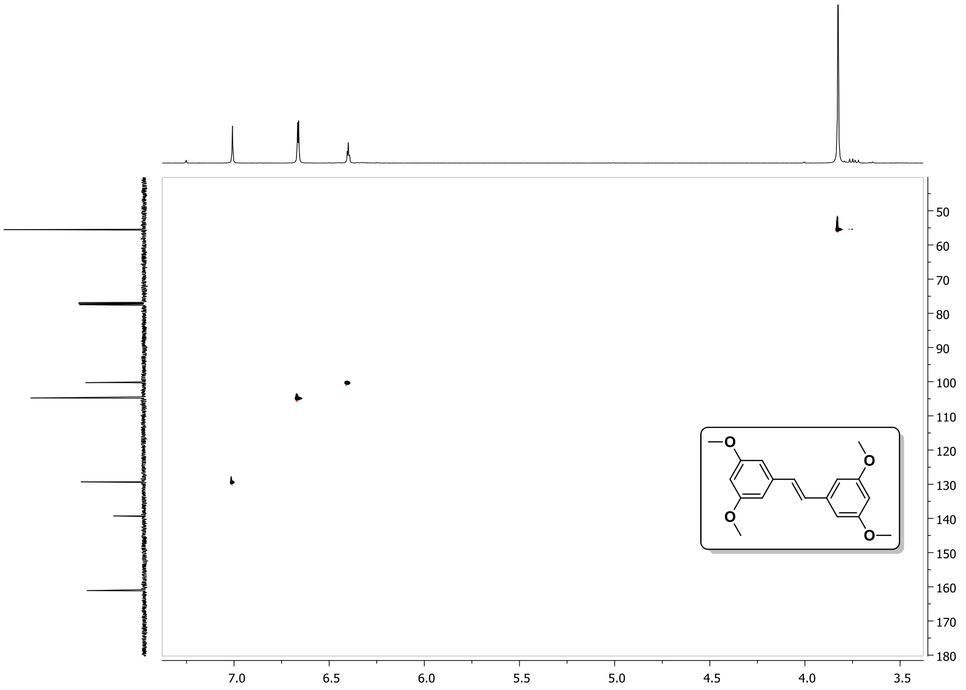

^{1}H-^{13}C HMBC NMR (400 MHz, $CDCl_3$) (*E*)-1,2-bis(3,5-dimethoxyphenyl)ethane (18)

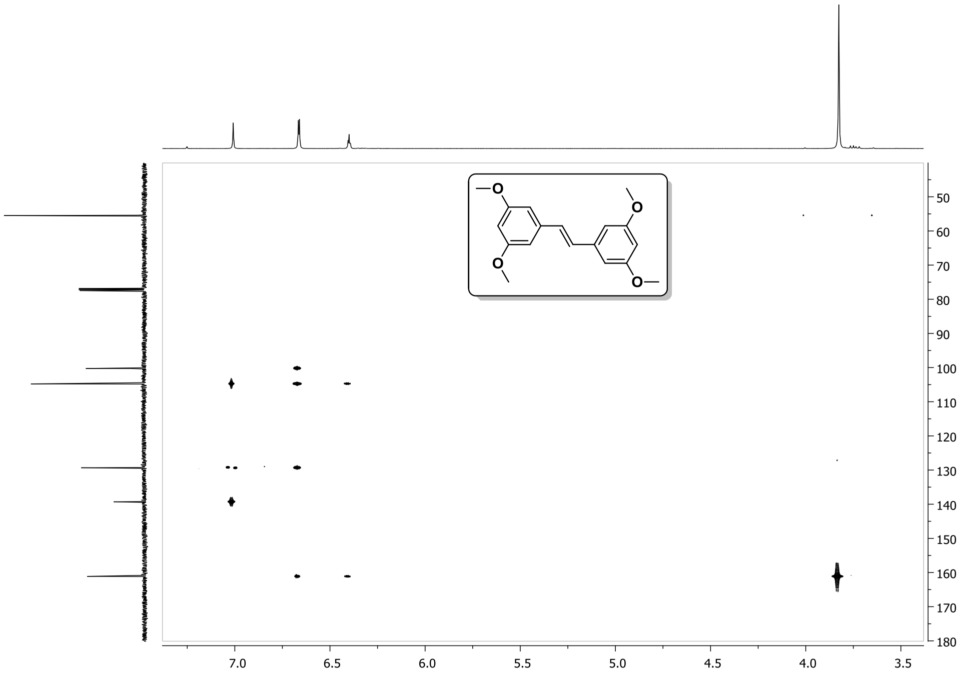

^{1}H NMR (400 MHz, $CDCl_3$) *(E)*-4-(3,5-dimethoxystyryl)phenol (19)

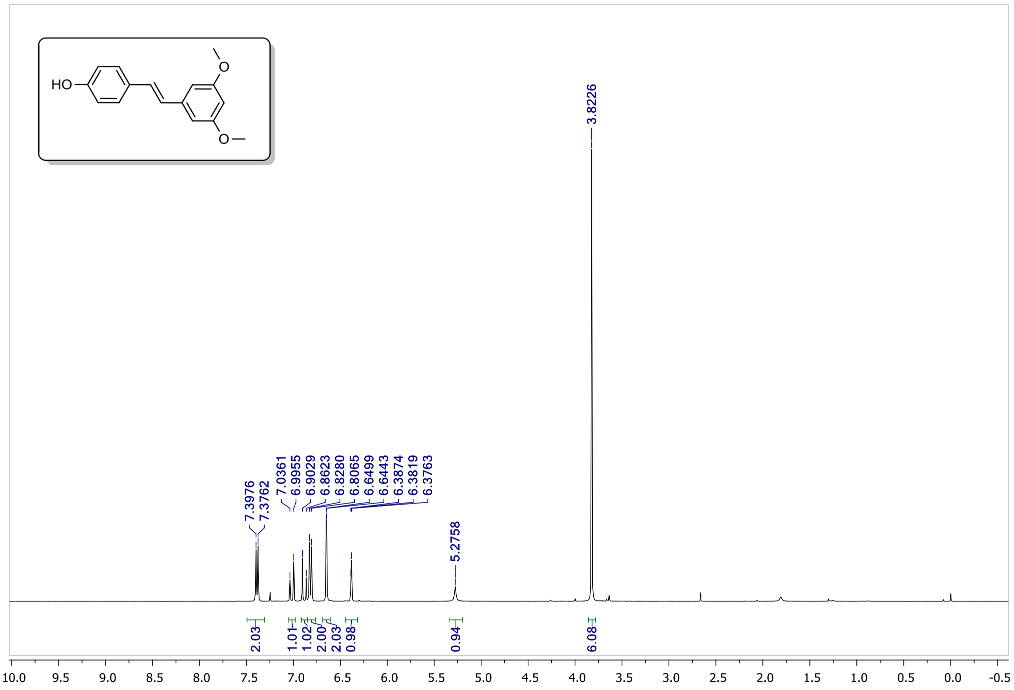

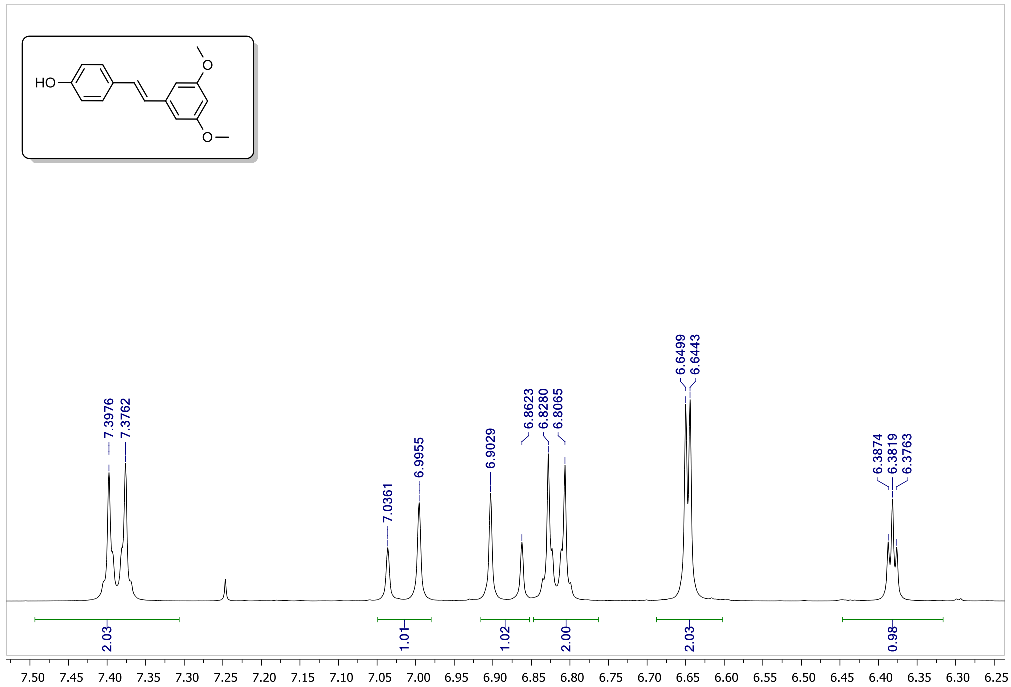

^{13}C NMR (101 MHz, CDCl$_3$) *(E)*-4-(3,5-dimethoxystyryl)phenol (19)

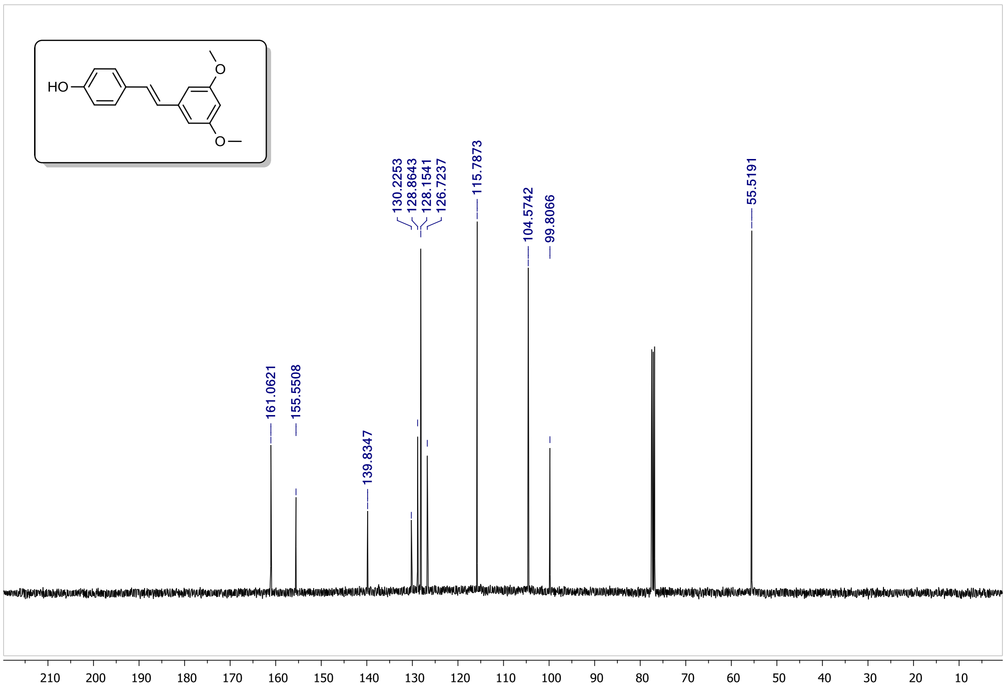

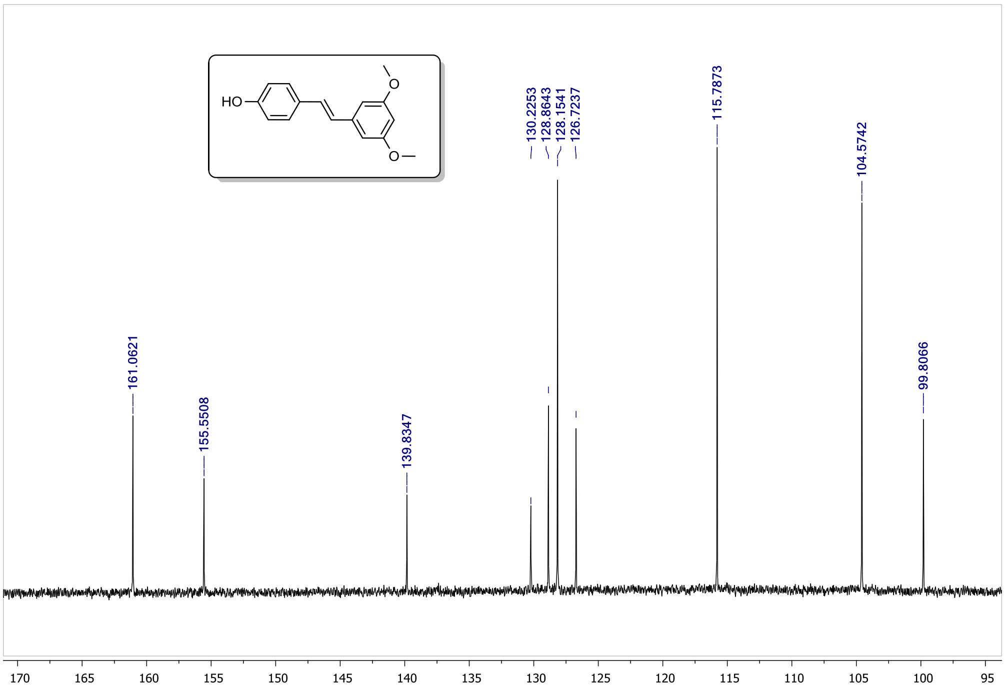

^{1}H-^{1}H COSY NMR (400 MHz, $CDCl_3$) *(E)*-4-(3,5-dimethoxystyryl)phenol (19)

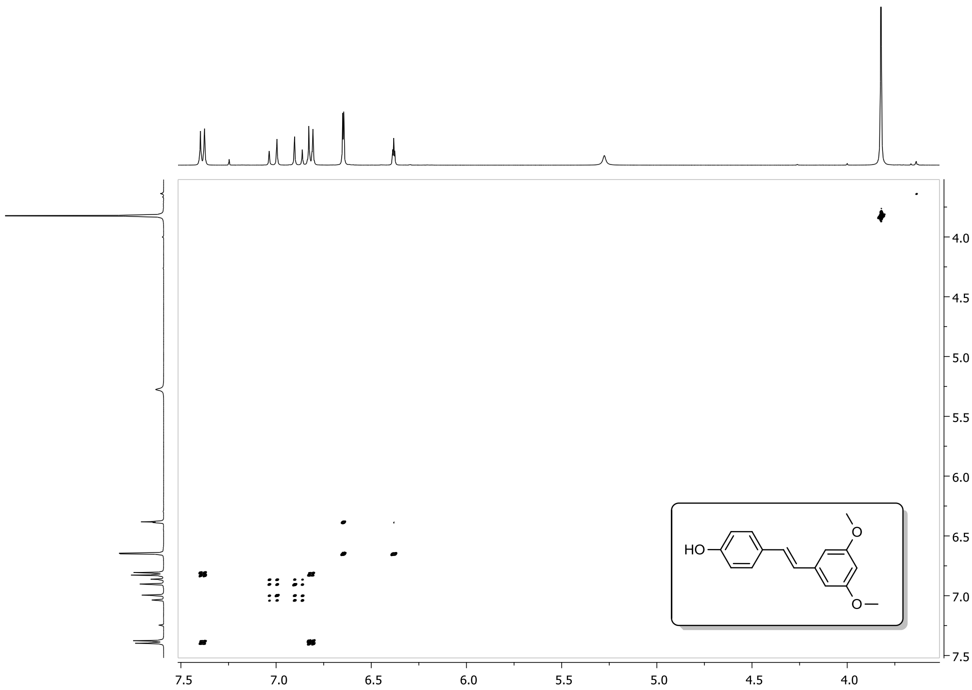

^{1}H-^{13}C HSQC NMR (400 MHz, $CDCl_3$) *(E)*-4-(3,5-dimethoxystyryl)phenol (19)

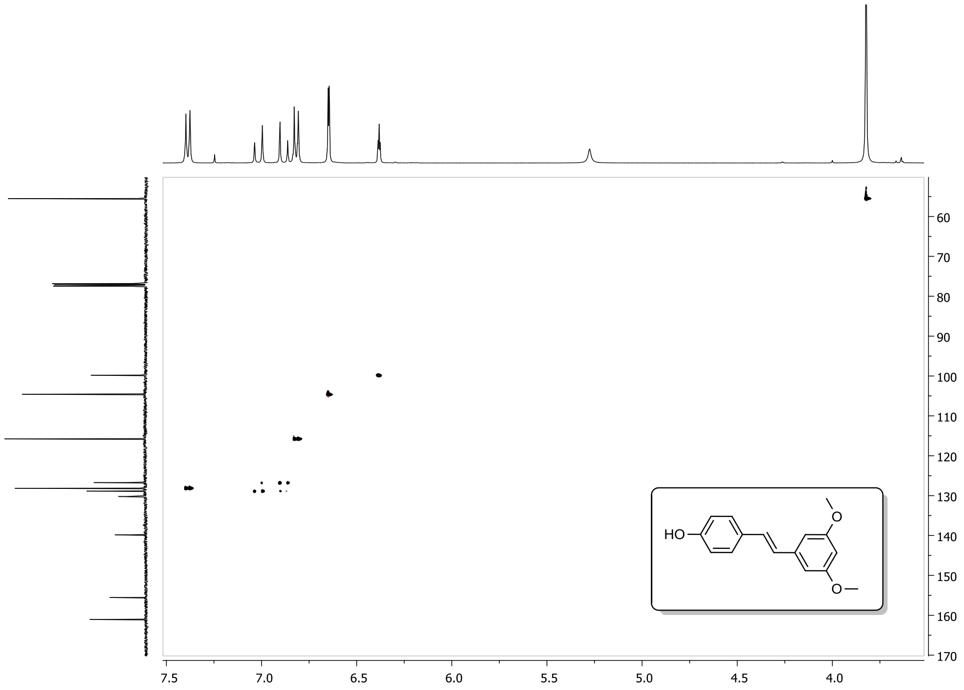

^{1}H-^{13}C HMBC NMR (400 MHz, $CDCl_3$) *(E)*-4-(3,5-dimethoxystyryl)phenol (19)

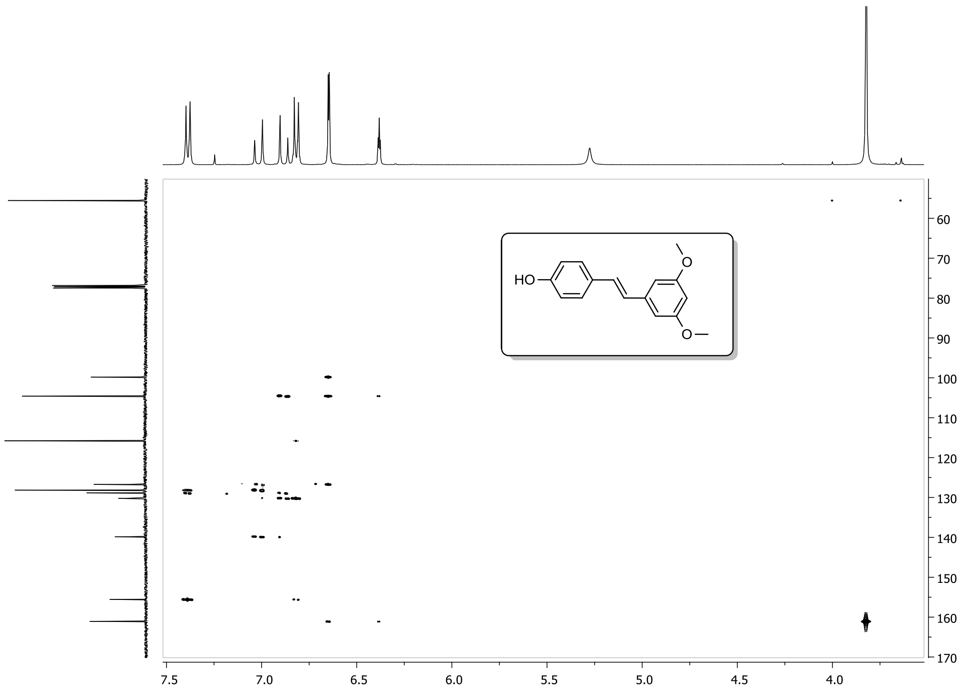

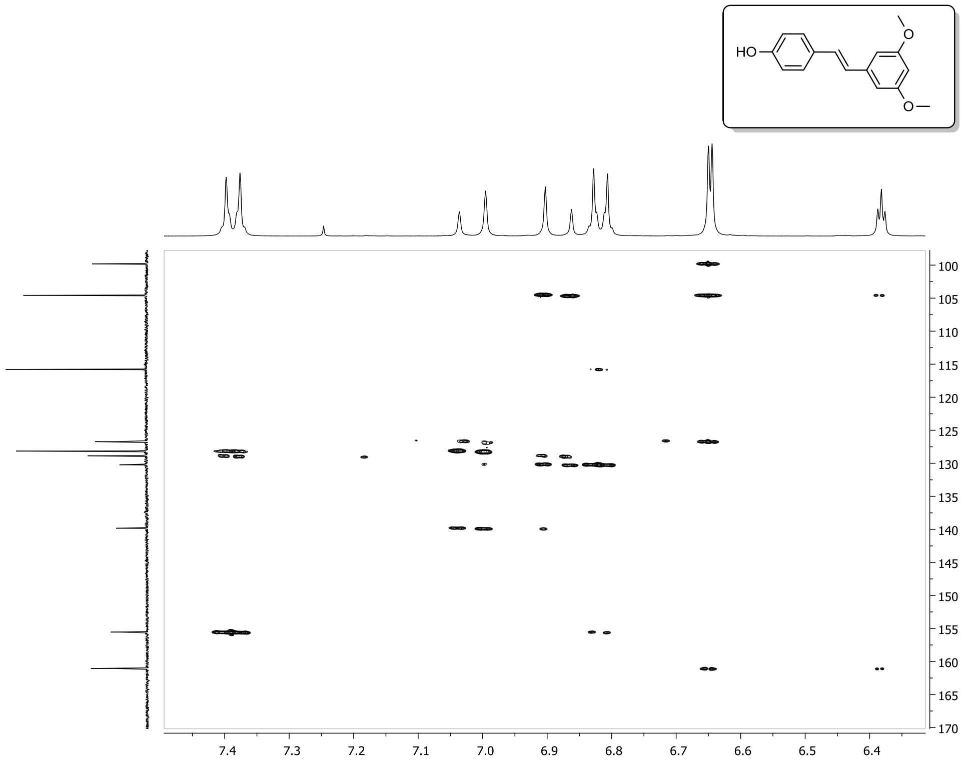

4. References

1 E. Alacid, C. Nájera, *J. Org. Chem.* **2009**, *74*, 8191.

2 M. Planellas, Y. Moglie, F. Alonso, M. Yus, R. Pleixats, A. Shafir, *Eur. J. Org. Chem.* **2014,** 3001.

3 J. F. Guastavino, M. E. Budén, R. A. Rossi, *J. Org. Chem.* **2014**, *79*, 9104.

4 X. Cui, Z. Li, C-Z. Tao, Y. Xu, J. Li, L. Liu, Q-X. Guo, *Org. Lett.* **2006**, *8*, 2467.

5 C. Lu, Y. Guo, J. Li, M. Yao, Q. Liao, Z. Xie, X. Li, *Bioorg. Med. Chem. Lett*. **2012**, *22*, 7683.

6 K. Itami, T. Nokami, Y. Ishimura, K. Mitsudo, T. Kamei, J. Yoshida, *J. Am. Chem. Soc.* **2001**, *123***,** 11577.

7 A. Gordillo, J. Forigua, C. López Mardomingo, E. de Jesús. *Organometallics*, **2011**, *30*, 352.

8 H. Diéguez, A. López, V. Domingo, J. Arteaga, J. Dobado, M. Herrador, J. Quílez del Moral, A. Barrero. *J. Am. Chem. Soc*. **2010**, 132, 254.

9 S. Albert, R. Horbach, H. B. Deising, B. Siewert, R. Csuk, *Bioorg. Med. Chem.* **2011**, *19*, 5155.
